# Effect of Swine Glyco-humanized Polyclonal Neutralizing Antibody on Survival and Respiratory Failure in Patients Hospitalized With Severe COVID-19: A Randomized, Placebo-Controlled Trial

**DOI:** 10.1093/ofid/ofad525

**Published:** 2023-10-20

**Authors:** Benjamin Gaborit, Bernard Vanhove, Karine Lacombe, Thomas Guimard, Laurent Hocqueloux, Ludivine Perrier, Vincent Dubee, Virginie Ferre, Celine Bressollette, Régis Josien, Aurélie Le Thuaut, Marie-Anne Vibet, Alexandra Jobert, Eric Dailly, Florence Ader, Sophie Brouard, Odile Duvaux, François Raffi, for the POLYCOR study group, Benjamin Gaborit, François Raffi, Maeva Lefebvre, Charlotte Biron, Raphaël Lecomte, Cécile Braudeau, Marie Chauveau, Eric Dailly, Colin Deschanvres, Matthieu Gregoire, Anne-sophie Lecomte, Laurent Flet, Martine Tching-Sin, Eugenie Clapeau, Jérémie Orain, Morgane Le Bras, Alexandre Duval, Isabelle Portier, Clara Mora, Anne-Sophie Boureau, Etienne Seronie-Doutriaux, Agnès Rouaud, Pamela Hublain, Laurence Le Jumeau De Kergaradec, Carole Agasse, Vivien Danielo, Megguy Bernard, Régine Valero, Karine Lacombe, Diane Bollens, Thibault Chiarabini, Nadia Valin, Patrick Ingiliz, Zineb Ouazene, Bénédicte Lefebvre, François Lecardonnel, Christian Tran, Raynald Feliho, Manuela Le Cam, Julie Lamarque, Jean-Luc Lagneau, Cyrielle Letaillandier, Anne Daguenel-Nguyen, Clémentine Mayala-Kanda, Djeneba Fofana, Arianna Fiorentino, Thomas Guimard, Yves Bleher, Jean-Luc Esnault, Dominique Merrien, Blandine Le Claire, Marine Morrier, Delphine Boucher, Romain Lamberet, Clémentine Coudon, Romain Decours, Hélène Durand, Armelle Pegeot, Edwige Migne, Hélène Pelerin, Yannick Poirier, Laurent Hocqueloux, Thierry Prazuck, Barbara De Dieuleveult, Pierre Plocco, Jérôme Guinard, Elisa Demonchy, Eric Cua, Edouard Devaud, Stanislas Harent, Marion Parisey, Céleste Lambert, Elise Gobin, Julien Manson, Pierre Pasquier, Pascale Martres, Patricia Kessedjian, Hikombo Hitoto, Nicolas Crochette, Lucia Perez-Grandiere, Jean-Baptiste Laine, Arnaud Salmon-Rousseau, Guillaume Cosseron, Sophie Blanchi, Florence Ader, Valérie Galvan, Alexia Moulin, Corinne Brochier, Julianne Oddone, Maude Bouscambert-Duchamp, Firouzé Bani-Sadr, Yohan N’guyen, Maxime Hentzien, Cédric Castex, Philippe Benoit, Véronique Brodard, Didier Laureillard, Albert Sotto, Paul Loubet, Aurélie Martin, Régine Doncesco, Julien Mazet, Ian Soulairol, Robin Stephan, François Goehringer, Nathalie Thilly, Michel Prevot, Hélène Jeulin, Jean-Philippe Talarmin, Lydie Khatchatourian, Nadia Saidan, Brice Guerpillon, Pascaline Rameau, Nicolas Cassou, Thomas Briand, Florence Le Gall, Elodie Le Breton, Cédric Joseph, Sandrine Soriot-Thomas, Claire Andrejak, Jean-Philippe Lanoix, Sophie Boddaert, Sandrine Castelain, Vincent Pestre, Juliette Woessner, Sophie Bayle, Stéphanie Branger, Christine Christides, Philippe Bielefeld, Adèle Lacroix, Roselyne Pillard-Gagliano, Isabelle Trinh, Pierre Lafitte, Guillermo Giordano, Malena Finello, Ignacio Ledesma, Gustavo Martini, Benjamin Delafontaine, Iris Corus, Pierre Baby, Emilie Catherinot, Céline Goyard, Simon Chauveau, Jad Choucair, Beatrice D’urso, Marie Da Silva Costa, Lucie Le Meur, Marc Vasse, Tiffany Pascreau, Eric Farfour, Benjamin Dervieux, C Charlotte Kaeuffer, François Danion, Yves Hansmann, Nicolas Lefebvre, Yvon Ruch, Axel Ursenbach, Catherine Schmidt-Mutter, Muhtadi Suliman, Anne Hutt, Guillaume Becker, Elodie Laugel, Sophie Bayer, Vincent Dubee, Rafael Mahieu, Valérie Daniel, Caroline Lefeuvre, Alexandra Ducancelle, Jean-Charles Gagnard, Abolfzl Mohebbi, Mélanie Dehais, Sophie Raccah, Anne-Lise Pouliquen, Alison Klasen, Emmanuel Forestier, Marie-Christine Carret, Severine Liardot, Jérôme Grosjean, Jean-François Faucher, Josselin Brisset, Anne Cypierre, Hélène Durox, Pauline Pinet, Sophie Ducroix-Roubertou, Claire Genet, Christine Vallejo, Sébastien Hantz, Marie Gousseff, Antoine Merlet, Sébastien Bigot, Marion Girard De Courtilles, Pascal Pouedras, Delphine Lariviere, Renaud Verdon, Sylvie Dargere, Jocelyn Michon, Anna Fournier, Sylvie Brucato, Séverine Gautier, Cécile Valentin, Anne Ricci, Antoine Alix, Flore Lacassin-Beller, Sophie Rousseau, Jérôme Dimet, Anne-Hélène Boivin, Maylis Larregle, Guillaume Rousseau, Ady Assaf, Fanny Vuotto, Karine Faure, Camille Joachim, Laurence Bocket, Kévin Diallo, Jessy Saffore, Isabelle Madeline, Pauline Chabanon, Nathalie Allou, Elisabeth Fernandes, Anne-Sophie Gruliere, Elisabeth Botelho-Nevers, Amandine Gagneux-Brunon, Véronique Ronat, Nadine Casimir, Sylvie Pillet, Frédérique Bertholon, Gilles Pialloux, Marwa Bachir Elrufaai, Ruxandra Calin, Pélagie Thibaut, Fatima Tendjaoui, Julie Fillon, Laurence Morand-Joubert, Marc-Olivier Vareil, Heidi Wille, Philippe Menager, Hugues Cordel, Youssouf Mohammed-Kassim, Vanessa Rathouin, Ségolène Brichler, André Cabie, Mélanie Lehoux, Karine Guitteaud, Karamba Sylla, Bastien Bigeard, Valentine Campana, Isabelle Calmont, Jean-Louis Lamaignere, Marine Deppenweiller, Christophe Padoin, Marine Thilbault, Laurence Fagour, Fatiha Najioullah, Isabelle Komla-Soukha, Mariam Roncato-Saberan, Martin Martinot, Mahsa Mohseni Zadeh, Simon Gravier, Ciprian Ion, Damien Kayser, Anne Schieber Pachart, Magali Eyriey, Anaïs Henric, Jean Daniel Kaiser, Dominique De Briel, Odile Duvaux, Gwenaëlle Evanno, Pierre-Joseph Royer, Juliette Rousse, Carine Ciron, Elsa Lhériteau, Gaëtane Rouvray, Alan Mougeolle, Auriane Rillet, Sophie Despons, Edwige Mevel, Françoise Shneiker, Régis Josien, Cécile Braudeau, Sophie Brouard, Hoa le Mai, Elise Appau-Danquah, Amélie Daniel, Virginie Grybek, David Gueneau, Marion Gautier, Joëlle Martin-Gauthier, Emily Rebouilleau, Joseph Herault, Tanguy Roman, Sorady Plantard, Patrice Chauveau, Anne Chiffoleau, Stéphanie Thauvin, Laurent Flet, Martine Tching-Sin, Eugenie Clapeau, Isabelle Charreau, Bruno Hoen, Caroline Solas-Chesneau, Astrid Vabret

**Affiliations:** Nantes Université, CHU Nantes, INSERM, Department of Infectious Diseases, Nantes, France; Nantes Université, CHU Nantes, INSERM, Center for Research in Transplantation and Translational Immunology, Nantes, France; Xenothera, Nantes, France; INSERM, AP-HP, Hôpital Saint-Antoine, Service des Maladies Infectieuses et Tropicales, Institut Pierre Louis d'Epidémiologie et de Santé Publique, Sorbonne Université, Paris, France; Infectious Diseases and Emergency Department, Centre Hospitalier de La Roche sur Yon, La Roche sur Yon, France; CHU d’Orléans, Department of Infectious Diseases, Orleans, France; Nantes Université, CHU Nantes, Sponsor Department, Direction de la Recherche et de l’Innovation, Nantes, France; Service de Maladies Infectieuses et Tropicales, Centre Hospitalier Universitaire d'Angers, Angers, France; Univ Angers, Nantes Université, INSERM, Immunology and New Concepts in ImmunoTherapy, INCIT, Angers, France; Nantes Université, CHU Nantes, Virology Laboratory, Nantes, France; Nantes Université, CHU Nantes, Virology Laboratory, Nantes, France; Nantes Université, CHU Nantes, INSERM, Center for Research in Transplantation and Translational Immunology, Nantes, France; Nantes Université, CHU Nantes, Laboratoire d’Immunologie, CIMNA, Nantes, France; Nantes Université, CHU Nantes, Sponsor Department, Direction de la Recherche et de l’Innovation, Nantes, France; Nantes Université, CHU Nantes, Plateforme de Méthodologie et Biostatistique, Direction de la Recherche et de l’Innovation, Nantes, France; Nantes Université, CHU Nantes, Sponsor Department, Direction de la Recherche et de l’Innovation, Nantes, France; Nantes Université, CHU Nantes, Plateforme de Méthodologie et Biostatistique, Direction de la Recherche et de l’Innovation, Nantes, France; Nantes Université, CHU Nantes, Sponsor Department, Direction de la Recherche et de l’Innovation, Nantes, France; Nantes Université, CHU Nantes, UMR 1246 MethodS in Patients-centered outcomes and HEalth Research,” SPHERE, Nantes, France; Nantes Université, CHU Nantes, Clinical Pharmacology Department, Nantes, France; CNRS, UMR5308, Ecole Normale Supérieure de Lyon, Centre International de Recherche en Infectiologie (CIRI), Inserm 1111, Université Claude Bernard Lyon 1, Université Lyon, Lyon, France; Département des Maladies Infectieuses et Tropicales, Hospices Civils de Lyon, Lyon, France; Nantes Université, CHU Nantes, INSERM, Center for Research in Transplantation and Translational Immunology, Nantes, France; Xenothera, Nantes, France; Nantes Université, CHU Nantes, INSERM, Department of Infectious Diseases, Nantes, France

**Keywords:** SARS-CoV-2, XAV-19, clinical trial, polyclonal glyco-humanized anti-SARS-CoV-2 antibody

## Abstract

**Background:**

We evaluated the safety and efficacy of XAV-19, an antispike glyco-humanized swine polyclonal neutralizing antibody in patients hospitalized with severe coronavirus disease 2019 (COVID-19).

**Methods:**

This phase 2b clinical trial enrolled adult patients from 34 hospitals in France. Eligible patients had a confirmed diagnosis of severe acute respiratory syndrome coronavirus 2 within 14 days of onset of symptoms that required hospitalization for low-flow oxygen therapy (<6 L/min of oxygen). Patients were randomly assigned to receive a single intravenous infusion of 2 mg/kg of XAV-19 or placebo. The primary end point was the occurrence of death or severe respiratory failure between baseline and day 15.

**Results:**

Between January 12, 2021, and April 16, 2021, 398 patients were enrolled in the study and randomly assigned to XAV-19 or placebo. The modified intention-to-treat population comprised 388 participants who received full perfusion of XAV-19 (199 patients) or placebo (189 patients). The mean (SD) age was 59.8 (12.4) years, 249 (64.2%) individuals were men, and the median time (interquartile range) from symptom onset to enrollment was 9 (7–10) days. There was no statistically significant decrease in the cumulative incidence of death or severe respiratory failure through day 15 in the XAV-19 group vs the placebo group (53/199 [26.6%] vs 48/189 [25.4%]; adjusted risk difference, 0.6%; 95% CI, −6% to 7%; hazard ratio, 1.03; 95% CI, 0.64–1.66; *P* = .90). In the safety population, adverse events were reported in 75.4% of 199 patients in the XAV-19 group and in 76.3% of 190 patients in the placebo group through D29.

**Conclusions:**

Among patients hospitalized with COVID-19 requiring low-flow oxygen therapy, treatment with a single intravenous dose of XAV-19, compared with placebo, did not show a significant difference in terms of disease progression at day 15.

Coronavirus disease 2019 (COVID-19) pneumonia in hospitalized patients often leads to acute respiratory distress syndrome and use of high-flow oxygen, mechanical ventilation, and a high rate of death [1]. In the past 2 years, progress has been made to improve management of the most severe forms of COVID-19, including heparin-based thromboprophylaxis [2], remdesivir [3], glucocorticoids [4], interleukin-6 signaling inhibitors [5], selective inhibitor of Janus kinase (JAK) [6], and neutralizing antibodies [7].

Many neutralizing monoclonal antibody treatments have been developed and have been recommended by the World Health Organization for patients at an early stage of COVID-19 to prevent progression to severe or critical disease [7, 8]. Given the resistance of current SARS-CoV-2 variants to monoclonal antibodies, there is a clinical need for new neutralizing antibodies for management of COVID-19 patients. The advantage of polyclonal antibodies over monoclonal antibodies, which bind to nonoverlapping epitopes, is to reduce neutralization escape by SARS-CoV-2 and mutations in the spike gene [9, 10].

Heterologous animal-derived polyclonal antibodies could be an advantageous approach but raise safety concerns related to the risk of serum sickness [11]. XAV-19 is a purified polyclonal immunoglobulin G (IgG) derived from immunization with the receptor binding domain (RBD) of the SARS-CoV-2 spike in CMAH/GGTA1 double-knockout pigs designed to produce polyclonal glyco-humanized antibodies, leading to improved tolerability for administration in humans [12]. XAV-19 binds multiple target epitopes on SARS-CoV-2 spike, maintains neutralizing activity against the Alpha, Beta, Gamma, Delta, and Omicron variants of concern, and does not induce escape mutations in SARS-CoV-2 [13].

In a phase 2a study conducted in severe COVID-19 hospitalized patients, a single intravenous perfusion of XAV-19 at 2 mg/kg was safe and maintained plasma XAV-19 concentrations above the expected target neutralization concentration for at least 8 days after infusion (estimated half-life of 11.4 days) [14, 15].

We conducted a multicenter, randomized, double-blind, placebo-controlled phase 2b study to investigate the efficacy of XAV-19 in hospitalized patients with severe COVID-19 who required low-flow oxygen support.

## METHODS

### Trial Design and Ethical Considerations

The POLYCOR trial was a multicenter, phase 2b, double-blind, placebo-controlled, randomized clinical trial conducted at 34 sites in France. Details of the trial design have been reported previously [14] and are available in the trial protocol and the statistical analysis plan (Supplementary Data 1 & 2).

### Patient Consent

This trial was conducted in accordance with the Good Clinical Practice guidelines of the International Council for Harmonization E6 and the principles of the Declaration of Helsinki. The protocol was reviewed by the French National Agency for Medicines and Health Products’ Safety (ANSM MEDMSANAT-2020-12-00243_2020-002574-27, approval 12/28/2020) and approved by the Ethics Committee CPP Ouest VI (Brest, France, approval #20.06.15.31.306, CPP reference 1305, 01/08/2021) and was sponsored by the research department of Nantes University Hospital. Written informed consent was obtained from all participants at the time of enrollment.

### Patients

Patients aged 18 years or older who were hospitalized with severe COVID-19, as confirmed by positive polymerase chain reaction (PCR) assay of respiratory samples, a need for oxygen supplementation, and evidence of pulmonary involvement on lung examination and/or chest radiography or computed tomography, were eligible for enrollment. Participants had to have symptom onset and first positive PCR test no more than 14 days before randomization and requirement for oxygen support with a blood oxygen saturation (SpO2) ≥92% (or ≥90% if they had chronic obstructive pulmonary disease) on oxygen ≤6 L/min by facial mask or nasal prongs. Exclusion criteria included high-flow oxygen support, mechanical ventilation or extracorporeal membrane oxygenation (ECMO), evidence of multiorgan failure, prior stay in an intensive care unit (ICU) for the current COVID-19 episode, having received immunoglobulins or any blood products in the past 30 days, or uncontrolled bacterial infection. Standard care according to local practice (antiviral treatment, glucocorticoids, tocilizumab, anticoagulants, and supportive care) was provided.

### Intervention, Randomization, and Blinding

Eligible patients were randomly assigned in a 1:1 ratio to receive a single intravenous infusion of XAV-19 or placebo plus standard care by means of an interactive web-based response system and block randomization. Randomization was stratified according to duration of symptoms (0 to 6 days, 7 to 10 days, or 11 to 14 days) and by center. The saline placebo was administered in the same volumes as the active agents in the XAV-19 group, so that neither the patient nor the investigator could differentiate it from XAV-19 by its appearance.

Based on a previous pharmacokinetic study (phase 2a) of XAV-19 in patients hospitalized with COVID-19 pneumonia, a single infusion of XAV-19 at 2 mg/kg was chosen [15]. Patients and investigators were blinded to the trial's group assignment.

### Outcomes

Patients’ clinical status was assessed on the World Health Organization (WHO) 8-level ordinal scale [16] according to the following categories: (1) discharged with no limitation of activities; (2) discharged with limitation of activities; (3) hospitalization without supplemental oxygen; (4) hospitalization with supplemental oxygen by mask or nasal prongs; (5) ICU or non-ICU hospitalization with noninvasive ventilation (NIV) or high-flow oxygen; (6) ICU hospitalization with intubation and mechanical ventilation (MV); (7) ICU hospitalization with extracorporeal membrane oxygenation or MV and additional organ support (ECMO); and (8) death. The primary outcome was the occurrence of death or respiratory failure through day 15, as defined by a score of ≥5 on the WHO ordinal scale or by an increase of the required O2 supplement (in absolute value) of ≥10 L/min with a nonrebreather mask.

The following secondary outcomes were recorded through day 29: percentage of subjects reporting each severity rating on an 8-point ordinal scale at day 15, primary criterion at days 8 and 29, time to respiratory failure, cumulative incidence of transfer to ICU, time to first day on invasive mechanical ventilation or ECMO (6 or 7 on an ordinal scale), time to weaning off of oxygen support, time to hospital discharge, cumulative incidence of death through days 60 and 29, oxygen-free days, time to National Early Warning Score (NEWS) <2 or hospital discharge, and thrombotic events (defined as any confirmed thrombotic episode including peripheral venous thrombosis, pulmonary embolism, or arterial thrombosis).

### Adverse Events

Safety outcomes included the cumulative incidence of any grade 3 or 4 adverse event (AE) or of any serious adverse event (SAE) and grade changes in the biological and inflammatory patterns of participants over time. The occurrence and severity of AEs were graded according to the Division of AIDS (DAIDS) Table for Grading the Severity of Adult and Paediatric Adverse Events (version 2.1, July 2017) and coded according to the *Medical Dictionary for Regulatory Activities*, version 24.0. The etails of adverse event reporting are described in Supplementary Data 2. An independent data and safety monitoring board reviewed unblinded patient-level data for safety on a regular preplanned basis during the trial.

### Sample Size Calculation

Sample size calculation was based on a previous cohort analysis that reported around 20% progression to severe respiratory failure requiring admission to the ICU among patients hospitalized with oxygen supplementation for COVID-19 pneumonia [1].

We calculated that a sample size of 398 patients would provide the trial with 80% power to detect a between-group difference of 10 percentage points in the incidence of the primary outcome, assuming that 10% of the participants in the XAV-19 group and 20% of those in the placebo group would have an event. The hypothesis of superiority was to be tested at a 2-tailed alpha level of 5%.

### Statistical Analysis

We performed efficacy assessments of the primary and secondary outcomes in the modified intention-to-treat (mITT) population, which included all the patients who had undergone randomization (ITT population) and received full perfusion of XAV-19 or placebo and who had legal requirements (ie, no guardianship or trusteeship, ≥18 years, signed consent) (Supplementary Data 1). The primary outcome was analyzed using a logistic regression model that included a fixed effect for stratification factor (duration of symptom onset at enrollment and center) as a random effect. Missing data were handled by simple imputation by the worst case scenario on the mITT population. If the *P* value of the parameter was ≤.05, then the null hypothesis that there was no difference between groups was rejected. A sensitivity analysis was performed on the ITT population, that is, all randomized patients. Missing data were handled by multiple imputation methods (5 completed data sets were generated in order to pool the results). The multiple imputation model was based on stratification factors (duration of symptom onset at enrollment, center) and the allocated treatment group. This approach assumed that missing data were missing at random. A second sensitivity analysis was performed on the per-protocol (PP) population, that is, randomized patients who had received a full perfusion of XAV-19 or placebo, met the legal requirements, met the primary end point, and did not meet the primary endpoint before treatment, that is, between randomization and perfusion. Prespecified subgroup analyses were performed on the primary end point according to time from symptom onset to enrollment, age, gender, comorbidities, presence of immunodepression, NEWS2 score, ratio of the partial pressure of oxygen to the fraction of inspired oxygen, and COVID-19 serum antibody status at baseline.

For analyses of the secondary outcomes, 95% CIs were not adjusted for multiplicity. Because of the potential for type I error due to multiple comparisons, findings for secondary end points were considered exploratory. Time-to-event analyses (time to respiratory failure or death), when no other risk was in competition, used the frailty model to take into account center as a random effect. All analyses were adjusted for duration of symptoms before enrollment as a fixed effect and censored 29 days after infusion. Time to ICU transfer, to MV or ECMO, to NIV/ high-flow oxygen, to oxygen support weaning, to a NEWS ≤2, and to hospital discharge was compared between groups using the Fine and Gray method to take into account the competing risk of death before the event, adding a frailty factor to include the variability of the randomization done by each center. Oxygen-free days were calculated as the number of days without oxygen between day 1 and day 29. Patients who had died by day 29 were considered to have had no oxygen-free days. This duration was compared between groups using the van Elteren test.

Data were analyzed with R (version 4.1.1) and SAS software (version 9.4, SAS Institute). All tests were 2-tailed, with significance defined as *P* < .05.

### Exploratory Analysis

Exploratory outcome measures were done in a subset of participants and included the decrease of the normalized SARS-CoV-2 viral load in nasopharyngeal (NP) swabs from baseline to day 29; ancillary studies analyzed the postinfusion plasma concentration of XAV-19 at day 1 and the trough level at days 3, 5, 8, 15, and 29, as well as total anti-SARS-CoV-2 S1 protein and inhibiting antibodies in the sera of patients treated with XAV-19 vs placebo. This study was performed in a subgroup of 30 patients included in the core study (16 XAV-19 and 14 placebo). We also included 20 additional patients from a supplementary pharmacokinetic substudy (with a fixed dose of 150 mg XAV-19) and 2 vaccinated patients (after the second dose of COVID-19 Pfizer–BioNTech BNT162b2 vaccine). The main objectives of this substudy were to measure total anti-SARS-Cov-2 S1 protein and inhibiting antibodies in the sera of patients treated with XAV-19, compared with placebo, with postvaccinated patients serving as controls. The kinetics of total anti-SARS-CoV-2 S IgG were assessed by Elecsys anti-SARS-CoV-2 S assay; an electrochemiluminescence immunoassay “ECLIA” was performed on a Cobas e immunoassay analyzer using the Elecsys Anti-SARS-CoV-2 S assay (Roche Diagnotics, Meylan, France) to detect antibodies to SARS-CoV-2 spike protein RBD in patients’ serum (antigens within the reagent capture were predominantly anti-SARS-CoV-2 IgG, but also anti-SARS-CoV-2 IgA and IgM). Spike/ACE-2 interaction blocking antibodies were assessed by testing patients’ sera for their capacity at blocking the binding of recombinant SARS-CoV-2 spike S1 molecule (original strain) to immobilized recombinant human ACE-2 protein (Sino Biological, Eschborn, Germany) [17].

## RESULTS

### Patients

**Figure 1.**
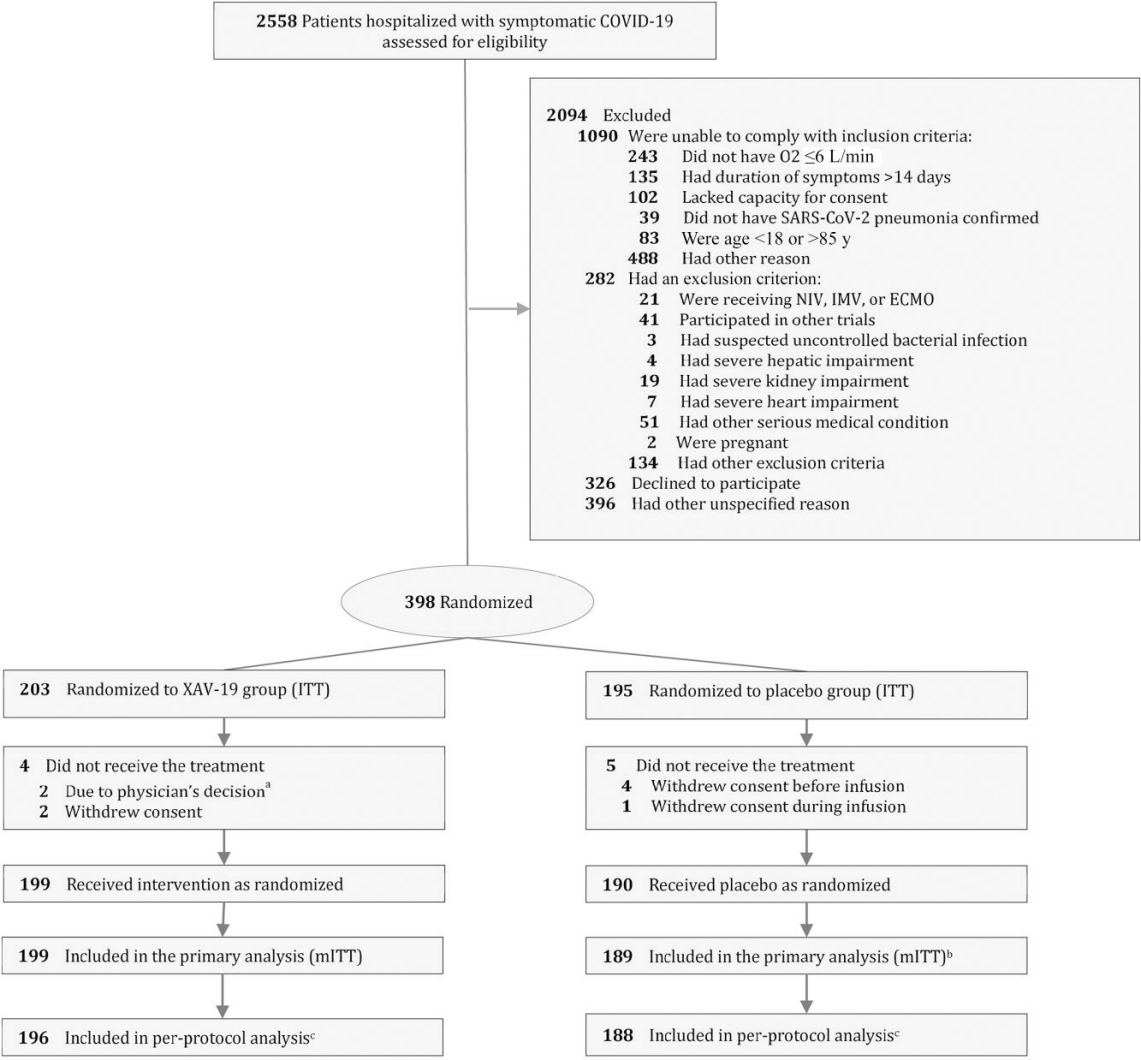
Flow of participants in a study of the effect of a swine glyco-humanized polyclonal neutralizing antibody on survival and respiratory failure in patients hospitalized with COVID-19 pneumonia (POLYCOR trial). ^a^Worsening of respiratory status before infusion. ^b^One patient did not provide a valid written informed consent and was excluded. ^c^The per-protocol analysis excluded 4 patients: 3 patients with respiratory failure before infusion (XAV-19 group n = 2 and placebo group n = 1) and 1 in the XAV-19 group with loss to follow-up between days 1 and 15. Abbreviations: COVID-19, coronavirus disease 2019; ECMO, extracorporeal membrane oxygenation; IMV, invasive mechanical ventilation; ITT, intent to treat; mITT, modified intent to treat; NIV, noninvasive ventilation; O2, oxygen flow; SARS-CoV-2, severe acute respiratory syndrome coronavirus 2; SpO2, blood oxygen saturation.

From January 12, 2021, through April 16, 2021, a total of 2558 hospitalized patients with COVID-19 were assessed for eligibility, and after exclusion, 398 consecutive patients receiving low-flow oxygen therapy from 34 sites in France consented and were enrolled: 203 were randomly assigned to receive XAV-19 and 195 to receive placebo. Of these patients, 199 in the XAV-19 group and 190 in the placebo group received the assigned treatment. In the placebo group, 1 patient did not provide a valid consent and was excluded from the mITT population, which included 388 patients (199 in the XAV-19 group and 189 in the placebo group). Four patients were removed from the per-protocol analysis: 1 patient, in the XAV-19 group, was lost to follow-up after being discharged alive from the hospital, 2 patients in the XAV-19 group and 1 in the placebo group had respiratory failure before infusion (Figure 1). The baseline characteristics of the patients were well balanced in the 2 trial groups. The mean (SD) age of patients was 59.8 (12.4) years, and 249 (64.2%) were men, with a median time (interquartile range [IQR]) of 9 (7–10) days from symptom onset to enrollment. The median duration between hospitalization and assigned treatment administration (IQR) was 1 (1–2) day. Regarding the patient's respiratory illness at baseline, partial pressure of oxygen/fraction of inspired oxygen ratio <300, NEWS score 2–4, and oxygen flow >4L/min at baseline were 80% vs 78%, 13% vs 18%, and 27% vs 23% for the XAV-19 and placebo patients, respectively (Table 1; Supplementary Table 1). Previous medications used by ACEI or ARB2 were in 26.6% vs 17% in the XAV-19 and placebo groups. The proportion of patients infected by the initial SARS-CoV2 virus, the variant Alpha, the variant Beta, the variant Gamma, or other variants was 39.2%, 53.4%, 3.9%, 0.5%, and 3.1%, respectively. Standard care at baseline included glucocorticoids in 93.3% of the patients and remdesivir in 1.0%.

**Table 1.** Demographic and Disease Characteristics of the Patients at Baseline

Characteristics at Baseline^a^	XAV-19 (n = 199)	Placebo (n = 189)
Age, mean (SD), y	59.6 (12.6)	60.0 (12.1)
Distribution, No. (%)		
≤50 y	38 (19.1)	40 (21.2)
50–64 y	101 (50.8)	82 (43.4)
65–74 y	41 (20.6)	50 (26.5)
≥75 y	19 (9.5)	17 (9.0)
Sex, No./total (%)		
Male	127/199 (63.8)	122/189 (64.6)
Female	72/199 (36.2)	67/189 (35.4)
Weight, mean (SD), kg	86.3 (18.9)	86.0 (20.3)
Body mass index, mean (SD), kg/m^2^	29.6 (6.2)	29.6 (6.3)
Body mass index in class, No. (%)		
<30 kg/m^2^	119 (61.3)	115 (61.8)
30–34 kg/m^2^	46 (23.7)	36 (19.4)
35–40 kg/m^2^	20 (10.3)	26 (14.0)
>40 kg/m^2^	9 (4.6)	9 (4.8)
Delay between the 1st symptoms and screening, mean (SD), d	8.5 (2.8)	8.7 (2.7)
Delay between the start of hospitalization and infusion, mean (SD), d	1.9 (3.4)	1.9 (1.6)
Delay between first positive PCR symptoms and infusion, mean (SD), d	3.9 (3.7)	4.2 (3.9)
Delay between 1st symptoms and screening, No. (%)		
≤6 d	46 (23.1)	40 (21.2)
7–10 d	106 (53.3)	101 (53.4)
≥11–14 d	47 (23.6)	48 (25.4)
Illness severity on National Early Warning Score 2, mean (SD)	7.0 (2.3)	6.7 (2.2)
<5	27 (13.6)	35 (18.5)
5–6	56 (28.1)	44 (23.3)
>6	105 (52.8)	101 (53.4)
Ordinal scale for clinical status, No. (%)^b^		
3-Hospitalized, not requiring supplemental oxygen	1 (0.5)	2 (1.1)
4-Hospitalized, requiring low-flow oxygen by mask or nasal prongs	197 (99.0)	186 (98.4)
5-Hospitalized, requiring NIV or use of high-flow oxygen devices	1 (0.5)	1 (0.5)
Type of oxygen supplementation, No. (%)^b^		
Room air	1 (0.5)	2 (1.1)
Low oxygen delivery (nasal cannula)	176 (88.4)	164 (86.8)
Low oxygen delivery (face mask)	19 (9.5)	21 (11.1)
Low oxygen delivery (face mask with reservoir)^c^	2 (1.0)	1 (0.5)
High oxygen delivery (high-flow oxygen, NIV)	1 (0.5)	1 (0.5)
Ratio PaO2/FiO2, mean (SD)^d^	241.4 (82.1)	246.0 (78.6)
Ratio PaO2/FiO2 <300, No. (%)	159 (80.3)	147 (78.6)
Ratio PaO2/FiO2 ≥300, No. (%)	39 (19.7)	40 (21.4)
Oxygen flow at the start of infusion, mean (SD)	3.3 (3.1)	3.4 (3.8)
<4 L/min	145 (72.9)	145 (76.7)
≥4 L/min	54 (27.1)	44 (23.3)
SpO2 at the start of infusion, mean (SD)	94.9 (2.1)	95.3 (1.9)
Laboratory values		
C-reactive protein, mean (SD), mg/L	85.9 (66.7) [n = 193]	80.1 (60.3) [n = 186]
Ferritin, mean (SD), mg/L	1300.2 (1257.2) [n = 156]	1219.2 (1236.7) [n = 162]
No. of comorbidities, No. (%)		
None	51 (26.3)	49 (26.63)
1	72 (37.1)	70 (37.6)
≥2	71 (36.6)	67 (36.0)
Immunosuppressive status, No. (%)^e^	13 (6.5)	14 (7.4)
Negative COVID serology at baseline, No. (%)	56/120 (46.7)	55/124 (44.4)
Previous medications used, No. (%)		
ACEI or ARB2	53 (26.6)	32 (17.0)
Recent use of NSAID	19 (9.8)	15 (8.2)
Anticoagulation	16 (8.0)	12 (6.4)
Corticoids	14 (7.0)	19 (10.1)
Treatments during trial before respiratory degradation, No. (%)^f^		
Remdesivir	2 (1.0)	2 (1.1)
Glucocorticoids	185 (93.0)	177 (93.7)
IL-6 receptor antagonist	9 (4.5)	12 (6.3)

Abbreviations: ACEI, angiotensin-converting enzyme inhibitors; ARB2, angiotensin 2 receptor blocker; COVID, coronavirus disease; FiO2, fraction of inspired oxygen; IL-6, interleukin-6; NIV, noninvasive ventilation; NSAID, nonsteroidal anti-inflammatory drug; PaO2, partial pressure of oxygen; PCR, polymerase chain reaction.

^a^For the evaluation of patients, baseline was defined as the last observation before the administration of XAV-19 or placebo on day 1.

^b^Worst flow of oxygen support was recorded at baseline.

^c^One patient requiring supplemental oxygen by face mask at 6 L/min and 1 patient at 15 L/min (considered respiratory failure before infusion) in the XAV-19 group and 1 patient at 6 L/min in the placebo group.

^d^Worst blood oxygen saturation was recorded at baseline.

^e^Have been considered immunosuppressant on any treatment that interferes with innate or adaptive immunity, such as calcineurin inhibitors, mTOR inhibitors, anti–tumor necrosis factor–α, antilymphocyte antibodies, alkylating agents, or purine base analogues. Long-term corticosteroid therapy was considered immunosuppressive if ≥7 d at a dose ≥1 mg/kg body weight (prednisone equivalent) or for >3 months at a lower dose.

^f^Before the primary end point if it occurred.

### Primary Outcome

Results for the primary and secondary outcomes are shown in Table 2. There was no statistically significant decrease in the cumulative incidence of death or severe respiratory failure through day 15 in the XAV-19 group vs the placebo group (53 [26.6%] vs 48 [25.4%], respectively; absolute risk difference, 0.6%; 95% CI, −6% to 7%; hazard ratio, 1.03; 95% CI, 0.64–1.66; *P* = .90).The ITT sensitivity analysis led to imputing 9 missing values (2%), with similar proportions regarding primary outcome (Table 2). Results for the primary outcome were consistent across subgroups and in all randomized (ITT) and PP populations (Supplementary Table 1, Supplementary Figures 1 and 2).

**Table 2.** Primary and Selected Secondary Outcomes of the POLYCOR Trial for Patients Included in the Primary Analysis (mITT)

Outcome	XAV-19 (n = 199)	Placebo (n = 189)	Adjusted Risk Difference (95% CI)	Odds/Hazard Ratio (95% CI)	*P* Value
Primary outcome, No. (%)					
Occurrence of death or respiratory failure through day 15^a,b^	53 (26.6)	48 (25.4)	0.6 (−6 to 7)	1.03 (0.64–1.66)	.90
Components of the primary outcome, No. (%)					
High-flow oxygen devices (or scale 5)	24 (12.1)	34 (18.0)	…	…	
Invasive mechanical ventilation or ECMO (or scale 6–7)	26 (13.1)	11 (5.8)	…	…	
Death due to any cause (or scale 8)	3 (1.5)	3 (1.6)	…	…	
Selected secondary outcomes					
Primary end point at day 8, No. (%)	51 (25.6)	48 (25.4)	−0.5 (−7 to 6)	0.97 (0.60–1.58)	.92
Primary end point at day 29, No. (%)	53 (26.6)	48 (25.4)	0.6 (−6 to 7)	1.03 (0.64–1.66)	.90
Time to respiratory failure through day 29, median (Q1; Q3), d^c^	3 (2; 4)	3 (2; 4)	0.1 (−6 to 6)	0.99 (0.67–1.48)^d^	.98
Transfer to ICU through day 29, No. (%)	42 (21.1)	38 (20.1)	0.9 (−3 to 5)	1.02 (0.67–1.57)^d^	.91
Invasive mechanical ventilation or ECMO at day 29, No. (%)	26 (13.1)	12 (6.3)	…	…	
Time to invasive ventilation or ECMO, median (Q1; Q3), d^c^	4 (3; 6)	5 (3; 7)	9 (4–13)	2.05 (1.04–4.02)^d^	.037
Time to weaning off of oxygen supplement, median (Q1; Q3), d^c^	8 (7; 8)	7 (7; 8)	−0.4 (7–7)	0.86 (0.70–1.04)^d^	.12
Time to hospital discharge, median (Q1; Q3), d^c^	7 (5; 10)	7 (5; 9)	−4 (−3 to 11)	0.76 (0.62–0.93)^d^	.008
Death through day 60, No. (%)	10 (5.0)	5 (2.6)	2.2 (−0.5 to 5)	1.83 (0.6–5.4)^e^	.55

Abbreviations: ECMO, extracorporeal membrane oxygenation; ICU, intensive care unit; mITT, modified intent to treat.

^a^Progression to high-flow oxygen, noninvasive ventilation, mechanical ventilation, ECMO, or to oxygen support with reservoir mask of ≥10 L/min.

^b^Simple imputation by worst case scenario (1 missing value in XAV-19 group).

^c^Median time before event for patients who had the event.

^d^Survival analysis with competing risk (death before hospital discharge and before day 29) applied using the Fine-Gray regression model.

^e^The model using a frailty model to take into account the variability between centers did not converge. Then no random effects were included in the final model.

### Secondary Outcomes

**Figure 2.**
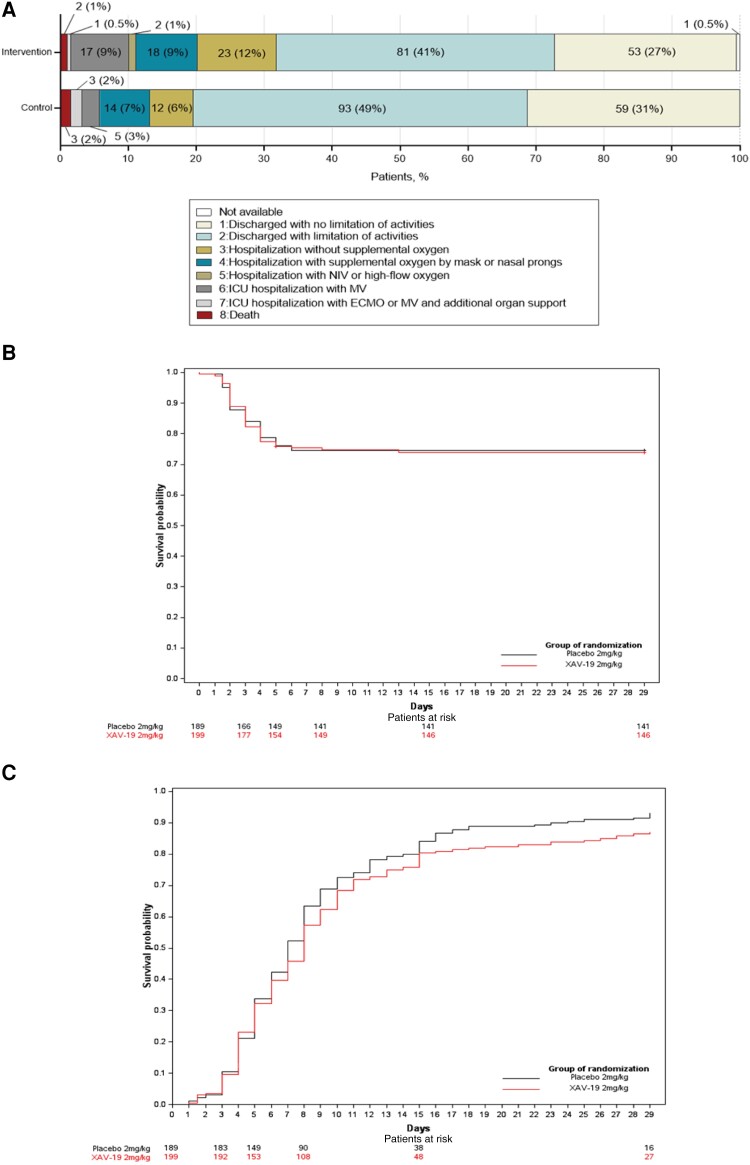
Change in respiratory status on an 8-point scale (A), time to respiratory failure (B), and weaning off of oxygen supplement (C), by group. Abbreviations: ECMO, extracorporeal membrane oxygenation; ICU, intensive care unit; MV, mechanical ventilation; NIV, noninvasive ventilation.

**Figure 3.**
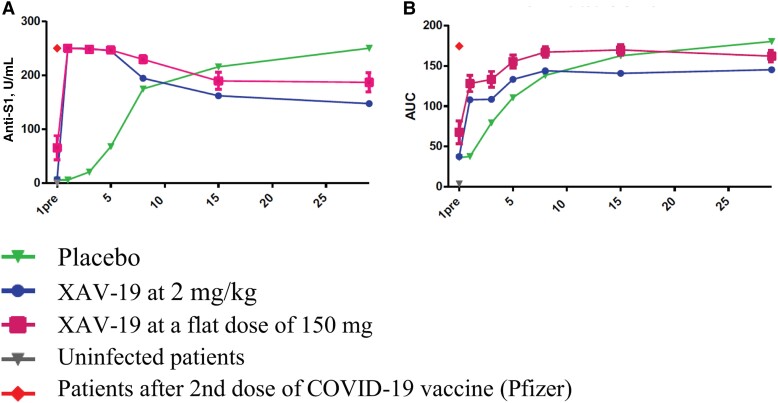
Changes in the total level of anti-SARS-CoV-2 spike S1 proteins (A) and of total inhibitory antibodies (Abs blocking spike S1-ACE2 interactions) (B). Abbreviations: AUC, area under the curve; COVID-19, coronavirus disease 2019; SARS-CoV-2, severe acute respiratory syndrome coronavirus 2.

The distributions of patients’ scores on the 8-level ordinal scale at day 15 and day 29 are shown in Figure 2A and Supplementary Figure 2. Time to respiratory failure, time to weaning off of oxygen, and oxygen-free days were not significantly different between the 2 groups. Through day 29, time to first day under invasive mechanical ventilation or ECMO was 1 day shorter in the XAV-19 group vs the placebo group (median [IQR], 4 [3–6] vs 5 [3–7] days, respectively; adjusted hazard ratio, 2.05; 95% CI, 1.04–4.02; *P* = .037) (Table 2, Figure 2B and C;  Supplementary Table 1). Time to hospital discharge was 1 day longer in the XAV-19 group than the placebo group (median time [IQR], 8 [7–9] vs 7 [7–8] days, respectively; adjusted hazard ratio, 0.76; 95% CI, 0.62–0.93; *P* = .008) (Supplementary Figure 4). By day 29, death from any cause occurred in 3.5% of the patients in the XAV-19 group and 1.6% of the placebo group (adjusted hazard ratio, 2.12; 95% CI, 0.55–8.20) (Table 2; Supplementary Table 1 and Figure 3).

### Exploratory Analyses

A total of 592 NP swabs were analyzed for viral load quantification in 194 participants. There was no significant effect of XAV-19 compared with placebo on the viral kinetics, in either patients with positive or negative COVID-19 serology status at baseline (Supplementary Figure 5 and 6). The postinfusion plasma concentrations of XAV-19 and total anti-SARS-CoV-2 S1 protein and inhibiting antibodies in the sera of patients treated with XAV-19 are presented in Supplementary Table 2. In the imunomonitoring substudy (n = 50 patients), we detected significantly increased levels of neutralizing Ab soon after the injection in patients treated with XAV-19 and in vivo additive antibodies neutralizing effect 2–5 days earlier than in the placebo group, yet these levels remained lower than in the vaccinated controls. Levels of neutralizing Ab then increased to reach a plateau at day 7, likely owing to patient IgG production (Figure 3).

### Safety

In the safety population, adverse events were reported in 75.4% of 199 patients in the XAV-19 group and in 76.3% of 190 patients in the placebo group through day 29 (Table 3); SAEs were reported in 23.1% and 15.8%, respectively, with a higher number of serious infections and pneumonia in the XAV-19 group. No infusion-related events were reported. Fatal events occurred in 7 patients (3.5%) in the XAV-19 group and in 3 (1.6%) in the placebo group through day 29. The most commonly reported cause of death was COVID-19 pneumonia (9/10). Adverse events of interest with respect to XAV-19 were well balanced between the trial groups (Table 3). No patients who received XAV-19 had anaphylaxis or hypersensitivity. A total of 31 adverse events, 14 (7.0%) in the XAV-19 group and 17 (8.9%) in the placebo group, were deemed related to the trial drugs. Of these 31 events, 3 were SAEs: acute delusion in the XAV-19 group, toxic skin eruption and bone marrow failure in the placebo group (Supplementary Table 3 and 4).

**Table 3.** Summary of Adverse Events in the Safety Population of the POLYCOR Trial, According to Randomization Arm From Day 1 (Infusion) to Day 29

Adverse Event^a^	XAV-19 (n = 199)	Placebo (n = 190)
Patients with ≥1 event, No. (%)	152 (76.4)	146 (76.8)
No. of events	593	476
Serious adverse event^b^		
Patients with ≥1 serious adverse event, No. (%)	47 (23.2)	30 (15.8)
No. of serious adverse events	94	57
Death, No. (%)	7 (3.5)	3 (1.5)
Patients with adverse events of interest, No. (%)	73 (36.7)	58 (30.5)
Skin and subcutaneous tissue disorders	12 (6.0)	9 (4.7)
Hepatobiliary disorders	28 (14.1)	27 (14.2)
Nervous system and psychiatric disorders	47 (23.6)	34 (17.9)
Serious infection	17 (8.5)	6 (3.2)
COVID-19 resulting in death	6 (3.0)	3 (1.6)
Pneumonia	10 (5.0)	4 (2.1)
Infusion-related event	0	0
Thrombosis	3 (1.5)	2 (1.0)

Abbreviation: COVID-19, coronavirus disease 2019.

^a^Data were censored at day 29.

^b^Defined as adverse events that result in death, life-threatening, persistent or significant disability, incapacity, prolongation of hospitalization, or other medically important condition as defined by the European Medicines Agency.

## DISCUSSION

In this trial involving hospitalized patients with severe COVID-19 requiring low-flow oxygen supplementation, we found no significant difference in clinical deterioration or mortality between the XAV-19 group and the placebo group at day 15. No safety concern was associated with the use of XAV-19. Adverse events of interest for heterologous antibodies (specifically pyrexia, skin disorders) were similar in the XAV-19 group and in the placebo group, and the vast majority of adverse events were mild or moderate in intensity. The safety of XAV-19 in this study, with no hypersensitivity or allergic reactions, highlights the therapeutic potential of polyclonal glyco-humanized animal-derived antibody technology to treat human diseases.

The absence of observable benefit of XAV-19 in this trial confirms other observations about anti-SARS-CoV-2-neutralizing antibodies in the course of advanced forms of severe COVID-19. In our trial, all patients had pulmonary involvement requiring oxygen support, and 96.9% received glucocorticoids, which may have mitigated the additional benefit of neutralizing antibodies. Indeed, steroids are known to interfere with the effector function of antibodies [18]; their frequent use in our study population, as standard of care for patients with severe COVID-19 on oxygen, could have contributed to limiting the effectiveness of neutralizing polyclonal antibodies. This could also be due to overwhelming infection for which viral blockade by therapeutic antibodies occurs too late to impact outcome. We did not observe any difference between groups following antibody administration in patients whatever the duration of symptoms, although a significant level of antibodies was reached 3 days earlier in XAV-19-treated patients as compared with placebo. Notably, patients with the shorter duration of symptoms had the higher risk of disease progression, as previously described [1, 19–22]. Indeed, time to severe respiratory distress occurred rapidly in both groups, a median of 3 days, suggesting that neutralizing antibodies were infused too late and that progression of the disease course may not be modifiable with antiviral therapy at this stage.

Consistently, SARS-CoV-2-seronegative participants had a similar rate of events in both groups, and no clear effect of XAV-19 on SARS-CoV-2 viral kinetics was observed. However, by using an earlier treatment strategy, XAV-19 had a greater clinical and virological impact [23], as already observed with remdesivir [24] and monoclonal antibodies [25].

The dose of 2 mg/kg of XAV-19 used in the trial was derived from a phase 2a trial, where plasma concentrations >10 times the in vitro neutralization concentration were maintained for at least 8 days [15]. In a model of human ACE-2–expressing mice infected with SARS-CoV-2, a single administration of XAV-19 at the dose of 20 mg/kg (pharmacologically equivalent to 2 mg/kg in humans) was effective to reduce viral load in the lung while the dose of 0.2 mg/kg was not [26]. An alternative hypothesis would be that 2 mg/kg would be a subtherapeutic concentrations, as it is generally estimated that the concentration of antibodies in the endothelial lining of the lungs is 15–30 times lower than in the circulation [27–29]. However, higher or equivalent doses of monoclonal antibodies [23] and neutralizing COVID-19 convalescent plasma [30, 31] have been evaluated in other studies and have not shown effectiveness in reducing mortality when administered late. In addition, no data are available on the effectiveness of nirmatrelvir/ritonavir at such an advanced stage of disease [32]. It is thus most likely that the late stage of COVID-19 evolution in this study is the main reason for the absence of efficacy.

In our study, the number of serious adverse events (SAEs) was higher in the XAV-19 group than in the placebo group, which was associated with more serious infections and pneumonia. The possible higher risk of occurrence of severe respiratory failure requiring mechanical ventilation or ECMO with XAV-19 could be due to several causes, notably the differences in respiratory state or the unbalance of previous medications by ACEI or ARB2 in the XAV-19 and placebo groups. Last, we cannot exclude that infusion of xenoantibodies might have worsened symptoms in the most severely infected COVID-19 patients, favoring respiratory failure by fluid overload or immune-mediated damage. These hypotheses will have to be explored further.

Given its broad in vitro activity against variants and its safety, XAV-19 might provide benefit in prophylactic treatment or when administered in patients for whom there is no immediate risk of rapid clinical deterioration, that is, earlier in the disease course.

### Limitations

First, the main limitation of the study concerns extrapolation of the findings to the current pandemic, given that a majority of the population has already been exposed or vaccinated several times against COVID-19 and given the constant evolution of SARS-CoV-2. Second, viral kinetics were evaluated in less than half of the randomized patients, making it impossible to draw definitive conclusions.

## CONCLUSIONS

Among patients with severe COVID-19 hospitalized for pneumonia requiring low-flow oxygen support, swine glyco-humanized polyclonal neutralizing antibody did not significantly improve the risk of clinical deterioration within 29 days. Further research is needed to determine the efficacy of such antibodies in patients with mild or moderate COVID-19.

## Supplementary Material

ofad525_Supplementary_Data

## Supplementary Data

Supplementary materials are available at *Open Forum Infectious Diseases* online. Consisting of data provided by the authors to benefit the reader, the posted materials are not copyedited and are the sole responsibility of the authors, so questions or comments should be addressed to the corresponding author.

## Notes

***Acknowledgments.*** The POLYCOR trial was supported by the Comité ad hoc de Pilotage National des Essais Thérapeutiques et Autres Recherches sur le COVID-19 (CAPNET, label Priorité Nationale de Recherche—National Research Priority). We thank the Biological Resource Centre for Biobanking (CHU Nantes, Nantes Université, Centre de Resources Biologiques [BB-0033_00040], Nantes, France). We thank the clinical and research teams as well as the pharmacists and virologists at all the participating clinical centers who contributed to the management of the study patients for their commitment to providing optimal patient care.

***Financial support.*** The POLYCOR study was funded and fully supported by the University Hospital of Nantes with support from the Bpifrance (Banque Publique d'Investissement) (BPI France) in the framework of the Investment for the Future Program (Programme d’Investissements d’Avenir) and Xenothera.

***Role of the funder/sponsor.*** Nantes University Hospital Research Department was the sponsor of the study. The funders of the study had no role in study conduct, data collection, or data analysis. Nantes University Hospital, BPI France, and Xenothera provided the trial budget, which covered all trial-related expenses including but not limited to investigator fees, costs related to investigational product supplies, insurance, applicable taxes and fees, and funding to support the activities of the monitoring team.

***Author contributions.*** Drs. Raffi and Gaborit had full access to all of the data in the study and take responsibility for the integrity of the data and the accuracy of the data analysis. Concept and design: Gaborit, Vanhove, Duvaux, Raffi. Acquisition, analysis, or interpretation of data: Gaborit, Lacombe, Guimard, Hocqueloux, Perrier, Dubee, Ferre, Josien, Le Thuaut, Vibet, Jobert, Dailly, Raffi. Drafting of the manuscript: Gaborit, Raffi. Critical revision of the manuscript for important intellectual content: Gaborit, Vanhove, Lacombe, Guimard, Hocqueloux, Perrier, Dubee, Ferre, Josien, Le Thuaut, Vibet, Jobert, Duvaux, Raffi. Statistical analysis: Gaborit, Le Thuaut, Vibet. Obtained funding: Gaborit, Vanhove, Duvaux, Raffi. Administrative, technical, or material support: Gaborit, Perrier, Raffi. Supervision: Gaborit, Perrier, Raffi.

***Group information.*** The POLYCOR study group is presented in Supplementary Data 1.

***Trial registration.*** ClinicalTrials.gov Identifier: NCT04453384.
