# Supplementary material for: Effect of Swine Glyco-humanized Polyclonal Neutralizing Antibody on Survival and Respiratory Failure in Patients Hospitalized With Severe COVID-19: A Randomized, Placebo-Controlled Trial
Source: Open Forum Infect Dis. 2023 Oct 20;10(11):ofad525. doi: 10.1093/ofid/ofad525 (PMC10629360; doi:10.1093/ofid/ofad525)
Supplement: ofad525_Supplementary_Data [file ofad525_supplementary_data.zip › Supplementary 1.docx]

**Supplemental 1 Online Content**

**Effect of Swine glyco-humanized polyclonal neutralizing antibody on survival and respiratory failure in patients hospitalized with COVID-19 pneumonia: A Randomized Clinical Trial**

Benjamin Gaborit, MD, PhD^1,2^, Bernard Vanhove, PhD^3^, Karine Lacombe, MD, PhD ^4^, Thomas Guimard, MD^5^, Laurent Hocqueloux, MD^6^, Ludivine Perrier, PharmD^7^, Vincent Dubee, MD, PhD ^8,9^, Virginie Ferre, MD, PhD ^10^,Celine Bressollette, MD, PhD ^10^, Régis Josien, MD, PhD ^2,11^, Aurélie Le Thuaut, MSc^7,12^, Marie-Anne Vibet, PhD^7,12^, Alexandra Jobert, PhD^7,13^,Eric Dailly, PharmD, PhD^14^, Florence Ader, MD, PhD^15,16^, Sophie Brouard, PhD^2^, Odile Duvaux, MD, PhD^3^, François Raffi, MD, PhD ^1^ for the POLYCOR study group

**List of POLYCOR Investigators and study group**

**Supplement 1.** Trial protocol and statistical analysis plan

This supplemental material has been provided by the authors to give readers additional

information about their work.

**List of POLYCOR Investigators and study group**

**Local Clinical Centre POLYCOR trial staff** (listed in descending order of the number of patients randomised per site)

***1-*** ***Nantes Université, CHU Nantes (1 place Alexis Ricordeau, 44000 Nantes, France, Boulevard Jacques Monod, 44800 Saint-Herblain),***

***Department of Infectious and Tropical Diseases:*** Benjamin GABORIT, MD, PhD (PI); François RAFFI, MD, PhD (Co-I); Maeva LEFEBVRE, MD (Co-I); Charlotte BIRON, MD (Co-I); Raphaël LECOMTE, MD (Co-I); Cécile BRAUDEAU, Marie CHAUVEAU, MD, Eric DAILLY, Colin DESCHANVRES, Matthieu GREGOIRE, Anne-sophie LECOMTE, Laurent FLET, PharmD ; Martine TCHING-SIN PharmD ; Eugenie CLAPEAU, PharmD ; Jérémie ORAIN ; Morgane LE BRAS ; Alexandre DUVAL ; Isabelle PORTIER ; Clara MORA.

***Acute Geriatric Medicine Department*:** Anne-Sophie BOUREAU MD (PI) ; Etienne SERONIE-DOUTRIAUX, MD (Co-I) ; Agnès ROUAUD, MD (Co-I), Pamela HUBLAIN ; Laurence LE JUMEAU DE KERGARADEC ; Carole AGASSE.

***Pulmonology Department:*** Vivien DANIELO, MD (PI), Megguy BERNARD ; Régine VALERO.

***2-Hospital Saint-Antoine AP-HP (184 Rue du Faubourg Saint-Antoine, 75571 Paris, France),***

***Department of Infectious and Tropical Diseases:*** Karine LACOMBE, MD, PhD (PI) ; Diane BOLLENS, MD (Co-I) ; Thibault CHIARABINI, MD (Co-I) ; Nadia VALIN, MD(Co-I) ; Patrick INGILIZ, MD (Co-I) ; Zineb OUAZENE, MD (Co-I) ; Bénédicte LEFEBVRE, MD (Co-I) ; François LECARDONNEL ; Christian TRAN ; Raynald FELIHO ; Manuela LE CAM ; Julie LAMARQUE ; Jean-Luc LAGNEAU ; Cyrielle LETAILLANDIER ; Anne DAGUENEL-NGUYEN, PharmD ; Clémentine MAYALA-KANDA, PharmD , Djeneba FOFANA, MD (Virology Unit), Arianna FIORENTINO, PhD (Biological Resource Center APHP.SU, site Saint Antoine Sorbonne Université).

***3- CHD Vendée La Roche-sur-Yon (Boulevard Stéphane Moreau, 85000 La Roche sur Yon, France),***

***Post-Emergency Medicine and Infectiology Department:*** Thomas GUIMARD, MD (PI) ; Yves BLEHER, MD(Co-I) ; Jean-Luc ESNAULT, MD (Co-I) ; Dominique MERRIEN, MD(Co-I) ; Blandine LE CLAIRE, MD (Co-I) ; Marine MORRIER, MD (Co-I) ; Delphine BOUCHER, MD (Co-I) ; Romain LAMBERET, MD (Co-I) ; Clémentine COUDON, MD (Co-I) ; Romain DECOURS, MD (Co-I) ; Hélène DURAND; Armelle PEGEOT; Edwige MIGNE; Hélène PELERIN, PhD; Yannick POIRIER, PharmD.

***4- CHR Orléans (14, avenue de l’Hôpital, 45100 Orléans, France),***

***Department of Infectious and Tropical Diseases:*** Laurent HOCQUELOUX, MD, PhD(PI) ; Thierry PRAZUCK, MD (Co-I) ; Barbara DE DIEULEVEULT ; Pierre PLOCCO, PharmD ; Jérôme GUINARD, MD (Microbiology Laboratory).

***5- CHU Nice (151 route de St Antoine de Ginestiere, 06202 Nice, France),***

***Department of Infectious and Tropical Diseases:*** Elisa DEMONCHY, MD(Co-I) ; Eric CUA, MD(PI).

***6-Hospital René Dubos (6 Avenue de l’île de France, 95300 Pontoise, France),***

***Intensive and Unscheduled Care Unit – Polypathologies and Systemic Diseases, Infectious and Tropical Pathologies Unit:*** Edouard DEVAUD, MD (PI) ; Stanislas HARENT, MD (Co-I) ; Marion PARISEY, MD (Co-I) ; Céleste LAMBERT, MD (Co-I) ; Elise GOBIN ; Julien MANSON PharmD ; Pierre PASQUIER, PharmD ; Pascale MARTRES (Virology laboratory), Patricia KESSEDJIAN (Virology laboratory).

***7- Hospital Le Mans (194 avenue Rubillard, 72037 Le Mans, France),***

***Department of Infectious and Tropical Diseases*:** Hikombo HITOTO, MD (PI); Nicolas CROCHETTE, MD (Co-I); Lucia PEREZ-GRANDIERE, MD (Co-I); Jean-Baptiste LAINE, MD (Co-I); Arnaud SALMON-ROUSSEAU, MD (Co-I); Guillaume COSSERON, MD (Co-I); Sophie BLANCHI, MD (Co-I).

***8-Hospital de la Croix Rousse, Hospices Civil de Lyon (103 Grande Rue de la Croix Rousse, 69 317 Lyon, France),***

***Department of Infectious and Tropical Diseases*:** Florence ADER, MD, PhD (PI) ; Valérie GALVAN ; Alexia MOULIN ; Corinne BROCHIER ; Julianne ODDONE PharmD ; Maude BOUSCAMBERT-DUCHAMP, MD (Institute of Infectious Agents).

***9-Hospital Robert Debré CHU Reims (Avenue du Général Koenig, 51092 Reims, France),***

***Department of Internal Medicine, Infectious Diseases, and Clinical Immunology:*** Firouzé BANI-SADR, MD, PhD (PI) ; Yohan N’GUYEN, MD (Co-I) ; Maxime HENTZIEN(Co-I) ; Cédric CASTEX ; Philippe BENOIT, PharmD ; Véronique BRODARD (Virologist).

***10-CHU Carémeau (Place du Professeur Robert Debré, 30000 Nîmes, France),***

***Department of Infectious and Tropical Diseases*:** Didier LAUREILLARD, MD (PI) ; Albert SOTTO, MD (Co-I) ; Paul LOUBET, MD (Co-I); Aurélie MARTIN, MD (Co-I); Régine DONCESCO, Julien MAZET; Ian SOULAIROL, PharmD; Robin STEPHAN (Department of Virology).

***11- CHRU Nancy – Hospitals Brabois (Rue du Morvan, 54511 Vandoeuvre Les Nancy, France),***

***Department of Infectious Diseases* :** François GOEHRINGER, MD (PI) ; Nathalie THILLY, PhD ; Michel PREVOT, PharmD, Hélène JEULIN (Virology Unit).

***12-Hospital Cornouaille (14 avenue Yves Thépot, 29000 Quimper, France),***

***Department of Infectious Diseases***: Jean-Philippe TALARMIN MD (PI) ; Lydie KHATCHATOURIAN, MD (Co-I) ; Nadia SAIDAN, MD (Co-I) ; Brice GUERPILLON, MD (Co-I) ; Pascaline RAMEAU ; Nicolas CASSOU, PharmD ; Thomas BRIAND, PharmD ; Florence LE GALL, PharmD (Department of Microbiology); Elodie LE BRETON, MD (Department of Microbiology).

***13-CHU Amiens Picardie (Rond Point Christian Chabrol, 80054 Amiens, France),***

***Department of Infectious and Tropical Diseases***: Cédric JOSEPH, MD (PI); Sandrine SORIOT-THOMAS, MD(Co-I); Claire ANDREJAK, MD, PhD (Co-I); Jean-Philippe LANOIX, MD (Co-I); Sophie BODDAERT, PharmD; Sandrine CASTELAIN, MD, PhD (Virology Laboratory).

***14-Hospital Henri Duffaut (305 rue Raoul Follereau - 84902 Avignon, France),***

***Polyvalent Acute Infectiology Internal Medicine Department:*** Vincent PESTRE, MD (PI); Juliette WOESSNER, MD (Co-I); Sophie BAYLE, MD (Co-I); Stéphanie BRANGER, MD (Co-I); Christine CHRISTIDES, MD (Co-I); Philippe BIELEFELD, MD (Co-I); Adèle LACROIX, MD (Co-I); Roselyne PILLARD-GAGLIANO, MD (Co-I); Isabelle TRINH, PharmD; Pierre LAFITTE, PharmD; Guillermo GIORDANO; Malena FINELLO; Ignacio LEDESMA; Gustavo MARTINI; Benjamin DELAFONTAINE; Iris CORUS; Pierre BABY.

***15- Hospital Foch (40 rue Worth, 92150 Suresnes, France),***

***Pulmonology Department:*** Emilie CATHERINOT, MD (PI); Céline GOYARD, MD (Co-I); Simon CHAUVEAU, MD (Co-I); Jad CHOUCAIR, MD (Co-I); Beatrice D’URSO; Marie DA SILVA COSTA; Lucie LE MEUR, PharmD; Marc VASSE, MD, PhD (Clinical Biology Department); Tiffany, PASCREAU, MD (Clinical Biology Department); Eric FARFOUR, MD (Clinical Biology Department).

***16-GHRMSA Hospital Emile Muller (20 avenue du Dr René Laennec, 68100 Mulhouse, France),***

***Department of Internal Medicine and Clinical Immunology*** : Benjamin DERVIEUX, MD (PI); C Charlotte KAEUFFER, MD (Co-I).

***17-CHU Strasbourg (1 Place de l’Hôpital, 67091 Strasbourg, France),***

***Department of Infectious and Tropical Diseases :*** François DANION, MD (PI); Yves HANSMANN, MD, PhD (Co-I); Nicolas LEFEBVRE, MD (Co-I); Yvon RUCH, MD (Co-I); Axel URSENBACH, MD(Co-I); Catherine SCHMIDT-MUTTER, MD (Co-I); Muhtadi SULIMAN; Anne HUTT, PharmD; Guillaume BECKER, PharmD; Elodie LAUGEL (Virology Laboratory); Sophie BAYER.

***18-CHU Angers (4 rue Larrey, 49933 Angers, France),***

***Department of Infectious and Tropical Diseases*:** Vincent DUBEE, MD, PhD (PI) ; Rafael MAHIEU, MD (Co-I) ; Valérie DANIEL, PharmD ; Caroline LEFEUVRE (Virology Laboratory) ; Alexandra DUCANCELLE (Virology Laboratory).

***19- Private Hospital Antony (1 rue Velpeau - 92160 Antony, France),***

***Internal Medicine Department:*** Jean-Charles GAGNARD, MD (PI) ; Abolfzl MOHEBBI, MD (Co-I) ; Mélanie DEHAIS ; Sophie RACCAH ; Anne-Lise POULIQUEN, PharmD ; Alison KLASEN, PharmD.

***20- Hospital Métropole Savoie (Site de Chambéry, Place Lucien Biset, 73011 Chambéry, France),***

***Department of Infectious Diseases*:** Emmanuel FORESTIER, MD (PI) ; Marie-Christine CARRET, Severine LIARDOT, PharmD ; Jérôme GROSJEAN (Biologist).

***21-CHU Dupuytren Limoges (16 rue Bernard Descottes, 87042 Limoges, France),***

***Department of Infectious and Tropical Diseases:*** Jean-François FAUCHER, MD, PhD (PI); Josselin BRISSET, MD (Co-I); Anne CYPIERRE, MD (Co-I); Hélène DUROX, MD (Co-I); Pauline PINET, MD (Co-I); Sophie DUCROIX-ROUBERTOU, MD (Co-I); Claire GENET, MD (Co-I); Christine VALLEJO, MD, Sébastien HANTZ, MD, PhD (Bacteriology-Virology-Hygiene department).

***22- Hospital Bretagne Atlantique (20 Boulevard Général Maurice Guillaudot, 56017 Vannes, France),***

***Department of Internal Medicine and Infectious Diseases:*** Marie GOUSSEFF, MD (PI) ; Antoine MERLET ; Sébastien BIGOT ; Marion GIRARD DE COURTILLES, PharmD ; Pascal POUEDRAS, MD (Medical biology laboratory), Delphine LARIVIERE, MD.

***23- CHU Caen Côte de Nacre (Avenue Côte-de-Nacre , 14033 Caen, France),***

***Infectiology Department:*** Renaud VERDON, MD, PhD (PI); Sylvie DARGERE, MD (Co-I); Jocelyn MICHON, MD (Co-I); Anna FOURNIER, MD (Co-I); Sylvie BRUCATO; Séverine GAUTIER; Cécile VALENTIN; Anne RICCI; Antoine ALIX PharmD.

***24- Hospital Mont-de-Marsan and Pays des Sources (417 avenue Pierre de Coubertin,***

***40024 Mont-de-Marsan, France),***

***Internal Medicine Department - Infectious Diseases and Rheumatology:*** Flore LACASSIN-BELLER, MD ( PI) ; Sophie ROUSSEAU, MD (Co-I) ; Jérôme DIMET, MD; Anne-Hélène BOIVIN ; Maylis LARREGLE, PharmD ; Guillaume ROUSSEAU (Laboratory department).

.

***25- Hospital Huriez - CHRU Lille (Rue M. Polonovski, 59037 Lille, France),***

***Department of Infectious Diseases:*** Ady ASSAF, MD(Co-I); Fanny VUOTTO, MD (PI); Karine FAURE; Camille JOACHIM, PharmD; Laurence BOCKET (Virology Laboratory).

***26-CHU Réunion (97 avenue du président Mitterrand, 97 448 Saint-Pierre ; Allée des Topazes, 97400 SAINT DENIS, France),***

***Department of Infectious and Tropical Diseases and Internal Medicine* :** Kévin DIALLO, MD (PI) ; Jessy SAFFORE ; Isabelle MADELINE ; Pauline CHABANON, PharmD.

***Pulmonology Department***: Nathalie ALLOU, MD (PI) ; Elisabeth FERNANDES, MD (Co-I) ; Anne-Sophie GRULIERE, PharmD.

***27- Hospital North-CHU Saint Etienne (Avenue Albert Raimond, 42055 Saint-Etienne, France),***

***Infectiology Department:*** Elisabeth BOTELHO-NEVERS, MD (PI) ; Amandine GAGNEUX-BRUNON, MD (Co-I) ; Véronique RONAT ; Nadine CASIMIR, PharmD ; Sylvie PILLET, MD (Infectious Agents Laboratory), Frédérique BERTHOLON, MD (Biological Resource Center).

***28- Hospital Tenon, AP-HP (4 Rue de la Chine, 75970 Paris, France),***

***Department of Infectious Diseases*:** Gilles PIALLOUX, MD, PhD (PI) ; Marwa BACHIR ELRUFAAI, MD (Co-I) ; Ruxandra CALIN, MD (Co-I) ; Pélagie THIBAUT ; Fatima TENDJAOUI, PharmD ; Julie FILLON, PharmD ; Laurence MORAND-JOUBERT (Virologist).

***29- Hospital Côte Basque (13 avenue de l'interne J Loeb, 64100 Bayonne, France),***

***Department of Infectious Diseases*:** Marc-Olivier VAREIL, MD (PI) ; Heidi WILLE, MD (Co-I).

***30- Hospital Sud Francilien (CHSF) (40 Boulevard Serge Dassault 91100 Corbeil Essonnes, France),***

***Pulmonology Department*:** Philippe MENAGER, MD (PI).

***31- Hospital Avicenne AP-HP Bobigny (125 rue de Stalingrad, 93000 Bobigny, France),***

***Department of Infectious and Tropical Diseases*:** Hugues CORDEL, MD (PI) ; Youssouf MOHAMMED-KASSIM ; Vanessa RATHOUIN PharmD ; Ségolène BRICHLER (Microbiology Department).

***32- CHU Martinique (site Fort de France,*** ***97 261 Fort-De-France, France),***

***Department of Infectious, Genetic and Emerging Diseases in the Tropical Zone:*** André CABIE, MD (PI) ; Mélanie LEHOUX, MD (Co-I) ; Karine GUITTEAUD, MD (Co-I) ; Karamba SYLLA, MD (Co-I) ; Bastien BIGEARD, MD (Co-I) ; Valentine CAMPANA, MD ; Isabelle CALMONT, Jean-Louis LAMAIGNERE, PharmD ; Marine DEPPENWEILLER, PharmD ; Christophe PADOIN, PharmD ; Marine THILBAULT, PharmD ; Laurence FAGOUR (Virology) ; Fatiha NAJIOULLAH (Virology) ; Isabelle KOMLA-SOUKHA (Biological Resource Center BRIF BB-0033-00099).

***33- Hospital La Rochelle (Groupe hospitalier littoral atlantique, Rue du Dr Schweitzer, 17009 La Rochelle, France),***

***Department of Infectious Diseases:*** Mariam RONCATO-SABERAN, MD (PI).

***34- Hospitals Civils de Colmar (39 avenue de la Liberté, 68000 Colmar, France),***

***Department of Infectious Diseases*:** Martin MARTINOT, MD (PI); Mahsa MOHSENI ZADEH, MD (Co-I); Simon GRAVIER, MD (Co-I); Ciprian ION, MD (Co-I); Damien KAYSER, MD (Co-I); Anne SCHIEBER PACHART; Magali EYRIEY; Anaïs HENRIC ; Jean DANIEL KAISER, PharmD ; Dominique DE BRIEL, MD (Virologist).

We thank the biological resource centre for biobanking (CHU Nantes, Nantes Université, Centre de ressources biologiques (BB-0033-00040), F-44000 Nantes, France

**Xenothera company:** Odile Duvaux, Gwenaëlle Evanno, Pierre-Joseph Royer, Juliette Rousse, Carine Ciron, Elsa Lhériteau, Gaëtane Rouvray, Alan Mougeolle, Auriane Rillet, Sophie Despons, Edwige Mevel and Françoise Shneiker.

**Center for Research in Transplantation and Translational Immunology, INSERM and Laboratoire d’Immunologie, CIMNA Nantes Université, CHU Nantes:** Régis Josien MD, PhD; Cécile Braudeau PhD, Sophie Brouard, PhD, Hoa le Mai PhD.

**Additional contributions :**

**Sponsor Department, Direction de la Recherche et de l’Innovation, F-44000 Nantes, France:**

**Data Monitoring** : Elise APPAU-DANQUAH, Amélie DANIEL, Virginie GRYBEK, David GUENEAU, Marion GAUTIER, Joëlle MARTIN-GAUTHIER, Emily REBOUILLEAU.

**Data-Managment :** Joseph HERAULT, Tanguy ROMAN

**Clinical Research Assistant** : Sorady PLANTARD.

**Project Managment back-up** : Patrice CHAUVEAU

**Adverse Event Monitoring** : Anne CHIFFOLEAU, MD.

**Adverse Event Monitoring Assistant**  : Stéphanie THAUVIN.

**Pharmacy Clinical Trials Unit** : Laurent FLET, PharmD ; Martine TCHING-SIN, PharmD ; Eugenie CLAPEAU, PharmD.

**Data Safety Monitoring Committee (this committee provided independent medical expertise on the occurrence of adverse events and whether the trial needed to be stopped on grounds of adverse events) :**

Isabelle CHARREAU *(INSERM SC10-US19 "Essais Thérapeutiques et Maladies Infectieuses"*

*Hôpital Paul Brousse, 16 Avenue Paul Vaillant Couturier, 94807 Villejuif Cedex)*

Bruno HOEN, MD, PhD (Infectious Diseases Department, University of Besançon, France);

Caroline SOLAS-CHESNEAU, MD, PhD *(Laboratoire de Toxicologie, Faculté de Pharmacie 27 Bd Jean Moulin, 13005 Marseille ; Laboratoire de Pharmacocinétique et de Toxicologie, CHU La Timone, AP-HM, 264 rue Saint-Pierre, 13005 Marseille)*

Astrid VABRET, MD, PhD *(Laboratoire de Virologie, CHU de Caen, Avenue Georges Clémenceau, 14033 Caen Cedex 9 ; Groupe de Recherche sur l’Adaptation Microbienne (GRAM 2.0) Normandie Univ, UNICAEN, UNIROUEN, GRAM2.0, 14000 CAEN, France)*

**Supplement 1.** Trial protocol (v7.0 dated 31/05/2021) and statistical analysis plan

**Protocol POLYCOR**

**Eudract:** No. 2020-002574-27

**Ref:** RC20_0230

**"A randomized, double-blind, placebo-controlled phase 2 (2a and 2b) study to evaluate the safety and efficacy of XAV-19 in patients with COVID-19 induced moderate pneumonia"**

**Coordinating Investigator:**

Dr Benjamin GABORIT

Infectious Diseases Department, Nantes University Hospital

44093 Nantes Cedex 1 - France

Phone : +33 (0)2 44 76 82 92 - Fax : +33 240 083 181

Email: benjamin.GABORIT@chu-nantes.fr

**Methodology expert:**

Marie-Anne VIBET et Aurélie Le THUAUT

Plateforme de Méthodologie et Biostatistique

CHU de Nantes

5 allée de l’Ile Gloriette

44093 NANTES cedex1

Phone : +33 2 53 48 28 45 et +33 2.44.76.68.47

Email : marieanne.vibet@chu-nantes.fr et Aurelie.LETHUAUT@chu-nantes.fr

**Sponsor:**

**
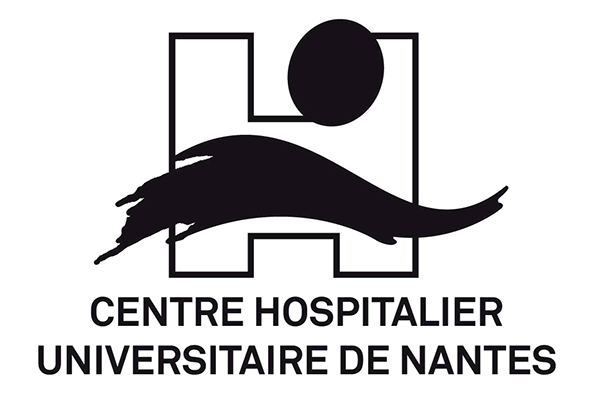
Nantes University Hospital**Medical Affairs

and Research Department

5, allée de l’île Gloriette
44 093 Nantes cedex 01 (FRANCE)

Tel: 33 (0)2 53 48 28 35
Fax : 33 (0)2 53 48 28 36

**INVESTIGATOR'S SIGNATURE**

# *LIST OF ABBREVIATIONS*

| Ab(s)  ADE | Antibody(ies)  Antibody-Dependent Enhancement |
| --- | --- |
| ANSM | Agence Nationale de Sécurité du Médicament et des produits de santé |
| ARDS | Severe Acute Respiratory Distress |
| CNIL | Commission Nationale de l’Informatique et des Libertés |
| CRF | Case Report Form |
| DSMC | Data and Safety Monitoring Committee |
| CRA | Clinical Research Associate (monitor) |
| eCRF | Electronic Case Report Form |
| ECG | Electrocardiogramm |
| EMA | European Medicines Agency |
| ERB | Ethical Review Board (CPP in France) |
| FIH | First-in-Human = Essai de 1^ère^ administration chez l'homme |
| GCP | Good Clinical Practice |
| IB | Investigator Brochure |
| IgG | Immunoglobulin G |
| IgM  IMP | Immunoglobulin M  Investigational Medicinal Product |
| IMV | Invasive Mechanical Ventilation |
| MNP | MonoNuclear Phagocyte |
| PD | Pharmacodynamic |
| PK | Pharmacokinetic |
| PUI | Hospital pharmacy (Pharmacie à usage intérieur) |
| SAE | Serious Adverse Event |
| SAR | Serious Adverse Reaction |
| RM | Reference Methodology |
| RSI | Reference Safety Information |
| RT-PCR | Reverse-Transcriptase Polymerase Chain Reaction |
| SmPC | Summary of Product Characteristics |
| SUSAR | Suspected Unexpected Serious Adverse Reaction |
|  |  |

# *Contents*

[*LIST OF ABBREVIATIONS* 3](#_Toc68687300)

[*Contents* 5](#_Toc68687301)

[INTRODUCTION 8](#_Toc68687302)

[1. Justification of the study 9](#_Toc68687303)

[1.1. Positioning of the study 9](#_Toc68687304)

[1.2. Benefits and risks for subjects taking part in the study 11](#_Toc68687305)

[1.2.1. Benefits 11](#_Toc68687306)

[1.2.2. Risks 12](#_Toc68687307)

[1.2.3. Benefit / risk balance 14](#_Toc68687308)

[1.3. Description and justification of the treatment plan 15](#_Toc68687309)

[2. Objectives and endpoints of Phase 2a 20](#_Toc68687310)

[2.1. Primary objective and endpoint 20](#_Toc68687311)

[2.1.1. Primary objective 20](#_Toc68687312)

[2.1.2. Primary endpoint 20](#_Toc68687313)

[2.2. Secondary objectives and endpoints 20](#_Toc68687314)

[2.2.1. Secondary objectives 20](#_Toc68687315)

[2.2.2. Secondary endpoint(s) 21](#_Toc68687316)

[2.3. Objective and endpoints for exploratory studies for phase 2a 22](#_Toc68687317)

[3. Objectives and endpoints of Phase 2b 24](#_Toc68687318)

[3.1. Primary objective and endpoint 24](#_Toc68687319)

[3.1.1. Primary objective 24](#_Toc68687320)

[3.1.2. Primary endpoint 24](#_Toc68687321)

[3.1. Secondary objectives and endpoints 24](#_Toc68687322)

[3.1.1. Secondary objectives 24](#_Toc68687323)

[3.1.2. Secondary endpoint(s) 26](#_Toc68687324)

[3.2. Objective and endpoints for ancillary studies for phase 2b 27](#_Toc68687325)

[3.2.1. Pharmacokinetic Study (90 patients) 27](#_Toc68687326)

[3.2.2. Immunomonitoring Study (100 patients) 28](#_Toc68687327)

[3.2.3. Terminal ancillary Study (20 additional patients) 28](#_Toc68687328)

[4. Study treatment 30](#_Toc68687329)

[4.1. Description and mode of administration 30](#_Toc68687330)

[4.1.1. Product Characteristics 30](#_Toc68687331)

[4.1.2. Administration 31](#_Toc68687332)

[4.1.3. Other study treatments 32](#_Toc68687333)

[4.2. Treatment compliance follow-up 32](#_Toc68687334)

[4.3. Experimental drug circuit 33](#_Toc68687335)

[4.3.1. General circuit 33](#_Toc68687336)

[4.3.2. Experimental drug storage conditions 33](#_Toc68687337)

[4.3.3. Unblinding procedure 34](#_Toc68687338)

[4.4. Authorised and unauthorised treatments 34](#_Toc68687339)

[4.4.1. Authorised treatments 34](#_Toc68687340)

[4.4.2. Unauthorised treatments 35](#_Toc68687341)

[5. Study population 36](#_Toc68687342)

[5.1. Description of the population in Phase 2a 36](#_Toc68687343)

[5.1.1. Inclusion criteria 36](#_Toc68687344)

[5.1.2. Exclusion criteria 37](#_Toc68687345)

[5.2. Description of the population in Phase 2b 38](#_Toc68687346)

[5.2.1. Inclusion criteria 38](#_Toc68687347)

[5.2.2. Exclusion criteria 39](#_Toc68687348)

[6. Study design and conduct 40](#_Toc68687349)

[6.1. Study schedule for phase 2A 40](#_Toc68687350)

[6.1.1. Screening Visit (Day –2 / Day 1) 40](#_Toc68687351)

[6.1.2. Inclusion Visit (Day 1) 41](#_Toc68687352)

[6.1.3. Visits on Day 2, Day 4, Day 6, Day 7, Day 11 and Day 13 42](#_Toc68687353)

[6.1.4. Visit on Day 3 42](#_Toc68687354)

[6.1.5. Visit on Day 5 43](#_Toc68687355)

[6.1.6. Visits on Day 8, Day 15 44](#_Toc68687356)

[6.1.7. Visit on Day 29 / End of Study Visit 44](#_Toc68687357)

[6.1.8. Follow-up phone call at Day 60 (M2) 45](#_Toc68687358)

[6.1.9. Early Withdrawal Visit 45](#_Toc68687359)

[6.2. Study schedule for phase 2B 50](#_Toc68687360)

[6.2.1. Screening Visit (Day –2 / Day 1) 50](#_Toc68687361)

[6.2.2. Inclusion Visit (Day 1) 51](#_Toc68687362)

[6.2.3. Visit on Day 2 52](#_Toc68687363)

[6.2.4. Visit on Day 3 53](#_Toc68687364)

[6.2.5. Visit on Day 5 (if still hospitalized) 53](#_Toc68687365)

[6.2.6. Visits on Day 8, Day 15 54](#_Toc68687366)

[6.2.7. Visit on Day 29 / End of Study Visit 54](#_Toc68687367)

[6.2.8. Follow-up phone call at Day 60 (M2) 55](#_Toc68687368)

[6.2.9. Early Withdrawal Visit 55](#_Toc68687369)

[6.3. Procedures details 59](#_Toc68687370)

[6.4. General study methodology 66](#_Toc68687371)

[6.5. Study diagram 66](#_Toc68687372)

[6.5.1. Phase 2a – Group1 66](#_Toc68687373)

[67](#_Toc68687374)

[67](#_Toc68687375)

[6.5.2. Phase 2a - Group2 68](#_Toc68687376)

[68](#_Toc68687377)

[6.5.3. Phase 2b 70](#_Toc68687378)

[6.6. Identification of all data sources not included in the medical record 71](#_Toc68687379)

[6.7. Rules for discontinuing subject participation 71](#_Toc68687380)

[6.7.1. Criteria in respect of early withdrawal of a subject from the study 71](#_Toc68687381)

[6.7.2. Procedures in respect of early withdrawal of a subject from the study 72](#_Toc68687382)

[6.7.3. Criteria in respect of discontinuation of all or part of the study (excluding biostatistical considerations) 73](#_Toc68687383)

[6.8. Patient medical care at the end of the study 74](#_Toc68687384)

[7. Data Management AND STATISTICS 75](#_Toc68687385)

[7.1. Data entry and data collection 75](#_Toc68687386)

[7.1.1. Data entry, processing and circulation 75](#_Toc68687387)

[7.1.2. Patient identification 75](#_Toc68687388)

[7.1.3. Encoding data 76](#_Toc68687389)

[7.1.4. Encoding data 76](#_Toc68687390)

[7.2. Statistics 77](#_Toc68687391)

[7.2.1. Description of planned statistical methods, including planned intermediate analysis schedule 77](#_Toc68687392)

[7.2.2. Statistical justification of the number of inclusions 78](#_Toc68687393)

[7.2.3. Expected level of statistical significance 79](#_Toc68687394)

[7.2.4. Consideration method for missing, unused or invalid data 79](#_Toc68687395)

[7.2.5. Management of changes made to the initial analytical strategy 80](#_Toc68687396)

[7.2.6. Choice of subjects to be included in analysis 81](#_Toc68687397)

[7.2.7. Randomisation 81](#_Toc68687398)

[8. Pharmacovigilance and adverse event management 83](#_Toc68687399)

[8.1. Definitions 83](#_Toc68687400)

[8.2. Safety evaluation parameters 85](#_Toc68687401)

[8.2.1. Specific safety-related evaluation criteria 85](#_Toc68687402)

[8.2.2. Methods and schedule envisaged to measure, compile and analyse safety evaluation parameters 85](#_Toc68687403)

[8.3. List of expected ARs 86](#_Toc68687404)

[8.4. Adverse event management 86](#_Toc68687405)

[8.4.1. AR/AE collection 86](#_Toc68687406)

[8.4.2. Management of possible adverse reaction to infusion of XAV-19 86](#_Toc68687407)

[8.4.3. SAR/SAE reporting 87](#_Toc68687408)

[8.4.4. Reporting period 87](#_Toc68687409)

[8.4.5. Data and Safety Monitoring Committee (DSMC) 87](#_Toc68687410)

[8.5. Follow-up procedure and period for subjects following the onset of adverse events 88](#_Toc68687411)

[8.5.1. Procedure to follow for the patient concerned by the SAE 88](#_Toc68687412)

[9. Administrative and regulatory aspects 89](#_Toc68687413)

[9.1. Source data and document access rights 89](#_Toc68687414)

[9.2. Trial monitoring 89](#_Toc68687415)

[9.3. Scientific Committee 89](#_Toc68687416)

[9.3.1. Composition 89](#_Toc68687417)

[9.3.2. Frequency of meetings 90](#_Toc68687418)

[9.3.3. Role 90](#_Toc68687419)

[9.4. Inspection / Audit 91](#_Toc68687420)

[9.5. Ethical considerations 91](#_Toc68687421)

[9.5.1. Written informed consent 91](#_Toc68687422)

[9.5.2. Ethical Review Board 91](#_Toc68687423)

[9.6. Registration with the competent authorities 91](#_Toc68687424)

[9.7. Amendments to the protocol 92](#_Toc68687425)

[9.8. Study funding and insurance 92](#_Toc68687426)

[9.9. Publication rules 92](#_Toc68687427)

[9.10. Outcome of biological samples 93](#_Toc68687428)

[9.11. Source data archiving 93](#_Toc68687429)

# INTRODUCTION

Early inhibition of entry and replication of the severe acute respiratory syndrome coronavirus 2 (SARS-CoV-2) is a very promising therapeutic approach. Polyclonal neutralizing antibodies offers many advantages such as providing immediate immunity, consequently blunt an early pro-inflammatory pathogenic endogenous antibody response and lack of drug-drug interactions^1–3^.

Because a suboptimal endogenous early antibody response with regard to SARS-CoV-2 replication in severe cases is observed, neutralising antibody treatment can be very interesting for patient with COVID-19 induced moderate pneumonia^4,5^. Convalescent plasma to treat infected patients is therefore an interesting therapeutic option currently under evaluation (COVIPLASM NCT04324047). However, the difficulties of collecting plasma and its safety aspects are not adapted to many patients.

A new polyclonal humanized anti-SARS-CoV2 antibodies (XAV-19) is being developed by Xenothera, which can be administered as intravenous treatment. XAV-19 is a heterologous swine glyco-humanized polyclonal antibody (GH-pAb) raised against the spike protein of SARS-CoV-2, inhibiting infection of ACE-2 positive human cells with SARS-CoV-2. Pharmacokinetic and pharmacodynamic studies have been performed in preclinical models including primates and a First In Human study with another fully representative GH-pAb from Xenothera is ongoing in volunteer patients recipients of a kidney graft. These studies indicated that 5 consecutive administrations of GH-pAbs can be safely performed in humans.

The objective of this 2-steps phase 2 randomized double-blind, placebo-controlled study is 1) to define the optimal and safety XAV-19 dose to administrate in patients with COVID-19 induced moderate pneumonia ; 2) to show the clinical benefit of selected dose of XAV-19 when administered to patients with COVID-19 induced moderate pneumonia.

# Justification of the study

## Positioning of the study

In December 2019, Coronavirus disease 2019 (COVID-19) emerged in Wuhan, China. After 12 months COVID-19 causes more than 75 034 000 confirmed cases and more than 1 663 000 deaths (375 000 in Europe).

In the lack of effective therapy and without any control of the virus’ spread, the World Health Organization declared a public health emergency of international concern on 30 January 2020.

Severe acute respiratory syndrome coronavirus 2 (SARS-CoV-2), is a protein-enveloped RNA virus^6^ that induces influenza-like symptoms (fever, cough, dyspnea, vomiting, diarrhea) requiring admission to hospital in 20% of cases for respiratory illnesses and to intensive care unit in 5% of cases for severe diseases^7^. Over 25% of patients develop acute respiratory distress during the second week of disease with the onset of severe ARDS^8^.

SARS-CoV-2 infection has been reported to induce both direct organ damage^9,10^ and inappropriate immune response causing "viral sepsis"^11–13^. The pathophysiological mechanisms of severe infection are poorly understood^14^. In the most critical cases, the overwhelming immunological reactions induced by systemic multiorgan viral invasion lead to multiorgan failure and subsequent mortality^15^. It is likely that both antivirals and blockage of inflammatory pathways are needed to optimize responses.

Due to the urgent need to develop available therapies to manage the pulmonary complications of COVID-19, many efforts continue to develop antivirals, and immunotherapies against COVID-19. Presently, none have yet demonstrated significant efficacy.

By providing immediate immunity and inhibiting entry into cells, convalescent plasma has been demonstrated to improve survival rate of patients with SARS-CoV infections in 2003^16^. SARS-CoV-2 has been shown to use the same cell entry receptor as SARS-CoV, angiotensin-converting enzyme 2 (ACE2)^17,18^. With the inhibition of SARS-CoV-2 spike glycoprotein (S) - mediated entry into cells, convalescent plasma is a promising approach to treat patients infected with SARS-CoV-2. Early administration of neutralizing Abs may inhibit viral entry and replication and consequently blunt an early pro-inflammatory response.

Xenothera has developed a novel polyclonal glyco-humanized (GH-pAb) anti-SARS-CoV2 swine antibody named “XAV-19” (see Vanhove et al. 2020). GH-pAbs^19,19^ (described in Rousse et al and Salama et al) have been evaluated for safety, pharmacokinetics and pharmacodynamics effects in preclinical assessments in non-human primates and in volunteer kidney recipient patients dosed daily for 5 days at the three doses levels of 0.6 mg/kg, 1 mg/kg, 3 mg/kg, 6 mg/kg, 8 mg/kg (Rousse J. Oral communication BOS29 at the 19th Congress of the European Society for Organ Transplantation, September 15-18, Copenhagen, Denmark). 40 mg/kg is therefore the maximal cumulative dose that has been administered in human. While the study is still ongoing, the DSMC agreed for opening of the second cohort of the LIS1 protocol which will include 5 patients at therapeutic dose of 5 times at 8 mg/Kg (cumulative dose of 40 mg/Kg).

Efficacy of neutralizing Abs

In mice, neutralizing Ab against SARS-CoV elicited by primary infection can protect from secondary-infection and prevent SARS-Cov replication in respiratory tract of naïve mice^16^.

In patients, small retrospective case-comparison studies in patients with SARS-CoV-2, suggested a case fatality rate reduction after convalescent plasma treatment^20,21^. An early administration, at a time where pathology may be driven mainly by viral replication appears most suitable.

Safety Profile

The issue of the potential toxicity associated with convalescent plasma and the difficulty in collecting it is a major limitation to the widespread use of this treatment. XAV-19 is a novel GH-pAb from Xenothera technology. Per se, XAV19 has now been administered at 0,5mg/kg twice in 8 patients, and at 2mg/kg in 6 patients (1/6 with 2 doses, the other 5 patients with single dose) of POLYCOR phase 2a trial with satisfactory safety profile assessed at 8 days following injection. Moreover, it is chemically comparable to LIS1, a lymphodepleting GH-pAb introduced in the clinic by Xenothera in 2019. LIS1 has been safely administered to kidney graft recipients at cumulated doses up to 40 mg/Kg, representing 80-fold the lowest dose foreseen for XAV-19 in the POLYCOR study.

New polyclonal glyco-humanized anti-SARS-CoV2 antibodies formulation

The XAV-19 drug substance (DS) is a liquid, colourless, sterile solution concentrated at 5.0 +/- 0.5 mg/ml. XAV-19 is generated as follows: the active substance manufacturing process starts from a pool of swine serum collected after immunisation with a recombinant antigen, (SARS-Cov-2 spike protein domain). It includes a primary clarification step by precipitation followed by three chromatographic steps (one capture and two polishing steps), viral nanofiltration, ultrafiltration and diafiltration against formulation buffer pH8.0. The purified bulk is complemented with Tween 80 (0.05%), filtered through a 0.22 μm filter and vialed into 6R vials containing 5 ml (extractible volume), representing 25 mg of product. Vials are stored at 5 ± 3°C. The stability for the vials used in Phase 2a is 6 months and 10 months for the vials used until April 2021 in Phase 2b. The shelf life will be extended in May 2021 to 18 months.

POLYCOR study

Knowing GH-pAbs are well tolerated in human and that neutralizing Abs can be an effective therapeutic approach during SARS disease, the POLYCOR aims to address, in Randomized double-blind, placebo-controlled study the efficacy of polyclonal humanized anti-SARS-CoV2 antibodies (XAV-19) in patients with COVID-19 induced moderate pneumonia.

Note, at the end of phase 2b, an ancillary study on 20 patients will be performed. In this open-label additional sub-study, these 20 patients will receive a fixed dose of 150 mg of XAV-19.

## Benefits and risks for subjects taking part in the study

### Benefits

#### Individual benefit

Although most patients infected with SARS-CoV-2 have a mild illness, over 25% of hospitalised patients for COVID19 disease develop acute respiratory distress during the second week of hospitalization with the onset of severe ARDS.

Polyclonal humanized anti-SARS-CoV2 has the potential to significantly decrease the severe complications induced by SARS-CoV-2 in patients by effectively freezing the disease process. Other benefits are to blunt the pneumopathy-induced damage and other COVID-19-associated injuries such as acute kidney injury (AKI), myocarditis, secondary bacterial infections.

This treatment is expected to shorten the duration of requirement for oxygenotherapy and of hospital stay with minimization of physical, psychological and economic complications related with prolonged stay. The ultimate clinical benefit of this therapeutic approach is globally to prevent death.

#### Collective benefit

This study will determine the clinical benefit of XAV-19 when administered to patients with COVID-19 induced moderate pneumonia. The first step of the study is expected to define the dose and safety to use XAV-19. Preliminary PK data of phase 2a shows that the concentration at day 5 after one single dose of 0,5 mg/Kg at day 1 reaches level around 5 ug/ml in peripheral blood and reaches 30 ug/ml after a single dose of 2 mg/Kg, a level. With a single dose of 2mg/kg (n=4 patients) preliminary analysis show plasma level above 10 µg/ml at least 8 days that might confers a potential clinical benefit.

Such treatment might become a new therapeutic option to provide COVID-19-infected patients an effective treatment (in combination with anti-viral and immunotherapies). Further studies could later evaluate such passive immunotherapy as a potential post-exposure prophylaxis.

### Risks

#### Individual risk

- Physical risks and constraints

For the patients included, an additional visit is scheduled at screening.

Realization of all the acts are representing the usual medical practice except the additional blood samples that will be collected specifically for the research (PK analysis, Neutralizing antibody titers, Cytokines analysis, Lymphocyte sub-populations and transcriptomic analyses), the RT-PCR SARS-Cov2 (Nasopharyngeal swab samples), and the Intravenous infusions of XAV-19 or placebo.

- Disease-related risks

Severe acute respiratory syndrome coronavirus 2 (SARS-CoV-2), induces influenza-like symptoms (fever, cough, dyspnea, vomiting, diarrhea) requiring admission to hospital in 20% of cases for respiratory illnesses and to intensive care unit in 5% of cases for severe diseases (3). Over 25% of patients develop acute respiratory distress during the second week of hospitalization with the onset of severe ARDS (4). After 12 months COVID-19 causes more than 75 034 000 confirmed cases and more than   1 663 000deaths ( 367 000 in Europe).

- Test treatment risks including placebo (adverse reactions)

***Potential adverse drug reaction and recommended monitoring***

XAV-19 has not been evaluated in many patients; therefore, few data exist on the possible reaction to single or multiple doses of XAV-19 in adult men or women. XAV-19, as a polyclonal IgG with anti-COVID-19 activity, is intended to bind to and neutralize entry of COVID-19 viral particles into ACE-2-positive human cells. It has been obtained by immunization of swines with a recombinant protein expressed in HEK293 cells, corresponding to the RBD domain of the S1 moiety of the Spike molecule of SARS-CoV-2. It should present a narrower antigen target array than the IgG fraction of Covid-19 convalescent plasma.

The pre-clinical efficacy studies detailed in the XAV-19 Investigation Brochure suggest XAV-19 will have strong neutralizing activity. The risks associated with viral neutralization with other antibodies such as convalescent plasma should apply.

Although no information is available yet on possible reactions to XAV-19, information is available on possible serious reactions related to treatment with other products from Xenothera where no adverse effect has been observed yet, and to treatments with other animal-derived globulins such as Thymoglobulin® (information available in Thymoglobulin HIGHLIGHTS OF PRESCRIBING INFORMATION). Described reactions to animal globulin infusion comprised tolerance-related events such as serum sickness, raising an itchy rash, difficulty of breathing, stomach pain, swelling of the face, tongue or throat, feeling or being sick, dizzy or feeling faint, tiredness, joint pain, headache, bleeding or bruising more than normal, irregular or fast heartbeat, symptoms of infection such as fever, chills, sore throat, mouth ulcers. These reactions are typically related to sensibilization events (allergy, serum sickness type hypersensitivity) and the special design of XAV-19, in which IgG are devoid of Neu5Gc and α-galactose residues, should greatly reduce their occurrence.

***Infusion-related reactions***

Infusion-related reactions may occur with antibody-related products and has been reported with other agents containing animal immunoglobulins. Pre-clinical investigations with another GH-pAb, LIS1, in macaques, also revealed possibility of chronic thrombosis at the infusion sites, with an increased incidence/severity of intimal thickening of the veins when compared with infusion of control item. Mononuclear inflammatory infiltrates and subcutaneous inflammation was also noted.

Hypersensitivity reactions can manifest as fever, chills, urticaria, dyspnea, headaches, myalgia, and/or hypotension. A serious infusion reaction that results in anaphylaxis is a rare event in therapy with a product containing animal immunoglobulins. If a severe hypersensitivity reaction occurs, XAV-19 should be discontinued, and appropriate therapy should be initiated.

***Carcinogenicity, mutagenicity and fertility***

No direct risks of carcinogenicity, mutagenicity or fertility are typically associated with antibodies (refer to Investigator Brochure).

1. Reactogenicity during and following IV administration
2. anaphylactic reaction
3. Occurrence of serum sickness disease between Day 1 and D28: hypotension (TAS < 40 mm Hg from baseline), emergent fever, rash, polyarthralgias, lymphadenopathy, proteinuria, renal insufficiency
4. Occurrence of positive immune complexes
5. Hypocomplementemia (low C3 level in serum)

Only local adverse events with pain, erythema, irritation are expected for placebo; the amount of NaCl does not suggest systemic hydro electrolytic or blood pressure adverse effects, nor infection.

### Benefit / risk balance

The study drug would demonstrate an efficacy and a good tolerability compared to previous treatment with a direct benefit for infected patients and the community. However, this cannot be guaranteed. It is also possible that patients may receive no benefit from being in this study. Preliminary studies of another GH-pAb in volunteer recipients of kidney grafts did not report any evidence of poor tolerance.

**1.2.4. Risk management**

ANSM has given their scientific recommendation to conduct the first part of the study (phase 2a) as a first-in-human study, with sequential enrolment to check for safety.

After enrollment of the first two patients in the low dose Group (one patient treated and one patient placebo), data will be obtained after first (Day 1) and second (Day 5) infusions to collect any severe adverse events that might have occurred, especially in the first 48 hours following each infusion.

DSMC will have to review immediately safety data of the first 2 patients (based on Day 8 information) and provide advice on continuing enrolment of the 6 last patients of Group 1 (low dose). DSMC statement, and sponsor decision with regards to study continuation will be immediately communicated to ANSM.

If there is no signal of severe intolerance by D8 of enrollment of the first two patients, the rest of the group will be enrolled with the same process of review of tolerance.

When the final patient of Group 1 has been enrolled, Day 8 safety data of all 8 patients from this low-dose cohort will have to be reviewed by the DSMC to advise of starting high dose group (Group 2). DSMC statement, and sponsor decision with regards to study continuation will be immediately communicated to ANSM.

At any time during the phase 2a study, if a serious adverse event occurs, this will be communicated to the DSMC, and the sponsor and coordinating investigator will have the responsibility to decide whether DSMC must meet to advise on study conduct.

The same sequential XAV-19 administrations will apply for the second group dosed at 2mg/kg, with safety steps and DSMC data review.

During the main phase of the study (phase 2b), the DSMC will regularly meets as defined in the DSMC charter.

During the phase 2b study, if the proportion of patients with need of invasive mechanical ventilation and/or death after inclusion reaches to 20% or above (analyzed by incremental groups of 50 patients), the DSMC will be asked to review the data without stopping the study (see 6.6.3). If the proportion of patients dead and/or requiring post-enrolment invasive mechanical ventilation after inclusion reaches 35% of above of the patients included in the study (analyzed by incremental groups of 50 patients), the enrolments will be temporarily discontinued and DSMC will be asked to review the data and decide on an early permanent discontinuation of the study (see 6.6.3).

## Description and justification of the treatment plan

This is a phase 2 study with the aim to define the optimal XAV-19 dose to administrate to moderate COVID-19 patients, and to evaluate its safety and efficacy.

XAV-19 is a glyco-humanized polyclonal Antibody. Glyco-humanized polyclonal Antibodies have been evaluated for safety effects in kidney graft recipient volunteers at the five dose levels of 0.6, 1, 3, 6 and 8 mg/kg administered daily for 5 days. Cumulative doses have thus been 3, 5,15, 30 and 40 mg/kg.

The estimated half-life (T1/2) from pre-clinical non-human primate data is of approximately 5 days, while first assessments from human data predict a half-life reaching 11 days.

The phase 2a will be a single or double ascending dose (DAD), double-blind, placebo-controlled randomized study to select the optimal dose of XAV-19 for the phase 2b. Phase 2a will enroll 16 subjects in one of the two dose-level groups: Group 1 with dose level defined according to the neutralization titer of the XAV19 batch (first estimation being 0.5 mg/kg), Group 2 as 4 times higher than Group 1 (estimated 2.0 mg/kg).

Each subject of the Group 1 and the first two patients of Group 2 will receive two infusions of XAV-19 or placebo separated by an interval of 4 days (administrations on Day 1 and Day 5). Rest of patients from Group 2 will receive only one administration of XAV-19 on Day 1. In each of group 1 and 2, a sequential inclusion strategy will be applied: the first two patients will be randomized in a 1:1 ratio to receive either treatment or placebo, after safety analysis reviewed by the DSMC, the following 6 patients will be randomized to receive either treatment or placebo in a 5:1 ratio. Subjects will be dosed with a minimal interval of 24h (first to first administration). This 24h interval is meant to monitor possible adverse reaction to the product, such as local intolerance, allergy or cytokine release syndrome, that may occur in first hours after infusion^22^.

A predefined hold time of minimum 8 days (first to first administration) will be applied after dosing in group 1, for interim evaluation of safety by the DSMC, on which decisions for the second dose level will be based. Same design with sequential XAV-19 administrations will apply for the Group2 dosed at 2mg/kg.

The phase 2b will be a double-blind, placebo-controlled randomized study to assess clinical benefit and safety of the phase 2a selected dose of XAV-19 in 398 hospitalized adults with COVID-19 associated moderate pneumonia. Patient will be randomized to either XAV-19 or placebo in a 1:1 ratio and will receive a single dose based on the analysis of the Phase 2a study.

In addition to ancillary studies conducted during both parts of the study, a terminal ancillary open label study will be performed after completion of the main phase 2b part to assess a flat dose regimen of XAV-19.

**Dose determination for Phase 2a**

Based on preclinical assessment of the elimination half-life of swine glyco-humanized IgG in non-human primates (baboon), it was hypothesized for the first cohort of Phase 2a of POLYCOR (low dose at 0.5mg/kg) that 2 doses 4 days apart (Day 1 and Day 5) would be necessary to obtain serum neutralizing antibodies titers maintained for 10 days. This period of 10 days was initially considered as the duration of productive SARS-CoV-2 infection during covid-19. Today, the first data from clinical trials evaluating the benefit of polyclonal anti-SARS-CoV-2 immunoglobulins (such as in Hueso et al, Blood, 2020 in immunocompromised patients) indicated that neutralizing antibodies result in clinical benefits within a few days (as low as 3 days). In addition, preliminary data of XAV-19 PK in the first 3 patients who received the low-dose of 0,5 mg/kg during the first cohort of POLYCOR Phase 2a, suggest that effective concentrations will be obtained for more than a week with a single higher dose, without need for a second dose administration. This has been confirmed with preliminary PK data in the first 4 patients who received a single dose of Xav-19 of 2mg/kg in the second part of the phase 2a. Concentrations were above the effective target concentration of neutralizing specific antibodies (10,000 ng/ml), with a mean peak post-infusion concentration around 50,000 ng/ml, and concentrations maintained >15 days above target. Maintaining a pharmacodynamic effect of neutralizing antibodies over more than a week is considered as an appropriate period of time to improve recovery and diminish worsening.

Dose levels selected in the First In Human assessment of XAV-19 are based on 3 datasets:

1) Assessment of the higher acceptable dose: Available clinical experience (EUDRACT 2019-000917-35) indicate than swine glyco-humanized antibodies are well tolerated at doses so far tested up to 40mg/Kg (cumulative dose after 5 daily administrations of 8 mg/Kg). Therefore, in this First In Human trial with XAV-19, the cumulative dose is proposed to be inferior to this so far tested upper limit.

2) Assessment of the Pharmacologically Active Dose: Experience with transfusion of convalescent patient plasma (CP) indicated a benefit if containing anti-COVID-19 neutralizing antibodies at titers higher than 1/40 and given at a dose equivalent to 25 mg/Kg IgG. The neutralizing titer of XAV-19 is at least 40-fold higher than COVID-19 CP. Accordingly, infusions of 0.6 mg/Kg XAV-19 should result in similar effects in vivo as compared with 25 mg/Kg CP.

3) PK/PD information obtained from ex-vivo assessment of XAV-19. XAV-19 potency has been assessed in competition ELISA (inhibition of the COVID-19 spike protein binding to ACE-2 receptor) and tested in cytopathogenic effect assay (CPE) using SARS-CoV-2 infection of human Vero cells. Data showed an IC50 (concentration for reaching 50% of maximal signal in the test) of approx. 2.5 µg/mL of XAV-19 in ELISA and 10 µg/ml in the CPE assay. The volume of distribution of swine glyco-humanized IgG measured in primates (53 mL/kg) has a value close to that of the plasma suggesting that the XAV-19 IgGs will essentially circulate in the plasma. Considering an average plasma volume in human of 48 ml/Kg, infusions of 0.5 mg/Kg of XAV-19 will translate into 10 µg/mL plasma concentration (at Cmax). Infusions of 2 mg/Kg of XAV-19 will translate to approx. 40 µg/mL plasma concentration at Cmax. The elimination half-life of equivalent glyco-humanized antibodies was estimated in primates at 172h.

Based on these 3 datasets, **the proposed dose level for Group 2 of phase IIa, would be 2 mg/Kg infused on day 1 (or on day 1 and day 5 for some patients)**. It is meant to maintain trough levels of XAV-19 well above the threshold of 10 µg/mL (considered being a neutralizing threshold) for at least a week. This dosing (one administration of 2 mg/Kg) presents a 10 to 20-fold safety factor for the administration of XAV-19, as compared with previous experience with glyco-humanized swine IgG.

Although data discussed above suggest that a single dose of 2 mg/kg will be sufficient to provide clinical activity and support administration of this single unique dose in the second cohort, the first two patients of this second cohort (1 XAV-19, 1 placebo) will receive 2 doses of 2 mg/kg, as initially planned, to confirm relevant PK/PD information and safety information before moving to the single high dose administration to the rest of the cohort (n = 6). It can be argued that avoiding 2 doses will diminish non necessary potential safety issues and will facilitate hospital discharge in patients with rapid recovery.

All patients will be closely monitored by a data safety monitoring committee (DSMC) during the study for emerging efficacy and overall safety data to ensure that the benefits and risks of study participation remain acceptable. Strict rules have been defined to recommend the discontinuation of XAV-19 dose at any time during the study in case of safety (Section 4.1.2.1, Dose Adjustment).

**Dose determination for Phase 2b**

The evaluation of safety and pharmacokinetic parameters in both dosing groups will guide the choice for the dose to be used in the Phase 2b study. This dose will range between 0,5 and 2 mg/kg given in a single administration. Alternatively, a corresponding flat dose might be considered.

Based on good safety and tolerability of phase 2a and PK data with the higher dose, a single infusion of 2 mg/kg of Xav-19 was selected for phase 2b.

Furthermore, a corresponding flat dose regimen (fixed dose of 150mg XAV-19) will be assessed in the terminal ancillary open study which will be performed on 20 patients. This regimen is based on:

- The preclinical data acquired during the development of XAV-19 compared to the phase 2a data: indeed, a XAV-19 concentration of approximatively 1 to 5 μg/ml is required to fully neutralize all variants (Wuhan, British and South-African forms)^23^. The Phase 2a has demonstrated that a single intravenous perfusion of XAV-19 at 2 mg/kg was safe, achieving a median serum Cmax of 50.4 μg/ml and D8 concentration of 20.3 μg/ml with an elimination half-life (T1/2) estimated at 11.4 days (Gaborit B et al MedRxIV, 2021, article in progress). All this data indicate that XAV-19 can provide high and sustained therapeutic activity with an inhibitory quotient well above 10.
- The phase 2a clinical data: based on patients’ median weight which was 77.3 Kg ([72-98] range from 52 to 120), a fixed dose of 150mg of XAV-19 will therefore be equivalent to a median dose of 1.94 mg/Kg; ([2.08-1.53 Kg]; Range 2.88-1.36*) (*to note, above 110 Kg, blood volume no longer increases proportionally to mass). If we consider 2 extremes body weights: a fixed dose of 150 mg in a 50 kg patient corresponds to a dose of 3 mg/Kg, a dose that theoretically results in a Cmax of 75 µg of XAV-19 per ml of plasma; a fixed dose of 150 mg in a 120 kg patient corresponds to a dose of 1.25 mg/Kg resulting in a Cmax of approximately 30 µg of XAV-19 per ml of plasma. These two extreme cases show that the therapeutic index will remain high, varying from 6 to 15 times the neutralizing concentration. The therapeutic window is therefore wide with XAV-19. These examples also show that there will be no exposure above the upper limit of 40 mg/Kg, a dose that has been administered in humans without any product-related adverse events being noted (data extrapolated from the ongoing LIS1 trial where another GH-pAb is administered; NCT 04431219).

Furthermore, initial volume of distribution and clearance data from phase 2a suggest that the intensity of diffusion of XAV-19 in the body does not increase with patient weight and the rate of elimination of XAV-19 from the blood does not increase ( or decrease ) with patient weight (Gaborit B et al MedRxIV, 2021, article in progress).

- Lastly, the literature indicates that for therapeutic Abs, in contrast to the conventional assumption, body weight-based dosing does not always offer advantages over fixed dosing in reducing exposure variability.^24^

These data lead us to investigate a fixed dose of XAV -19 for comparison with conventional weight adjusted dose as tested so far in the phase 2b patients.

**The bibliographical references are appended to the document.**

# Objectives and endpoints of Phase 2a

## Primary objective and endpoint

### Primary objective

The primary objectives of the study are to:

1. evaluate the Antibody titers of XAV-19 treated patients versus placebo treated patients at Day 8
2. assess the safety and tolerability of XAV-19.

### Primary endpoint

The primary endpoints are the:

1. pharmacokinetic measurement of the antibody titer of XAV-19 measurements of all treated patients and of all patients in the placebo group at Day 8
2. tolerability and adverse events of XAV-19 between the two groups of treated patients and vs. placebo over 29 days, evaluated as:

- Occurrence of all suspected XAV-19 related adverse effects and incidence of serious adverse events
- Proportion of participants with treatment emergent adverse events leading to study drug discontinuation
- Incidence of major or bacterial or fungal infections
- Incidence of hypersensitivity reactions and infusion reactions
- White cell count, hemoglobin, platelets, creatinine, ALT, AST, on D1, D3, D5, D8, D15 and D29
- SARS-CoV-2 viral load over time (D1-D29), as collected by nasopharyngeal swab samples
- Time to RT-PCR virus negativity in nasopharyngeal swab samples

## Secondary objectives and endpoints

### Secondary objectives

The secondary objectives of the study are:

1. To characterize pharmacokinetics (PK) of XAV-19 infected patients over the time from D1 to D29
2. To evaluate the Antibody titers of XAV-19 and to compare Group 1 treated patients versus Group 2 treated patients at Day 8.
3. To describe groups of patients according to clinical variables (Duration of supplemental oxygen, transfer in ICU, Normalization of fever, titer of Biomarkers, Ordinal scale assessed at Day 15, Hospital length of stay).

### Secondary endpoint(s)

The secondary endpoints are the:

1. Pharmacokinetic analysis: Antibody titer measurements at Day 1 (pre-dose, post-dose), Day 3, Day 5 (if applicable second XAV-19 administration): pre-dose and post-dose), Day 8, Day 15, and Day 29.
2. The antibody titer of XAV-19 measurements in Group 1 treated patients and Group 2 treated patients at Day 15.
3. Clinical aspects:

- Duration of supplemental oxygen
- Transfer to intensive care unit with need for invasive mechanical ventilation or high flow oxygen
- Normalization of fever ≥ 24 hours: clinical assessment every day from Day 1 to Day 14. Evaluation to be performed between 8 and 12 am, Day X evaluation will consider the higher value during Day X-1 (8 am to 8 am)

- Normal fever (≤ 37.8° C tympanic or ≤ 36.6°C axillary or ≤ 37.2°C oral taken 4 hours apart from antipyretic administration) each day and time to resolution of fever for at least 48 hours

- Biomarkers : CRP, Ferritin
- Ordinal scale assessed at Day 15. The 7-point ordinal scale is an assessment of the clinical status at the first assessment on day 15. The scale is as follows:

1. Not hospitalized, no limitations on activities;
2. Not hospitalized, limitations on activities;
3. Hospitalized, not requiring supplemental oxygen;
4. Hospitalized, requiring supplemental oxygen;
5. Hospitalized, on non-invasive ventilation or high flow O2 devices;
6. Hospitalized, on invasive mechanical ventilation or ECMO;
7. Death.

- Hospital length of stay

## Objective and endpoints for exploratory studies for phase 2a

Exploratory objectives will be to assess the effects of neutralizing antibodies use on virus-induced immune response on longitudinal follow-up, and to identify targets for "immuno-monitoring" for the next phase of the study and to investigate the immunogenicity of COVID-19 during treatment with XAV-19.

We will take advantage of the existing "Immunologie" research program” declared by Nantes CHU to set up the biocollection “POLYCOR”. This biocollection will be a biological and longitudinal follow-up of patients with diagnosis of COVID-19, to assess immune changes and host-virus interactions. Ethic authorization is already granted, and the preanalytical steps are already implemented and efficient. Blood samples (plasma, serum at -80°C, PBMCs) are stored in liquid nitrogen.

Blood samples taken will be stored in this biocollection until analyses.

Objectives of Collections of samples:

HD monitoring of circulating immune cells:

Two mAb panels validated by Cytek (https://cytekbio.com/pages/aurora#tab-data ) will be used for lymphocytes and MNP (mononuclear phagocytes) populations respectively.

1. Panel 1 - 35 Abs:

CD45RA/CD16/CCR5/CD11C/CD56/CD8/CCR7/CD123/CD161/IgD/CD3/CD20/IgM/IgG/CD28/CXCR3/CCR6/CXCR5/PD-1/CD141/CD57/CD14/CD45/CD11b/TCRgd/CD25/CD24/CD95/CXCR3/CD27/CD1c/CD127/HLA-DR/CD19/CD4/CD38.

1. Panel 2 -28 Abs:

CD40/CD16/CD56/CD3/CD86/CD45/CX3CR1/CD66b/CD85k/FCER1a/PD-L1/CD141/CD172a/CD14/CD11b/CD123/CD26/CD32/CD197/CD163/CD80/CD19/CD4/CD272/CD1c/CD11c/HLA-DR.

Both supervised and unsupervised analyses will be performed.

Serum cytokine/chemokine profiles in XAV19 vs. placebo COVID-19 pneumonia:

We will measure the concentration of MCP1, IP10, IL-10, IFNa, TNF-α, IL-1b, IL-6, IL-82 (in the phase 2a) and IL-6, IFNa2, IL-10, IP10, CCL2 (in the phase 2b).

Ex vivo pharmacodynamic:

Ability of patient serum to neutralize binding of recombinant SARS-CoV-2 Spike molecule to ACE-2 receptor in an ELISA format.

Description of transcriptomic analyses:

Messenger RNA molecules will be extracted from peripheral blood in order to identify transcripts that are specifically over- or under-expressed in correlation with the clinical status and with viral load. The goal is to identify possible biomarkers of the disease severity and disease responding to the treatment, and, using deconvolution bioanalyses, also identifying cell population changes in complement to the direct flow cytometry analyses. A focus will be made on B cell subpopulation and their role in interaction with Tfh cells.

# Objectives and endpoints of Phase 2b

## Primary objective and endpoint

### Primary objective

To evaluate the efficacy of XAV-19 + standard-of-care (Soc) therapy compared with placebo

+ Soc therapy for treatment of COVID-19 assessed by the proportion of patients who die or develop respiratory failure between baseline and Day 15.

### Primary endpoint

Patients who died or develop respiratory failure, as defined by the requirement of noninvasive ventilation, high-flow oxygen devices, invasive mechanical ventilation (corresponding to a score of 5 or more on the WHO 8 point ordinal scale) or by an increase of the required O2 supplement more or equals to 10 L/minutes with a non-rebreather mask (oxygen mask with reservoir bag).

## Secondary objectives and endpoints

### Secondary objectives

**The key secondary objectives** are to evaluate the efficacy of XAV-19 + standard-of-care (Soc) therapy compared with placebo + Soc therapy for treatment of COVID-19 between baseline and Day 8, and then between baseline and Day 29.

**The others secondary objectives** are to evaluate clinical efficacy and safety of the investigational therapeutic compared to the control arm, assessed by:

**Clinical severity**:

a) National Early Warning Score (NEWS) over 29 days:

*-* Change from baseline to Day 3, 5, 8, 15 and 29

- Time to discharge or a NEWS <=2 and maintained for 24 hours

b) 8-point ordinal scale over 29 days

- Time to improvement of one category from baseline (Day 1)

- Mean change in ordinal scale from baseline to Day 3, 5, 8, 15 and 29

- Percentage of subjects reporting each severity rating on an 8-point ordinal scale at Day15

c) Improvement of clinical and biological parameters over Day 15

d) Oxygenation

- Duration of oxygen therapy over 29 days

- Time to weaning in supplemental oxygen

- Proportion without O2 requirement at D8, D15 and D29

e) Non-invasive ventilation, high-flow oxygen

*-* Non-invasive ventilation/high flow oxygen use up to Day 29

- Incidence and duration of non-invasive ventilation or high flow oxygen use during the study

- Time to first day with non-invasive ventilation/high flow oxygen

f) Invasive mechanical ventilation / Extra Corporeal Membrane Oxygenation (ECMO)

- Ventilator / ECMO use up to Day 29

- Incidence and duration of new mechanical ventilation or ECMO use during the study

- Time to first day with Invasive Mechanical Ventilation / extracorporeal membrane oxygenation

g) Transfer to ICU by Day 29

h) Hospitalization

- Hospital length of stay (in days)

i) Mortality

- Mortality rate at day 15 and at Day 29 and at Day 60

j)Thrombotic events

-°cumulative incidence of thrombotic events : venous, pulmonary embolism, arterial thrombotic events.

**Safety of XAV-19**

Evaluate the safety of the intervention through 29 days and 60 days of follow up as compared to the control group as assessed by:

- The cumulative incidence of SAE
- The cumulative incidence of grade 3 or 4 AE
- Proportion of discontinuations or temporary suspensions of infusion (for any reason)
- Changes in white cell count, hemoglobin, platelets, creatinine, ALT, AST, over time
- The cumulative incidence of major or opportunistic bacterial or fungal infections
- The cumulative incidence of hypersensitivity reactions and infusion reactions

**Exploratory analysis on a subset of patients**

- Change in SARS-CoV-2 status (positive or negative, and quantitatively, including variant information by sequencing) viral load over time
- Time to RT-PCR virus negativity in nasopharyngeal swab samples over 29 days

### Secondary endpoint(s)

**The key secondary endpoints are the**:

Proportion of patients who die, develop respiratory failure, as defined by the requirement of noninvasive ventilation, high-flow oxygen devices or invasive mechanical ventilation at Day 8 and Day 29.

**The other secondary endpoints are:**

1. National Early Warning Score (NEWS) assessed while hospitalized and on Day 15 and Day 29
2. Clinical status using the 8-point ordinal scale assessed daily ~~during hospitalization~~ until Day 29
3. Temperature and blood analysis between baseline and Day 15, and Day 29
4. Days of oxygen therapy over 29 days

PaO2 / FiO2 at baseline, Day 5, Day 8, Day 15, Day 29 if available

1. Days of non-invasive ventilation or high flow oxygen (if applicable) up to Day 29
2. Days of invasive mechanical ventilation/ECMO (if applicable) up to Day 29
3. Transfer in ICU
4. Hospital length of stay (in days)
5. All-cause mortality evaluated between baseline and Day 15 and between baseline and at Day 29 and at Day 60
6. Thrombotic events (peripheral venous, pulmonary, arterial)

**Safety of XAV-19 evaluated as:**

- Occurrence of all suspected XAV-19 related adverse effects or Incidence of serious adverse events
- Study drug discontinuation or temporary suspension of infusion
- Proportion of participants with treatment emergent adverse events leading to study drug discontinuation
- Incidence of major or opportunistic bacterial or fungal infections
- Incidence of hypersensitivity reactions and infusion reactions
- White cell count, hemoglobin, platelets, creatinine, ALT, AST, on D1, D3, D5, D8, D15 and D29

**Exploratory analysis**

- SARS-CoV-2 status (positive or negative and quantitatively, including variant information by sequencing) over time (D1, D8, D15, and D29)
- SARS-CoV-2 status viral load over time (D1, D8, D15, and D29)

## Objective and endpoints for ancillary studies for phase 2b

Three ancillary studies will complete the phase 2b. For the pharmacokinetic study, 90 patients will be included, as for the immunomonitoring study 100 patients will be included. Finally, at the end of phase 2b study, 20 patients will all receive 150mg of
XAV-19. Analyses performed will be identical to those performed in the phase 2a study.

Study sites involved in these ancillary studies will propose to their first patients to participate to these studies. For the first two studies mentioned patients can participate simultaneously to both studies.

### Pharmacokinetic Study (90 patients)

#### Objective

The objective of the pharmacokinetic study is to characterize pharmacokinetics (PK) of XAV-19 infected patients over the time from D1 to D29.

#### Endpoint

Pharmacokinetic analysis correspond to antibody titer measurements at Day 1 (pre-dose, post-dose), Day 3, Day 5, Day 8, Day 15, and Day 29.

### Immunomonitoring Study (100 patients)

#### Objective

Objectives will be to assess the effects of neutralizing antibodies use on virus-induced immune response on longitudinal follow-up, and to identify targets for "immuno-monitoring" for the next phase of the study and to investigate the immunogenicity of COVID-19 during treatment with XAV19.

#### Endpoint

The endpoints encompass the following analysis:

- Spike/ACE2 neutralizing antibody titers: D1 (pre-, post dose), D3, D5, D8, D15 and D29
- Lymphocytes sub-population: D1, D3, D5, D8 and D15
- Transcriptomic analyses: D1, D3, D5, D8 and D15
- Cytokines: D1, D3, D5, D8 and D15

### Terminal ancillary Study (20 additional patients)

#### Objective

The objectives are:

-to compare pharmacokinetic parameters in patients receiving a fixed dose of 150mg with patients receiving 2mg/Kg of XAV-19 (master phase 2b), in order to confirm that the exposure and variability are similar

-to compare the effects of neutralizing antibodies use on virus-induced immune response on longitudinal follow-up, and targets for "immuno-monitoring"

-to investigate the immunogenicity of COVID-19 during treatment with XAV19 in patients receiving a fixed dose of 150mg with patients receiving 2mg/Kg of XAV-19

#### Endpoint

Antibody titer measurements IN SERUM at Day 1 (pre-dose, post-dose), Day 3, Day 5, Day 8, Day 15, and Day 29

The endpoints encompass the following analysis:

- Spike/ACE2 neutralizing antibody titers: D1 (pre-, post dose), D3, D5, D8, D15 and D29
- Lymphocytes sub-population: D1, D3, D5, D8 and D15
- Transcriptomic analyses: D1, D3, D5, D8 and D15
- Cytokines: D1, D3, D5, D8 and D15

# Study treatment

In this study, XAV-19 is the Investigational Medicinal Product (IMP), Xenothera will be responsible for providing XAV-19 to Nantes University Hospital.

## Description and mode of administration

### Product Characteristics

- Physical and chemical characteristics:

XAV-19 is a glyco-humanized swine polyclonal IgG antibody targeting SARS-CoV-2 spike Receptor Binding Domain (RBD). It is composed of IgG molecules with a molecular mass of 150 kilodaltons (kDa). Glyco-humanization refers to replacement of animal-type Gal alpha 1-3 galactose (alpha-Gal) and N-glycolylneuraminic acid (Neu5GC) glyco epitopes by terminal glyco-epitopes of the human type (Neu5AC sialylation). XAV-19 is produced by immunization of genetically-modified swine in which the alpha 1,3-galactosyltransferase (GT1) and the cytidine monophosphate N-acetyl hydroxylase genes have been knocked out. Immunization has been performed with a recombinant protein comprising the Receptor Binding Domain (RBD) of the SARS-CoV-2 spike molecule. Swine are bred under Defined High Health Status (DHHS) conditions, suitable to produce parenteral pharmaceutical substances. Glyco-humanization reduces immunogenicity, prevents allergy due to recognition of alpha-Gal moieties by anti-alpha-Gal IgE and prevents formation of immune complexes that might arise as a result of recognition by pre-existing anti-Neu5GC natural antibodies, such complexes potentially causing serum sickness. XAV-19 binds to and blocks S1-dependent viral entry into ACE-2 positive human cells. Because it is a swine antibody, IgG Fc domains of XAV-19 do not bind to human Fc receptors and therefore XAV-19 is predicted to not being able to elicit antibody-dependent enhancement, i.e. antibody-dependent promotion of entry of viral particles into Fc receptor-positive human cells.

- Formulation

The drug product is provided as a sterile solution in a single use.

XAV-19 is a novel investigational drug formulated in a buffered solution of phosphate-buffered saline containing 0.05% polysorbate. Each ampoule is filled with a target volume of 5.5 mL which includes a 10% overfill to allow an extractable volume of 5 mL drug product containing 25 mg of XAV-19 (5 mg/mL).

The drug product should be stored at 2-8°C and is stable at this temperature for at least 6 months for the Phase 2a and 12 months for the Phase 2b. The shelf-life will be extended when actual data will become available. Swine GH-pAbs are projected to be stable at 4°C for > 3 years.

### Administration

The drug product is intended to be slowly administered by IV infusion after dilution in a NaCl 0.9% bag. The dilution will be based on the patient weight and the dose must be administered using in-line filtration.

For Phase 2a, XAV-19 will be given:

- at the dose of 0.5 mg/kg intravenously (IV) on Day 1 (D1) and Day 5 (D5) (i.e., 2 injections in total) in group 1.
- at the dose of 2 mg/kg intravenously (IV) on Day 1 (D1) and Day 5 (D5) (i.e., 2 injections in total) for the first two patients and on D1 only (i.e., one administration) for the rest of the group, in group 2.

XAV-19 will be administered by slow intravenous infusion of 100 mL over at least 30 min but not to exceed 60 min, after dilution to the right concentration in sterile NaCl 0.9% pocket.

Timing of infusion will have to be between 9 am and 4 pm at Day 1, and, as much as possible, at the same time as in Day 1, for Day 5 if applicable.

For Phase 2b, XAV-19 will be given at the dose chosen from the Phase 2a intravenously (IV) on Day 1 (D1), i.e 2mg/kg.

XAV-19 will be administered by slow intravenous infusion of 100 mL over at least 30 min but not to exceed 60 min, after dilution to the right concentration in sterile NaCl 0.9% pocket.

Timing of infusion will have to be between 9 am and 4 pm at Day 1

Additional detailed information on dose preparation and administration will accompany the clinical drug supplies to the clinical study sites.

For the terminal ancillary study, XAV-19 will be given at a fixed dose of 150mg, as single infusion.

#### Dose adjustment

In the absence of serious or grade 4 adverse events deemed at least possibly related to study drug treatment, patients will complete their scheduled infusions as prescribed by protocol.

In the event of new safety findings, serious and unexpected toxicity potentially related to XAV-19, study drug administration should be interrupted, and in case, only 1 dose has been administered, the second dose if applicable will not be administered (no dose adjustment). The investigator must immediately notify the adverse event/ new safety finding to the sponsor.

#### Reference documents

To date, no serious adverse reactions are considered expected for the purpose of expedited reporting of suspected unexpected serious adverse reactions (SUSARs).

For more information on the safety profile of XAV-19, please refer to the current version of the Investigator Brochure; section RSI (Reference Safety Information).

### Other study treatments

No other study treatment will be administered in the study.

## Treatment compliance follow-up

All medications specified in this protocol must be administered as described within the protocol.

The administration of XAV-19 must be done in the investigational site and supervised by a physician or a nurse of the department. The investigator and/or the pharmacist must retain adequate and accurate records of XAV-19, showing the receipt and distribution of the clinical supplies, and any deviations from specified administration should be clearly documented in the patient record.

## Experimental drug circuit

### General circuit

Labelled XAV-19 vials (IMP) are provided by Xenothera to Nantes University Hospital Pharmacy. Each vial contains 25 mg of XAV-19. Each ampoule is filled with a target volume of 5.5 mL which includes a 10% overfill to allow an extractable volume of 5 mL drug product containing 25 mg of XAV-19 (5 mg/mL).

Nantes University Hospital Pharmacy will manage XAV-19 vials: receipt, storage, relabelling (if applicable), distribution to the other sites hospital pharmacies or dispensing directly to investigational site (Nantes Hospital), accountability, and disposal (after sponsor approval).

The other sites hospital pharmacies will manage XAV-19 vials: receipt, storage, relabelling (if applicable), dispensing directly to investigational site, accountability, and disposal (after sponsor approval).

Each pharmacy will ensure IMPs reconciliation and accountability.

XAV-19 will be diluted in an infusion bag containing a variable volume (weight-dependent) of NaCl at the concentration of 0.83 mg/ml for groups 1 and 2 by each pharmacy department upon request of the investigational site.

The XAV-19 infusion bag will then be transferred to the investigational site for administration to the patient.

### Experimental drug storage conditions

The investigator (or the Hospital Pharmacy_PUI) is responsible for the safe storage of all study drugs assigned to the clinical site, in a secure place with restricted access, and maintained within the appropriate ranges of temperature.

Regular temperature logging of the study drug storage room should be performed. In case of temperature excursion during storage conditions, the pharmacy/site must notify immediately the sponsor. The pharmacy/site must wait for Sponsor approval, according to Xenothera’s recommendations, to dispense the affected study drug.

#### Description of dispensary storage

As of reception by the Hospital Pharmacies, IMPs are kept in a cool dry place, protected from light, where the temperature stays between +2 and +8°C.

The vials will be stored in a secured place whose temperature is monitored, stored away from conventional drugs.

#### Description of department storage

Once reconstituted in the NaCl bags, the pharmacy ensures the dispensing of IMPs to investigator’s team. XAV-19 pocket will be stored in the investigator site between +2 and +8°C until administration. Bags should be administered within 72h after reconstitution. Empty bags will be destroyed at clinical site.

### Unblinding procedure

Under normal circumstances, blind should not be broken until all patients have completed the study and the database is locked.

Phase 2b :

For the first set of statistical analyses, to allow early reporting of primary and secondary endpoints at D15, the blind will be partially broken once all patients have completed Day 29. Except for statisticians, only the principal investigator and the scientific coordinator will have access to the full data set for the analysis of the primary and secondary endpoints up to day 29. The database will be partially locked (with all data up to day 29) as neither monitors nor investigators will be informed of the unblinding until the final data for day 60 is completed and the final database is locked.

In the current study, the investigator does not need to know the treatment arm of the patient in case of adverse event, as the treatment will be symptomatic and not adapted to the arm. The blind will be broken only by the pharmacovigilant team for safety purposes and transmission.

This will be done according to the internal sponsor’s procedure.

For the terminal ancillary study, the study will be conducted as open label.

## Authorised and unauthorised treatments

### Authorised treatments

Standard of Care (SOC) for COVID-19 is defined by use of dexamethasone and per local practice and/or local/national guidelines at time of the study, which may include, not exclusively, antibiotics, antiviral treatment, corticosteroids, immune therapies not based on antibodies administration, anticoagulants.

### Unauthorised treatments

Serotherapy for the current COVID-19 episode will not be permitted.

Patients with prior anti-COVID-19 vaccine are not excluded whatever the delay.

Anti-COVID-19 vaccine is not permitted in the study, it is recommended to wait 90 days after the acute covid-19 episode, before proposing an anti-COVID-19 vaccination so as not to risk reducing the immunogenicity of the vaccine.

# Study population

The population to be studied is adults (male or female), over 18 years old, hospitalized for moderate COVID-19 as confirmed by a positive RT-PCR. The patients will be recruited in the investigational sites declared on this study.

Women of childbearing potential (WOCBP) must use appropriate method(s) of contraception during the clinical trial (oral contraception, implant or IUD).

Pregnant woman or Women of childbearing potential (WOCBP) without effective contraception or nursing, patient under guardianship or trusteeship will not be included in the study.

The subjects cannot participate simultaneously in another interventional study and until 3 months after the end of their participation to this study.

## Description of the population in Phase 2a

In the phase 2a, two sequential groups:

- Dose 1 XAV-19 with 0.5 mg/kg (n=6) versus Placebo (n=2)

8 patients receiving two doses of treatment (day 1, day 5) and follow-up until D29

- Dose 2 XAV-19 with 2 mg/kg (n=6) versus Placebo (n=2)

2 first patients receiving two doses of treatment (day 1, day 5) and 6 patients receiving one dose of treatment at 2mg/kg at D1 and all have follow-up until D29

16 patients will be included.

If a patient does not receive the appropriate treatment or has not been followed for 8 days then he will be replaced within the limit of 20 patients in total.

Patients will be treated once or twice in 1 week with XAV-19 (at day 1 and day 5 or only at day 1) and monitored regularly during the study period (see flow chart). Patients will be hospitalized at least from Day 1 to Day 3, if discharged before Day 5 the patient will come back for on-site visits at Day 5, Day 8, Day 15 and Day 29. Visits on Day 4, Day 6, Day 7, Day 11 and Day 13 will be done via phone calls to evaluate the clinical status, collect any adverse event and any new concomitant medications.

### Inclusion criteria

1. Willing and able to provide written informed consent prior to performing study procedures
2. Male or female ≥ 18 years and ≤ 85 years
3. Hospitalized for COVID-19
4. Positive SARS-CoV-2 RT-PCR in any body specimen (nasopharynx, saliva, sputum) ≤ 10 days before enrolment
5. Evidence of pulmonary involvement (on lung examination [rales/crackles] and/or chest-imaging [Chest X-ray or computed tomography])
6. Requiring O2 supplement ≤ 6L/min at screening
7. Requiring O2 supplementation with SpO2 ≥ 92% on O2 therapy at screening
8. First onset of COVID-19 symptoms ≤ 10 days, among fever and/or chills, headache, myalgias, cough, shortness of breath, whichever as occurred fist
9. WOCBP must have a negative urinary pregnancy test the day of inclusion
10. All sexually active male subjects must agree to use an adequate method of contraception throughout the study period and for 90 days after the last dose of study drug and agree to no sperm donation until the end of the study, or for 90 days after the last dose of XAV-19, whichever is longer
11. Patients with French social security

### Exclusion criteria

1. Evidence of multiorgan failure (severe COVID-19)
2. Mechanically ventilated (including ECMO)
3. Receipt of immunoglobulins or any blood products in the past 30 days
4. Psychiatric or cognitive illness or recreational drug/alcohol use that in the opinion of the investigator, would affect subject safety and/or compliance
5. End-stage renal disease (eGFR < 15 ml/min/1,73 m^2^)
6. Child-Pugh C stage liver cirrhosis
7. Decompensated cardiac insufficiency
8. **History of active drug abuse**
9. Known allergy, hypersensitivity, or intolerance to the study drug, or to any of its components
10. Females of childbearing potential without contraceptive method, or with positive pregnancy test, breastfeeding, or planning to become pregnant during the study period
11. Current documented and uncontrolled bacterial infection.
12. Prior severe (grade 3) allergic reactions to plasma transfusion
13. Patient participating in another interventional clinical trial
14. Life expectancy estimated to be less than 6 months
15. Patient under guardianship or trusteeship

## Description of the population in Phase 2b

In the phase 2b, about 398 patients, 199 patients in each group, will be included in this study.

Patients will be treated once with XAV-19 (at day 1) and monitored regularly during the study period (see flow chart). Patients will be hospitalized at least from Day 1 to Day 3, if discharged before Day 5 the patient will come back for on-site visits Day 8, Day 15, except if patient is participating to the ancillary study, in which case he will have to come back on site for the Day 5 and Day 29 visits.

In the terminal ancillary study, 20 patients will be included with the same inclusion and exclusion criteria as the other phase 2b patients. Patients will be treated once with XAV-19 (at day 1) at flat dose and monitored regularly during the study period (see flow chart). Patients will be hospitalized at least from Day 1 to Day 3, if discharged before Day 5 the patient will come back for on-site visits Day 5, 8, 15 and 29.

### Inclusion criteria

1. Willing and able to provide written informed consent prior to performing study procedures
2. Male or female ≥ 18 years
3. Hospitalized for COVID-19
4. Documentation of SARS-Cov-2 infection before enrolment, by positive SARS-CoV-2 RT-PCR or antigen in any body specimen (nasopharynx, oropharynx, saliva, sputum, bronchoalveolar lavage …) before enrolment
5. Evidence of pulmonary involvement (on lung examination [rales/crackles] and/or chest-imaging [Chest X-ray or computed tomography])
6. Requiring O2 supplement ≤ 6L/min at screening
7. Requiring O2 supplementation with SpO2 ≥ 92% on O2 therapy at screening (or ≥ 90 % if chronic obstructive pulmonary disease)
8. First onset of COVID-19 symptoms ≤ 14 days, among fever and/or chills, headache, myalgias, cough, shortness of breath, whichever as occurred fist (other symptoms such as asthenia not to be considered in this list)
9. WOCBP must have a negative urinary pregnancy test the day of inclusion
10. All sexually active male subjects must agree to use an adequate method of contraception throughout the study period and for 90 days after the last dose of study drug and agree to no sperm donation until the end of the study, or for 90 days after the last dose of XAV-19, whichever is longer
11. Patients with French social security

### Exclusion criteria

1. Evidence of multiorgan failure (severe COVID-19)
2. Mechanically ventilated (including ECMO)
3. Receipt of immunoglobulins or any blood products in the past 30 days
4. Psychiatric or cognitive illness or recreational drug/alcohol use that in the opinion of the investigator, would affect subject safety and/or compliance
5. End-stage renal disease (eGFR < 15 ml/min/1,73 m^2^)
6. Child-Pugh C stage liver cirrhosis
7. Decompensated cardiac insufficiency
8. Known allergy, hypersensitivity, or intolerance to the study drug, or to any of its components
9. Females of childbearing potential without contraceptive method, or with positive pregnancy test, breastfeeding, or planning to become pregnant during the study period
10. Current documented and uncontrolled bacterial infection.
11. Prior severe (grade 3) allergic reactions to plasma transfusion
12. Patient participating in another interventional clinical trial
13. Life expectancy estimated to be less than 6 months
14. Patient under guardianship or trusteeship
15. Patient already included
16. Prior hospitalisation in intensive care unit for the current covid-19 episode

Patients with prior anti-COVID-19 vaccine are not excluded whatever the delay.

Anti-COVID-19 vaccine is not permitted in the study, it is recommended to wait 90 days after the acute covid-19 episode, before proposing an anti-COVID-19 vaccination so as not to risk reducing the immunogenicity of the vaccine.

# Study design and conduct

## Study schedule for phase 2A

The study schedule is summarized in the flowchart below; all of these examinations apply to all patients from phase 2a, the blood collections apply for all patients.

### Screening Visit (Day –2 / Day 1)

Subjects will be screened within 2 days before randomization and dosing to determine eligibility for participation in the study. Following presentation of the study and the informed consent form by the investigator research team, participants will have enough time to take their decision regarding their participation.

- Obtain written informed consent. This should be documented in the medical file of the patient. After the informed consent, the following assessments are performed to determine eligibility requirements as specified in the inclusion and exclusion criteria:
- Focused medical history including the following information (eg, date of first symptoms, overall symptoms, exposure source, demographics, baseline characteristics, allergies and medical history)
- Review and record medications and therapies for this current illness
- Counsel subjects to use adequate birth control methods required during the trial to avoid pregnancy
- Targeted physical examination including, vital signs (heart rate, temperature, blood pressure, level of consciousness), body weight, and height
- Documentation of respiratory status: respiratory rate, oxygen supplementation, SpO2 at rest or PaO2, radiographic findings
- Obtain blood samples if not done in the preceding 48 hours for white blood cell count, hematocrit, platelets, creatinine and creatinine clearance, glucose, total bilirubin, ALT, AST
- Urinary Pregnancy test (for women of childbearing potential)
- Administration of the 7-point Ordinal scale
- Record any serious adverse events and all adverse events related to protocol-mandated procedures occurring after signing of the consent form.

Study subjects who qualify should be immediately randomized, which could occur on same day as screening (Day 1). Randomization and dosing should occur on the same day if possible.

### Inclusion Visit (Day 1)

The following evaluations are to be completed at the Day 1 visit.

The investigator must have confirmed eligibility before proceeding with randomization on the Day 1 visit.

If the screening and Day 1 visits occur within 24 hours, blood analysis and urinary stick do not need to be repeated.

Investigators must complete the following assessments before administration of study drug:

**Before XAV-19 infusion**

- Full clinical examination
- Vital signs
- ECG
- Documentation of respiratory status: respiratory rate, oxygen supplementation, SpO2 at rest or PaO2, radiographic findings (if available)
- Nasopharyngeal swab collection for RT-PCR SARS-CoV-2 (unless diagnosis PCR was done at the same hospital)
- 7-point ordinal scale
- Blood analysis and urinary stick (unless screening and inclusion are the same day)
- Blood sample collection before the administration of XAV-19 (if applicable):
  - - Pharmacokinetic samples
    - Spike/ACE2 interaction neutralizing antibody sample
    - Lymphocytes subpopulation
    - Cytokines
    - Transcriptomic analyses
- Document concomitant medications
- Record any serious adverse events and all adverse events

Following these procedures:

- Randomisation to one of the two study arms:
  - - Experimental arm:
      - XAV-19 at 0.5 mg/kg (Group 1) or at 2 mg/kg (Group 2)
    - Control Arm: Placebo
- **Treatment administration**
- During and following treatment infusion, the patient will be monitored every 30 minutes up to 2 hours after the infusion for signs or symptoms of any infusion reactions, including vital signs: heart rate, temperature, blood pressure, respiratory rate, SpO2, consciousness, nausea, fatigue, headache, malaise, myalgias, itching, rash.

**After XAV-19 infusion:**

- Blood sample immediately after end of infusion:
  - - Pharmacokinetics analysis
    - Spike/ACE2 interaction neutralizing antibody

### Visits on Day 2, Day 4, Day 6, Day 7, Day 11 and Day 13

- Documentation of any changes in clinical examination
- Vital signs
- Documentation of respiratory status: respiratory rate, oxygen supplementation, SpO2 at rest or PaO2, radiographic findings (if available)
- 7-point Ordinal scale
- Document concomitant medications
- Record any serious adverse events and all adverse events

Patients will be hospitalized at least from Day 1 until Day 3.

If patients are discharged from hospital before Day 8, visits on Day 4, Day 6, Day 7, Day 11 and Day 13 will be done via phone through directed questionnaire calls to evaluate the clinical status, collect any adverse event and any new concomitant medications.

### Visit on Day 3

- Documentation of any changes in clinical examination
- Vital signs
- Documentation of respiratory status: respiratory rate, oxygen supplementation, SpO2 at rest or PaO2, radiographic findings (if available)
- 7-point Ordinal scale
- Document concomitant medications
- Record any serious adverse events and all adverse events
- Blood analysis and urinary stick
- Blood sample collection:
  - - Pharmacokinetic sample
    - Spike/ACE2 interaction neutralizing antibody sample
    - Lymphocytes subpopulation
    - Cytokines
    - Transcriptomic analyses

### Visit on Day 5

**Before XAV-19 administration (if treatment administered otherwise at any time)**

- Documentation of any changes in clinical examination
- Vital signs
- Documentation of respiratory status: respiratory rate, oxygen supplementation, SpO2 at rest or PaO2, radiographic findings (if available)
- 7-point Ordinal scale
- Document concomitant medications
- Record any serious adverse events and all adverse events
- Blood analysis and urinary stick
- Blood sample collection immediately before the administration of XAV-19:
  - - Pharmacokinetic sample
    - Spike/ACE2 interaction neutralizing antibody sample
    - Lymphocytes subpopulation
    - Cytokines
    - Transcriptomic analyses

Following these procedures and only to be done if treatment administered at Day5:

- **Treatment administration (if applicable)**
- During and following treatment infusion, the patient will be monitored every 30 minutes up to 2 hours after the infusion for signs or symptoms of any infusion reactions, including vital signs: heart rate, temperature, blood pressure, respiratory rate, SpO2, consciousness, nausea, fatigue, headache, malaise, myalgias, itching, rash.

**After XAV-19 administration (only to be collected if treatment has been administered)**

- Blood sample immediately after end of infusion :
  - - Pharmacokinetics analysis
    - Spike/ACE2 interaction neutralizing antibody: and immediately after end of infusion

If patients are discharged from hospital before Day 5, patient will have to come back on-site for the Day 5 visit.

### Visits on Day 8, Day 15

- Documentation of any changes in clinical examination
- Vital signs
- Nasopharyngeal swab collection for RT-PCR SARS-Cov2 (qualitative assessment and viral load)
- Documentation of respiratory status: respiratory rate, oxygen supplementation, SpO2 at rest or PaO2, radiographic findings (if available)
- 7-point Ordinal scale
- Blood analysis and urinary stick
- Blood sample collection :
  - - Pharmacokinetic sample
    - Spike/ACE2 interaction neutralizing antibody sample
    - Lymphocytes subpopulation
    - Cytokines
    - Transcriptomic analyses
- Document concomitant medications
- Record any serious adverse events and all adverse events

If patients are discharged from hospital before Day 8, patient will have to come back on-site for the Day 8 and Day 15 visits.

### Visit on Day 29 / End of Study Visit

- Documentation of any changes in clinical examination
- Vital signs
- Nasopharyngeal swab collection for RT-PCR SARS-Cov2 (qualitative assessment and viral load)
- Documentation of respiratory status: respiratory Rate oxygen supplementation, SpO2 at rest or PaO2, radiographic findings (if available)
- 7-point Ordinal scale
- Blood analysis and urinary stick
- Blood sample collection:
  - - Pharmacokinetic sample
    - Spike/ACE2 interaction neutralizing antibody sample
- Document concomitant medications
- Record any serious adverse events and all adverse events

If patients are discharged from hospitalized before Day 29, patient will have to come back on-site for the Day 29 visit.

### Follow-up phone call at Day 60 (M2)

- Evaluation of the clinical status
- Document concomitant medications
- Record any serious adverse events and all adverse events

### Early Withdrawal Visit

- Documentation of any changes in clinical examination
- Vital signs
- Nasopharyngeal swab collection for RT-PCR SARS-Cov2 (qualitative assessment and viral load)
- Documentation of respiratory status: respiratory rate, oxygen supplementation, SpO2 at rest or PaO2, radiographic findings (if available)
- 7-point Ordinal scale
- Blood analysis and urinary stick
- Blood sample collection
- Document concomitant medications
- Record any serious adverse events and all adverse events

**STUDY SCHEDULE FOR all patients at dose 0.5mg/kg and the first two patients at dose 2mg/kg**

**in Phase 2a**

| **Activities** | 0 to 2 Days before inclusion (Screening) | D1  (Inclusion visit) | D2 | D3 | D4 | D5 | D6 | D7  ** | D8 | D11** | D13** | D15 | D29 | D60** | Early Withdrawal |
| --- | --- | --- | --- | --- | --- | --- | --- | --- | --- | --- | --- | --- | --- | --- | --- |
| Patient information | X |  |  |  |  |  |  |  |  |  |  |  |  |  |  |
| Informed Consent | X |  |  |  |  |  |  |  |  |  |  |  |  |  |  |
| Randomisation |  | X |  |  |  |  |  |  |  |  |  |  |  |  |  |
| Previous medical history | X |  |  |  |  |  |  |  |  |  |  |  |  |  |  |
| Clinical examination and vital signs^1^ | X | X | X | X | X | X | X | X | X | X | X | X | X | X | X |
| Respiratory status^2^ | X | X | X | X | X | X | X | X | X | X | X | X | X |  | X |
| XAV-19 administration |  | X |  |  |  | X |  |  |  |  |  |  |  |  |  |
| RT-PCR SARS-Cov2 (Nasopharyngeal swab samples) | * | X^3^ |  |  |  |  |  |  | X |  |  | X | X |  | X |
| ECG |  | X | X^4^ | X^4^ | X^4^ | X^4^ | X^4^ | X^4^ | X^4^ | X^4^ | X^4^ | X^4^ | X^4^ |  | X^4^ |
| Serology^5^ | X |  |  |  |  |  |  |  |  |  |  |  |  |  |  |
| Urine pregnancy test | X |  |  |  |  |  |  |  |  |  |  |  |  |  |  |
| Blood analysis and urinary stick^6^ | X | X |  | X |  | X |  |  | X |  |  | X | X |  | X |
| Pharmacokinetic^7^ |  | X^8^ |  | X |  | X^8^ |  |  | X |  |  | X | X |  |  |
| Transcriptomic analyses^7^ |  | X |  | X |  | X |  |  | X |  |  | X |  |  |  |
| Lymphocyte subpopulation^7^ |  | X |  | X |  | X |  |  | X |  |  | X |  |  |  |
| Cytokines^7^ |  | X |  | X |  | X |  |  | X |  |  | X |  |  |  |
| Spike/ACE2 neutralizing antibody titers^7^ |  | X^8^ |  | X |  | X^8^ |  |  | X |  |  | X | X |  |  |
| 7-point ordinal scale | X | X | X | X | X | X | X | X | X | X | X | X | X |  | X |
| Concomitant treatment | X | X | X | X | X | X | X | X | X | X | X | X | X | X | X |
| Adverse events | X | X | X | X | X | X | X | X | X | X | X | X | X | X | X |

^*^ Documented positive SARS-CoV-2 RT PCR within past 10 days.

**: If patients are discharged from hospital as early as Day 6, visits on Day 7, Day 11 and Day 13 will be done via phone calls to evaluate the clinical status, collect any adverse event and any new concomitant medications. Day 60 is a phone call visit.

^1^: Clinical examination: Full physical examination at Day1 and brief targeted examination at other visits. Vital signs: Blood pressure, pulse rate, body temperature, Height (cm), (only at D1), and body weight (kg). To be performed between 8 and 12 am.

^2^.Respiratory status: respiratory rate, oxygen supplementation, SpO2 at rest or PaO2, radiographic findings (if available)

^3^:For viral load assessment. Not to be done if screening PCR was obtained in the same study site

^4^: ECG: to be performed at the discretion of the Investigator, as deemed necessary for appropriate patient care

^5^: Serology: HIV, hepatitis B/C

^6^: Blood analysis: **Hematology**: hemoglobin, hematocrit, red blood cell (RBC) count, white blood cell (WBC) count with differential (absolute counts and including calculation of total lymphocytes), and platelet (PLT) count.

**Serum Chemistry:** creatinine, fasting glucose, total protein, phosphorus, bicarbonate, total bilirubin, direct and indirect bilirubin, alkaline phosphatase (ALP), aspartate aminotransferase (AST), alanine aminotransferase (ALT), gamma glutamyltransferase (GGT), sodium (Na), potassium (K), chloride (Cl), albumin, C-reactive protein, ferritin, D-dimers, IL-6 and procalcitonin (if available locally). Creatine kinase (CK), lactate dehydrogenase (LDH), lipase

**Urinary stick: if positive** urinary stick, proteinuria analysis to be performed

^7^ : phase 2a : all patients, phase 2b: only for patients participating in the ancillary studies

^8^: immediately before administration and at the end of infusion

**STUDY SCHEDULE FOR the last 6 patients at dose 2mg/kg in Phase 2a**

| **Activities** | 0 to 2 Days before inclusion (Screening) | D1  (Inclusion visit) | D2 | D3 | D4  ** | D5 | D6  ** | D7  ** | D8 | D11** | D13** | D15 | D29 | D60** | Early Withdrawal |
| --- | --- | --- | --- | --- | --- | --- | --- | --- | --- | --- | --- | --- | --- | --- | --- |
| Patient information | X |  |  |  |  |  |  |  |  |  |  |  |  |  |  |
| Informed Consent | X |  |  |  |  |  |  |  |  |  |  |  |  |  |  |
| Randomisation |  | X |  |  |  |  |  |  |  |  |  |  |  |  |  |
| Previous medical history | X |  |  |  |  |  |  |  |  |  |  |  |  |  |  |
| Clinical examination and vital signs^1^ | X | X | X | X | X** | X | X** | X** | X | X** | X** | X | X | X | X |
| Respiratory status^2^ | X | X | X | X | X*** | X | X*** | X*** | X | X*** | X*** | X | X |  | X |
| XAV-19 administration |  | X |  |  |  |  |  |  |  |  |  |  |  |  |  |
| RT-PCR SARS-Cov2 (Nasopharyngeal swab samples) | * | X^3^ |  |  |  |  |  |  | X |  |  | X | X |  | X |
| ECG |  | X | X^4^ | X^4^ | X^4^ | X^4^ | X^4^ | X^4^ | X^4^ | X^4^ | X^4^ | X^4^ | X^4^ |  | X^4^ |
| Serology^5^ | X |  |  |  |  |  |  |  |  |  |  |  |  |  |  |
| Urine pregnancy test | X |  |  |  |  |  |  |  |  |  |  |  |  |  |  |
| Blood analysis and urinary stick^6^ | X | X |  | X |  | X |  |  | X |  |  | X | X |  | X |
| Pharmacokinetic^7^ |  | X^8^ |  | X |  | X |  |  | X |  |  | X | X |  |  |
| Transcriptomic analyses^7^ |  | X |  | X |  | X |  |  | X |  |  | X |  |  |  |
| Lymphocyte subpopulation^7^ |  | X |  | X |  | X |  |  | X |  |  | X |  |  |  |
| Cytokines^7^ |  | X |  | X |  | X |  |  | X |  |  | X |  |  |  |
| Spike/ACE2 neutralizing antibody titers^7^ |  | X^8^ |  | X |  | X^9^ |  |  | X |  |  | X | X |  |  |
| 7-point ordinal scale | X | X | X | X | X | X | X | X | X | X | X | X | X |  | X |
| Concomitant treatment | X | X | X | X | X | X | X | X | X | X | X | X | X | X | X |
| Adverse events | X | X | X | X | X | X | X | X | X | X | X | X | X | X | X |

^*^ Documented positive SARS-CoV-2 RT PCR within past 10 days.

**: If patients are discharged from hospital as early as Day 3, visits on Day 4, Day 6, Day 7, Day 11 and Day 13 will be done via phone calls to evaluate the clinical status, collect any adverse event and any new concomitant medications. Day 60 is a phone call visit.

^1^: Clinical examination: Full physical examination at Day1 and brief targeted examination at other visits. Vital signs: Blood pressure, pulse rate, body temperature, Height (cm), (only at D1), and body weight (kg). To be performed between 8 and 12 am. If patients are discharged before Day 8 (as early as Day 3), phone visits on Day 4, Day 6, Day 7, Day 11 and Day 13 will collect respiratory and general status through directed questionnaire.

^2^.Respiratory status (*** only for hospitalized patients): respiratory rate, oxygen supplementation, SpO2 at rest or PaO2, radiographic findings (if available)

^3^: For viral load assessment. Not to be done if screening PCR was obtained in the same study site

^4^: ECG: to be performed at the discretion of the Investigator, as deemed necessary for appropriate patient care

^5^: Serology: HIV, hepatitis B/C

^6^: Blood analysis: **Hematology**: hemoglobin, hematocrit, red blood cell (RBC) count, white blood cell (WBC) count with differential (absolute counts and including calculation of total lymphocytes), and platelet (PLT) count.

**Serum Chemistry:** creatinine, fasting glucose, total protein, phosphorus, bicarbonate, total bilirubin, direct and indirect bilirubin, alkaline phosphatase (ALP), aspartate aminotransferase (AST), alanine aminotransferase (ALT), gamma glutamyltransferase (GGT), sodium (Na), potassium (K), chloride (Cl), albumin , C-reactive protein, ferritin, D-dimers, IL-6 and procalcitonin (if available locally). Creatine kinase (CK), lactate dehydrogenase (LDH), lipase

**Urinary stick: if positive** urinary stick, proteinuria analysis to be performed

^7^: phase 2a: all patients,

^8^: immediately before administration and at the end of infusion

^9^ : at the time of blood analysis (same venous sampling)

## Study schedule for phase 2B

The study schedule is summarized in the flowchart below; all of these examinations apply to all patients from phase 2b, the following blood collections are part of ancillary studies as follows:

Ancillary study 1: 90 patients from Phase 2b

- - - Pharmacokinetic Study

Ancillary study 2: 100 patients from Phase 2b

- - - Spike/ACE2 interaction neutralizing antibody sample
    - Lymphocytes subpopulation
    - Cytokines
    - Transcriptomic analyses

Terminal ancillary study 3: 20 patients in addition to phase 2 b. The blood samples in this study are equivalent to those in the first and second ancillary pharmacokinetic study.

### Screening Visit (Day –2 / Day 1)

Subjects will be screened within 2 days before randomization and dosing to determine eligibility for participation in the study. The same applies to the terminal ancillary study. Practically, Day 1 is the day following Day-1, so patients can be randomized either one or two days after, or the same day as screening. Following presentation of the study and the informed consent form by the investigator research team, participants will have enough time to take their decision regarding their participation.

- Obtain written informed consent. This should be documented in the medical file of the patient. After the informed consent, the following assessments are performed to determine eligibility requirements as specified in the inclusion and exclusion criteria:
- Focused medical history including the following information (eg, date of first symptoms, overall symptoms, exposure source, demographics, baseline characteristics, allergies and medical history)
- Review and record medications and therapies for this current illness
- Counsel subjects to use adequate birth control methods required during the trial to avoid pregnancy
- Targeted physical examination including, vital signs (heart rate, temperature, blood pressure, level of consciousness), body weight, and height
- Documentation of respiratory status: respiratory rate, oxygen supplementation, SpO2 at rest or PaO2, radiographic findings
- Obtain blood samples if not done in the preceding 48 hours for white blood cell count, hematocrit, platelets, creatinine and creatinine clearance, glucose, total bilirubin, ALT, AST
- Urinary Pregnancy test (for women of childbearing potential)
- Administration of the 8-point Ordinal scale
- Record any serious adverse events and all adverse events related to protocol-mandated procedures occurring after signing of the consent form.

Study subjects who qualify should be immediately randomized, which could occur on same day as screening (Day 1). Randomization and dosing should occur on the same day if possible.

### Inclusion Visit (Day 1)

The following evaluations are to be completed at the Day 1 visit.

The investigator must have confirmed eligibility before proceeding with randomization on the Day 1 visit.

If the screening and Day 1 visits occur within 24 hours, blood analysis do not need to be repeated.

Investigators must complete the following assessments before administration of study drug:

**Before XAV-19 infusion**

- Full clinical examination
- Vital signs
- ECG
- Documentation of respiratory status: respiratory rate, oxygen supplementation, SpO2 at rest or PaO2, radiographic findings (if available)
- Nasopharyngeal swab collection for RT-PCR SARS-CoV-2 (unless diagnosis PCR was done at the same hospital). After analysis, this sample will be stored at local site.
- 8-point ordinal scale
- Blood analysis (unless screening and inclusion are the same day)
- Blood sample collection before the administration of XAV-19 (if applicable):
  - - Pharmacokinetic samples (for the first 20 patients included in this ancillary study, for following patients the PK analysis will be done from immunology blood samples)
    - Spike/ACE2 interaction neutralizing antibody sample
    - Lymphocytes subpopulation
    - Cytokines
    - Transcriptomic analyses
- Document concomitant medications
- Record any serious adverse events and all adverse events

Following these procedures:

- Randomisation to one of the two study arms:
  - - Experimental arm: Xav-19 infusion over one hour at 2mg/kg
    - Control Arm: Placebo
- **Treatment administration**
  - Patients will be monitored for signs or symptoms of any infusion reactions and vital signs will be monitored twice: at the beginning and at the end of the treatment administration

For the ancillary terminal study, the only difference is that there will be no randomization, patients will all receive 150 mg of XAV-19.

**After XAV-19 infusion, only for patients participating to the ancillary studies:**

- Blood sample immediately after end of infusion:
  - - Pharmacokinetics analysis (for the first 20 patients included in this ancillary study, for following patients the PK analysis will be done from immunology blood samples ; and for the 20 patients of the terminal ancillary study)
    - Spike/ACE2 interaction neutralizing antibody (for patients included in the ancillary study 2 and for the 20 patients of the terminal ancillary study)

Patients will be hospitalized at least from Day 1 until Day 3.

### Visit on Day 2

- Documentation of any changes in clinical examination
- Vital signs
- Documentation of respiratory status: respiratory rate, oxygen supplementation, SpO2 at rest or PaO2, radiographic findings (if available)
- 8-point Ordinal scale
- Document concomitant medications
- Record any serious adverse events and all adverse events

### Visit on Day 3

- Documentation of any changes in clinical examination
- Vital signs
- Documentation of respiratory status: respiratory rate, oxygen supplementation, SpO2 at rest or PaO2, radiographic findings (if available)
- 8-point Ordinal scale
- Document concomitant medications
- Record any serious adverse events and all adverse events
- Blood analysis
- Blood sample collection (if applicable):
  - - Pharmacokinetic sample (for the first 20 patients included in this ancillary study, for following patients the PK analysis will be done from immunology blood samples ; and for the 20 patients of the terminal ancillary study)
    - Spike/ACE2 interaction neutralizing antibody sample
    - Lymphocytes subpopulation
    - Cytokines
    - Transcriptomic analyses

### Visit on Day 5 (if still hospitalized)

- Documentation of any changes in clinical examination
- Vital signs
- Documentation of respiratory status: respiratory rate, oxygen supplementation, SpO2 at rest or PaO2, radiographic findings (if available)
- 8-point Ordinal scale
- Document concomitant medications
- Record any serious adverse events and all adverse events
- Blood analysis
- Blood sample collection (if applicable):
  - - Pharmacokinetic sample
    - Spike/ACE2 interaction neutralizing antibody sample
    - Lymphocytes subpopulation
    - Cytokines
    - Transcriptomic analyses

If patients are discharged from hospital before Day 5, patient will not come on-site for the Day 5 visit, this will be done via a phone call, except if patient is participating to the ancillary studies, in which case patient will have to come on site for this visit.

### Visits on Day 8, Day 15

- Documentation of any changes in clinical examination
- Vital signs
- Nasopharyngeal swab collection for RT-PCR SARS-Cov2 (qualitative assessment and viral load), after qualitative analysis, this sample will be stored at local site.
- Documentation of respiratory status: respiratory rate, oxygen supplementation, SpO2 at rest or PaO2, radiographic findings (if available)
- 8-point Ordinal scale
- Blood analysis
- Blood sample collection (if applicable):
  - - Pharmacokinetic sample (for the first 20 patients included in this ancillary study, for following patients the PK analysis will be done from immunology blood samples ; and for the 20 patients of the terminal ancillary study)
    - Spike/ACE2 interaction neutralizing antibody sample
    - Lymphocytes subpopulation
    - Cytokines
    - Transcriptomic analyses
- Document concomitant medications
- Record any serious adverse events and all adverse events

If patients are discharged from hospital before Day 8, patient will have to come back on-site for the Day 8 and Day 15 visits.

### Visit on Day 29 / End of Study Visit

- Documentation of any changes in clinical examination
- Vital signs done only if visit on site
- Nasopharyngeal swab collection for RT-PCR SARS-Cov2 (qualitative assessment and viral load) done only if visit on site, after qualitative analysis, this sample will be stored at local site.
- Documentation of respiratory status: respiratory Rate oxygen supplementation, SpO2 at rest or PaO2, radiographic findings (if available)
- 8-point Ordinal scale
- Blood analysis done only if visit on site
- Blood sample collection (if applicable):
  - - Pharmacokinetic sample (for the first 20 patients included in this ancillary study, for following patients the PK analysis will be done from immunology blood samples ; and for the 20 patients of the terminal ancillary study)
    - Spike/ACE2 interaction neutralizing antibody sample
- Document concomitant medications
- Record any serious adverse events and all adverse events

If patients are discharged from hospital before Day 29, patient will not come on-site for the Day 29 visit, this will be done via a phone call, except if patient is participating to the ancillary studies, in which case patient will come on site for this visit.

### Follow-up phone call at Day 60 (M2)

- Evaluation of the clinical status
- Document concomitant medications
- Record any serious adverse events and all adverse events

### Early Withdrawal Visit

- Documentation of any changes in clinical examination
- Vital signs if before day 15
- Nasopharyngeal swab collection for RT-PCR SARS-Cov2 (qualitative assessment and viral load) if before day 15, after qualitative analysis, this sample will be stored at local site.
- Documentation of respiratory status: respiratory rate, oxygen supplementation, SpO2 at rest or PaO2, radiographic findings (if available)
- 8-point Ordinal scale
- Blood analysis if before day 15
- Blood sample collection (if applicable)
- Document concomitant medications
- Record any serious adverse events and all adverse events

**STUDY SCHEDULE FOR all patients in Phase 2b**

| **Activities** | 0 to 2 Days before inclusion (Screening) | D1  (Inclusion visit) | D2 | D3 | D5 **  (+/- 1 day) | D8  (+/- 1 day) | D15  (+/- 1 day) | D29  ***  (+/- 1 day) | D60  **** | Early Withdrawal |
| --- | --- | --- | --- | --- | --- | --- | --- | --- | --- | --- |
| Patient information | X |  |  |  |  |  |  |  |  |  |
| Informed Consent | X |  |  |  |  |  |  |  |  |  |
| Randomisation |  | X |  |  |  |  |  |  |  |  |
| Previous medical history | X |  |  |  |  |  |  |  |  |  |
| Clinical examination and vital signs^1^ | X | X | X | X | X | X | X | X | X | X^9:^ |
| Respiratory status^2^ | X | X | X | X | X | X | X | X |  | X |
| XAV-19 administration |  | X |  |  |  |  |  |  |  |  |
| Diagnosis of SARS-Cov-2 infection before screening, by positive SARS-CoV-2 RT-PCR or antigen (*) | X |  |  |  |  |  |  |  |  |  |
| RT-PCR SARS-Cov2 (Nasopharyngeal swab samples) | * | X^3^ |  |  |  | X^11^ | X^11^ | X^11^ |  | X^11^, ^9:^ |
| ECG |  | X | X^4^ | X^4^ | X^4^ | X^4^ | X^4^ | X^4^ |  | X^4^ |
| Serology^5^ | X |  |  |  |  |  |  |  |  |  |
| Urine pregnancy test | X |  |  |  |  |  |  |  |  |  |
| Blood analysis^6^ | X | X |  | X | X | X | X | X |  | X^9:^ |
| Pharmacokinetic^7^ |  | X^8^ |  | X | X | X | X | X |  |  |
| Transcriptomic analyses^7^ |  | X |  | X | X | X | X |  |  |  |
| Lymphocyte subpopulation^7^ |  | X |  | X | X | X | X |  |  |  |
| Cytokines^7^ |  | X |  | X | X | X | X |  |  |  |
| Spike/ACE2 neutralizing antibody titers^7^ |  | X^8^ |  | X | X | X | X | X |  |  |
| 8-point ordinal scale^10^ | X | X | X | X | X | X | X | X |  | X |
| Concomitant treatment | X | X | X | X | X | X | X | X | X | X |
| Adverse events | X | X | X | X | X | X | X | X | X | X |

* Documented positive SARS-CoV-2 RT PCR before enrolment (if screening nasopharyngeal swab sample done at study site, this sample should be stored after analysis).

**: Only if still hospitalized at Day 5, otherwise phone all. Patients participating to the ancillary studies will have to come back on site for this visit.

*** If not hospitalized Day 29 is a phone call visit except for patients participating to the ancillary studies will have to come back on site for this visit (Vital signs, Nasopharyngeal swab collection for RT-PCR SARS-Cov2 and blood analysis will be done only if visit on site).

**** if not hospitalized Day 60 is a phone call visit.

^1^: Clinical examination: Full physical examination at Day1 and brief targeted examination at other visits. Vital signs: Blood pressure, pulse rate, body temperature, Height (cm) and body weight (kg) (only at D1),.

^2^.Respiratory status: respiratory rate, oxygen supplementation, SpO2 at rest or PaO2, radiographic findings (if available). For outpatients, only supplement oxygenation will be enquired.

^3^: For viral load assessment. Not to be done if screening RT-PCR was obtained in the same study site. After qualitative analysis, nasopharyngeal swab samples to be stored at the site)

^4^: ECG: to be performed at the discretion of the Investigator, as deemed necessary for appropriate patient care

^5^: Serology: HIV, hepatitis B/C, COVID-19 (after analysis remaining serum will be stored under usual conditions at local site)

^6^: Blood analysis: **Hematology**: hemoglobin, hematocrit, red blood cell (RBC) count, white blood cell (WBC) count with differential (absolute counts and including calculation of total lymphocytes), and platelet (PLT) count.

**Serum Chemistry:** creatinine, glucose, total protein, total bilirubin, alkaline phosphatase (ALP), aspartate aminotransferase (AST), alanine aminotransferase (ALT), gamma glutamyltransferase (GGT), C-reactive protein, ferritin,

^7^: Only for patients participating in the ancillary studies

^8^: Immediately before administration and at the end of infusion

^9:^ done only if before day 15

^10^: assessed daily

^11^: For viral load assessment. After qualitative analysis, nasopharyngeal swab samples to be stored at the site)

## Procedures details

All examinations are performed by the investigator or a qualified member of the investigational staff.

- All inclusion and exclusion criteria will be reviewed prior to patients entering the study

- Informed consent form will be signed prior to any specific study procedure.
- Medical History: including COPD and chronic respiratory insufficiency, and if applicable, assessment of their severity.
- The full physical examination includes the following assessments will be done: Cardiovascular, Head-Ear-nose-throat, Eyes, Gastro-intestinal, General Appearance, Lymph nodes, Musculoskeletal, Neurological, Respiratory, Skin and Mucous Membranes. Body weight and height will be measured.

Any abnormalities present at Inclusion, or subsequent changes, will be documented in the appropriate sections of the eCRF. Any clinically significant abnormalities persisting at the end of the study will be followed by the investigator until resolution or until reaching a clinically stable endpoint.

- Vital signs include: Blood pressure, pulse rate and body temperature will be taken at each assessment per the Study Schedule. Height (cm) and weight (kg) will be taken only at screening. Blood pressure and pulse will be taken after the patient has been resting in supine position for 5 minutes. Level of consciousness will also be assessed.
- Respiratory status: respiratory rate, oxygen supplementation, SpO2 at rest or PaO2, radiographic findings (if available).
- Electrocardiogram (ECG):

The 12-lead digitalized ECGs will be recorded according to the Study Schedule while the patient is in supine position for at least 5 minutes. ECG is performed at inclusion visit, other ECG will be performed at the discretion of the Investigator, as deemed necessary for appropriate patient care.

- Blood analyses and serology:

Blood samples for serum chemistry and haematology will be collected according to the Study Schedule. In addition, a urine sample for women of childbearing potential for pregnancy testing will be collected at screening to confirm eligibility criteria (in case as per local site practice pregnancy testing is done on blood sample, no additional urine sample is needed and the blood pregnancy testing result will be collected). The investigator must review the laboratory report, document this review, and record any clinically relevant changes occurring during the study in the adverse event section of the eCRF.

Results of serological tests performed prior to the subject signing consent as part of routine clinical management is acceptable in lieu of a screening test.

***Serum Chemistry***

- creatinine,
- urea*,
- glucose,
- total protein,
- phosphorus*,
- bicarbonate*,
- total bilirubin,
- direct and indirect bilirubin*,
- alkaline phosphatase (ALP),
- aspartate aminotransferase (AST),
- alanine aminotransferase (ALT),
- gamma glutamyltransferase (GGT),
- sodium (Na)*,
- potassium (K)*,
- chloride (Cl)*,
- albumin*,
- creatine kinase (CK)*,
- lactate deshydrogenase (LDH)*,
- lipase*

***Haematology:***

- hemoglobin,
- hematocrit,
- red blood cell (RBC) count,
- white blood cell (WBC) count with differential (absolute counts and including calculation of total lymphocytes),
- platelet (PLT) count

***Serology (remaining serum after analysis will be stored under usual conditions at local site):***

- HIV,
- hepatitis (B & C),
- COVID-19

***Inflammatory markers and cytokines***

- C-reactive protein (CRP)
- Ferritin
- D-dimers*
- IL-6* (if available locally)
- Procalcitonin* (if available locally)

***Urine analysis (by stick)*** ****:***

- Proteinuria

* Only for Phase 2a

All of these blood and urine analysis will be performed locally by each center.

- Pharmacokinetic analysis:

Blood sample (3,5mL) will be collected at each draw for specific analysis as described below according to the Study Schedule

These analyses will be performed a R&D grade locally in order to provide data to the DSMC. Final data will be obtained from Citoxlab/Charles Rivers and run under GLP conditions.

In the phase 2a all patients included in the study will have pharmacokinetic samples taken.

- Immunological analyses on blood sample:

Blood sample will be collected for specific analysis as described below according to the Study Schedule. Actual time and date of blood drawn must be accurately recorded on the eCRF.

The following analyses will be performed:

-T and B lymphocyte subpopulations

-Spike/ACE2 interaction neutralizing antibody.

-Serum cytokines: MCP1, IP10, IL-10, IFNa, TNF-α, IL-1b, IL-6, IL-82 (in the phase 2a) and IL-6, IFNa2, IL-10, IP10, CCL2 (in the phase 2b).

These analyses will be performed by the CIMNA/Nantes University Hospital.

- Transcriptomic analyses

Messenger RNA molecules will be extracted from peripheral blood in order to identify transcripts that are specifically over- or under-expressed in correlation with the clinical status and with viral load. The goal is to identify possible biomarkers of the disease severity and disease responding to the treatment, and, using deconvolution bioanalyses, also identifying cell population changes in complement to the direct flow cytometry analyses. A focus will be made on B cell subpopulation and their role in interaction with Tfh cells.

These immunological analyses will be performed by the CRTI in Nantes University (INSERM U1064) (lead by Sophie Brouard, CNRS).

During the study, including the end-of-study visit, the total blood volume to be collected from each patient specific for the research analyses will be 253mL (1).

These estimates are for required additional blood samples specific for the study and do not include additional blood samples that may be collected at the investigator’s discretion for drug level monitoring or other clinical laboratory tests deemed necessary for appropriate patient care in case of occurrence of adverse events.

| Table 1: Estimated Blood Volume Drawn specific to the study: | | | |
| --- | --- | --- | --- |
| **Type of Sample** | **Volume per Sample (mL)** | **No. of Samples per Patient** | **Total Volume of Blood  (mL)^a^** |
|  |  |  |  |
| PK  Lymphocyte sub-population and cytokine | 3,5  5 | 8 (7)  5 | 28 (24.5)  25 |
| Spike/ACE2 neutralizing antibody titers | 5 | 8 (7) | 40 (35) |
| Transcriptomic | 50 | 5 | 200 |
| Total blood volume drawn |  |  | 293 (284.5) |
| ^a^ Calculated as number of samples multiplied by amount of blood per sample.  (x) If only one treatment administration at D1 | | | |

- Nasopharyngeal swab samples will be used to perform RT-PCR in order to detect SARS-CoV-2, and collect information (D1 or screening sample) on presence or not of variant. These samples should be stored after analysis at the site for further analysis at central laboratory (viral load).
- Ordinal scale:

The ordinal scale is an assessment administered by the investigator to determine the clinical status of the patient, the scale is as follows:

The 7-point ordinal scale (Phase 2a)

1. Not hospitalized, no limitations on activities;

2. Not hospitalized, limitations on activities;

3. Hospitalized, not requiring supplemental oxygen;

4. Hospitalized, requiring supplemental oxygen;

5. Hospitalized, on non-invasive ventilation or high flow O2 devices;

6. Hospitalized, on invasive mechanical ventilation or ECMO;

7. Death.

The 8-point ordinal scale (<https://www.who.int/blueprint/priority-diseases/key-action/COVID-19_Treatment_Trial_Design_Master_Protocol_synopsis_Final_18022020.pdf>) (Phase 2b)


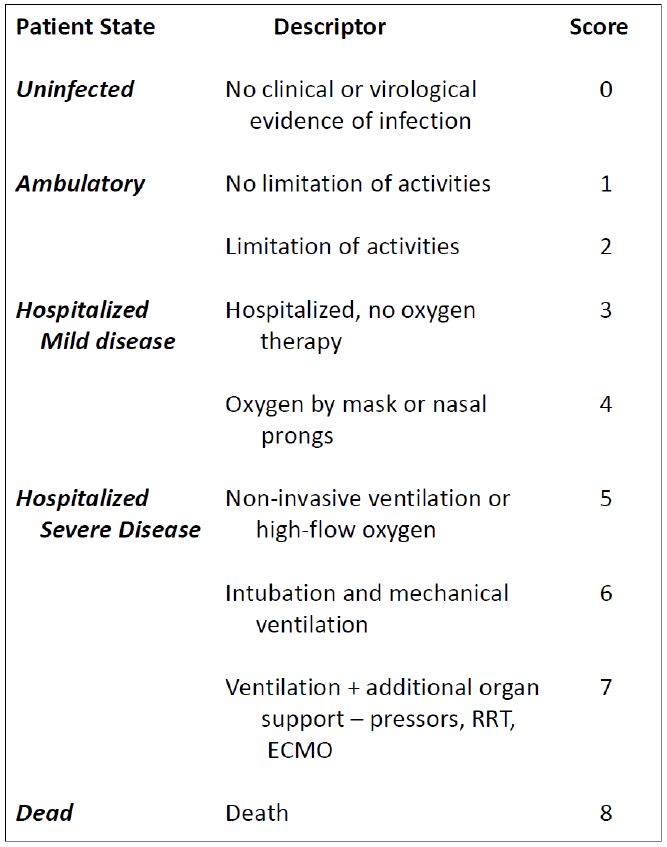


The ordinal scale of the clinical status will be assessed daily until Day29. Each day, the worse score for the previous 24 hours will be recorded. i.e. on day 3, day 2 score is obtained and recorded as day 2.

On Day 1, assessment will have to be performed before IMP infusion.

- National Early Warning Score (NewS2) (<https://www.cebm.net/covid-19/should-we-use-the-news-or-news2-score-when-assessing-patients-with-possible-covid-19-in-primary-care/>):

The NEWS is based on a simple aggregate scoring system in which a score is allocated to physiological measurements. Six simple physiological parameters form the basis of the scoring system:

- respiration rate
- oxygen saturation
- systolic blood pressure
- pulse rate
- level of consciousness or new confusion
- temperature.

The score is then aggregated and uplifted by 2 points for people requiring supplemental oxygen to maintain their recommended oxygen saturation.


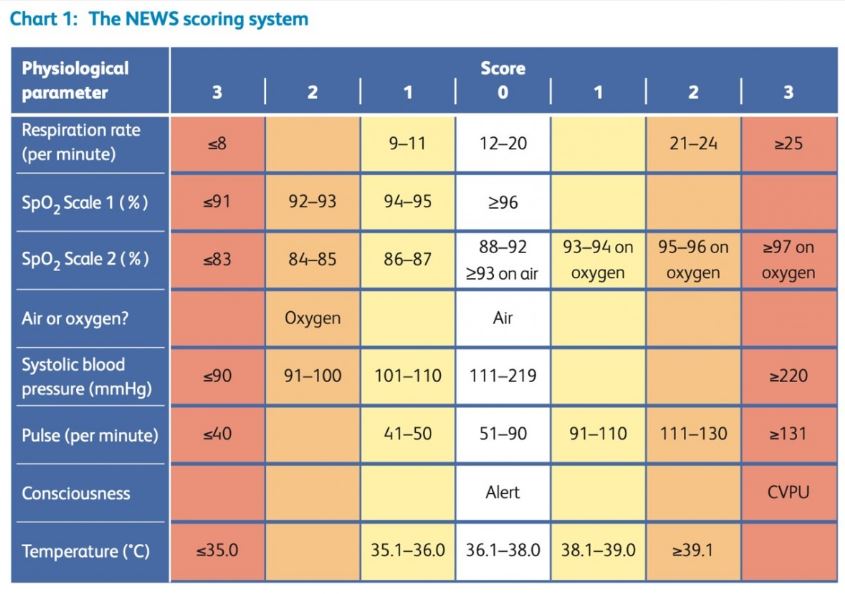


PaO2/FiO2 Ratio (EPICII conversion table8 May 2007)
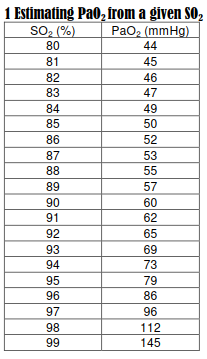


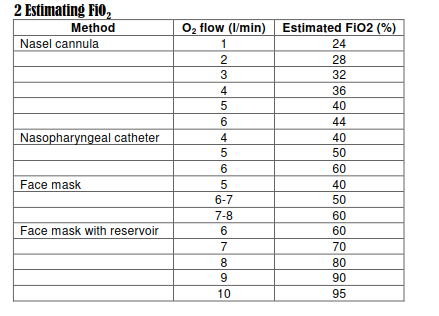


- Concomitant treatments

Concomitant treatments will be reported for the duration of the study. Concomitant treatments will be reported in the eCRF

- Adverse Events

Adverse events will be reported for the duration of the study from the signature of the informed consent form until the end of the study. Adverse events will be followed by the investigator as specified in Section 8, and reported in the eCRF.

## General study methodology

The study presents the following characteristics:

- ***Phase 2a and 2b study to evaluate the safety and efficacy of XAV-19***
- ***Both phases are multicenter, placebo-controlled, double-blind, randomized***
- ***Phase 2a is a sequential 2 groups ascending dose, with a 1:1 randomisation for the first two patients and a 5:1 ratio (treatment: placebo) for the 6 following patients). Subjects will be dosed with a minimal interval of 24h (first to first administration) all sites combined.***
- ***Phase 2b is a double-blinded 1:1 randomised study with randomisation balanced by blocks. A randomisation by center and by duration between onset of COVID-19 symptoms and screening (1-6 days, 7-10, 11 to 14 days) will be settled****.*
- To note, the terminal ancillary study of phase 2b will be performed as open label.

## Study diagram

### Phase 2a – Group1

Adults patients with moderate COVID-19 confirmed by RT-PCR

Inclusion visit: verification of eligibility criteria and signature of informed consent form

Follow-up visits until day 29 and phone call at Day 60

**Experimental Group 1**

XAV-19 at 0,5mg/kg

Administration at Day 1 and Day 5

6 patients

Randomisation 5:1 (treatment : placebo)

2 patients

Randomisation 1:1 (treatment : placebo)

Stop

1st XAV-19 Administration Day1

1st XAV-19 Administration Day1

**Safety Data Review**

**&**

**Evaluation by DSMC**

2nd XAV-19 Administration Day5

2nd XAV-19 Administration Day5

Follow-up Visits until Day29 and phone call at Day 60

Follow-up Visits until Day29

and phone call at Day 60

**DSMC**

**Evaluation of data until Day 8 for the 8 patients**

**GROUP2**

**STOP**

Stop

### Phase 2a - Group2

Adults patients with moderate COVID-19 confirmed by RT-PCR

Follow-up visits until day 29 and phone call at Day 60

Inclusion visit: verification of eligibility criteria and signature of informed consent form

**Experimental Group 2**

XAV-19 at 2mg/kg

Administration at Day 1 and Day 5

6 patients

Randomisation 5:1 (treatment : placebo)

2 patients

Randomisation 1:1 (treatment : placebo)

Stop

1st XAV-19 Administration Day1

1st XAV-19 Administration Day1

**Safety Data Review**

**&**

**Evaluation by DSMC**

2nd XAV-19

Administration Day5

Follow-up Visits until Day29 and phone call at Day 60

Follow-up Visits until Day29

and phone call at Day 60

**Phase 2b**

**STOP**

Stop

**DSMC**

**Evaluation of data until Day 8 for the 8 patients**

### Phase 2b

398 Adults patients with moderate COVID-19 confirmed by RT-PCR + 20 patients (terminal ancillary study)

D0: Inclusion visit: verification of eligibility criteria and signature of informed consent form

Follow-up visits until day 29 and phone call at Day 60

D1: Randomisation 1:1 (treatment:placebo) only for the first 398 patients

1st XAV-19 Administration Day1

(fixed dose of 150mg for the 20 patients of the terminal ancillary study)

Follow-up visits until Day 29 and phone call at Day 60

Follow-up visits until Day 29

Follow-up visits until Day 29

Follow-up visits until Day 29

## Identification of all data sources not included in the medical record

All specific data from the study will be compiled directly in the eCRF, as they will not be found in medical record.

These specific data are:

- Urinary pregnancy test at D1

- 12-Lead ECG at D1

- Immunological analysis

- Spike/ACE2 interaction neutralizing antibody
- Cytokines
- Lymphocytes sub-population
- Transcriptomic analyses

- PK analysis

- The 7-point Ordinal Scale or the 8-point Ordinal Scale

All other data will be present in the patient medical file.

## Rules for discontinuing subject participation

### Criteria in respect of early withdrawal of a subject from the study

Withdrawals from the study can only be effective after confirmation by the investigator and the sponsor. These withdrawals are always definitive

Patients can withdraw from participation in this study at any time, for any reason, specified or unspecified, and without prejudice.

Criteria in respect of early withdrawal of a subject from the study are the following:

- Withdrawal of informed consent (subject’s decision to withdraw for any reason). All data gathered before their withdrawal will be kept for analysis
- Investigator’s decision to stop the study in the patient’s best interest. Patients who stop prematurely or definitively the study treatment will be kept in the study for follow-up
- Subjects who have grade 4 reaction secondary to first XAV-19 administration and/or develop serum sickness disease, serious allergic reaction such as anaphylactic shock, severe hypotension, tachycardia or Quincke’s edema following first XAV-19 administration will discontinue study treatment but will be kept in the study to perform the required study-related follow-up and procedures
- For the phase 2a, subjects who discontinue prior to the Day 8 in phase 2a will be replaced, in order to obtain the required number of evaluable subjects in each group (8 patients). There will be no replacement of discontinued patients in phase 2b.
- Termination of the study by the Sponsor

Stopping rules:

| **Parameter** | **Value** |
| --- | --- |
| Adverse events | Grade 4 reaction secondary to first XAV-19 administration and/or develop serum sickness disease, serious allergic reaction such as anaphylactic shock, severe hypotension, tachycardia or Quincke’s edema |
| **Vital signs** |  |
| - Bradycardia | - <50 beats/min or ECG abnormalities |
| - Tachycardia | - >130 beats/min or ventricular dysrhythmias |
| Supine systolic blood pressure increase | >200 mmHg or headache or clinical signs |
| Supine diastolic blood pressure increase | >120 mmHg or headache or clinical signs |
| Supine systolic blood pressure decrease | <70 mmHg or symptomatic |

Every effort should be made to keep the patients in the trial, if possible, on treatment, or at least post-treatment discontinuation to collect important trial data.

For the data processing procedures in respect of subjects withdrawn early from the study, refer to the statistical section.

### Procedures in respect of early withdrawal of a subject from the study

In the event of early discontinuation of the study treatment, all efforts will be made by the investigator to invite the patient to undergo the end of study (EOS) visit.

The primary reason for discontinuation must be recorded in the appropriate section of the e-CRF and all efforts made to complete and report the observations as thoroughly as possible. A complete final evaluation following the patient's withdrawal should be made, as described in Section 6.1.8 (Early Withdrawal Visit).

If a patient fails to return for a scheduled visit/follow up, attempts should be made to contact the patient to identify reason for not returning. Likewise, if a patient declares his/her wish to discontinue from the study e.g., for personal reasons, all efforts should be made to establish that the cause is not due to an AE (bearing in mind the patient is not obliged to state his/her reasons).

For the data processing procedures in respect of subjects withdrawn early from the study, refer to the statistical section.

Each early departure from the study should receive the usual medical follow‐up.

### Criteria in respect of discontinuation of all or part of the study (excluding biostatistical considerations)

The first part of the study (phase 2a) as a first-in-human study is conducted with sequential enrolment to check for safety.

The DSMC will give recommendation regarding modification to the ongoing conduct of the trial (see 1.2.4) and give approval for continuing the study to the next step within each group of phase 2a and between the phase 2a and 2b.

At any time during the phase 2a study, if a serious adverse event occurs, this will be communicated to the DSMC, and the sponsor and coordinating investigator will have the responsibility to decide whether DSMC must meet to advise on study conduct. The DSMC may also be requested for a review by the person in charge of safety pharmacology if a SUSAR or a SAE presents a particular analytical problem or if a doubt in respect benefit/risk arises during the study and advise on study conduct.

Due to the small number of patients and the short duration of follow-up, no further rules for early termination of research are defined for phase 2a.

During the main phase of the study (phase 2b), the DSMC will regularly meets as defined in the DSMC charter and may decide to stop the study prematurely.

During the phase 2b study, if the proportion of patients with need of invasive mechanical ventilation and/or death after inclusion reaches to 20% or above (analyzed by incremental groups of 50 patients), the DSMC will be asked to review the data without stopping the study (see 6.6.3). If the proportion of patients dead and/or requiring post-enrolment invasive mechanical ventilation after inclusion reaches 35% of above of the patients included in the study (analyzed by incremental groups of 50 patients), the enrolments will be temporarily discontinued and DSMC will be asked to review the data and decide on an early permanent discontinuation of the study (see 6.6.3).

References:

- study ACTT-1 (Beigel JH. NEJM 2020; 383:1813-26)

- study GS-US-5773 (Goldman JD. NEJM 2020; 383:1827-37)

- study CORIMUNO-TOCI (Hermine O. JAMA Intern Med 2020; Oct 20 (online ahead of print)

The number of patients requiring mechanical ventilation after inclusion will be tracked over time.

The thresholds requiring DSMC intervention or premature discontinuation of the study have been defined in incremental groups of 50 patients as follows:

| Number of patients included | Number of patients requiring invasive mechanical ventilation after inclusion | |
| --- | --- | --- |
|  | For DSMC intervention | For temporary discontinuation of the study |
| Up to 50 | 10 | 18 |
| 51 to 100 | 20 | 35 |
| 101 to 150 | 30 | 53 |
| 151 to 200 | 40 | 70 |
| 201 to 250 | 50 | 88 |
| 251 to 300 | 60 | 105 |
| 301 to 352 | 70 | 124 |
| 353 to 398 | 80 | 140 |

These criteria applied to this day may need to be modified according to the evolution of the epidemic.

A definitive or temporary discontinuation of all or part of the study may be decided by ANSM, the Ethics Committee, the Sponsor after Data and Safety Monitoring Committee (DSMC) opinion*.*

In any case:

- A written confirmation of this early discontinuation of the study shall be sent to the coordinating investigator of the study (specifying the reasons for the early discontinuation),

- All the patients included in the study shall be informed and should attend their early withdrawal visit.

## Patient medical care at the end of the study

After the end of the study, each patient will receive the usual medical follow‐up.

# Data Management AND STATISTICS

## Data entry and data collection

### Data entry, processing and circulation

An electronic data capture system, eCRF, will be used for data collection in this study.

Each person (investigator, CRT, Project Manager, CRA) has a personal user account in connection with their role (profile) that has been assigned to them by the trial sponsor. The creation and administration of computer accounts are managed by the Data Manager in charge of the test.

Each user must change their initial password on first login, using an encrypted password of at least 8 alphanumeric characters, valid for 30 days. The system will revert to standby after 15 minutes of inactivity on the application, and users will be blocked after three unsuccessful login attempts. Each person will be trained for the use of the eCRF

Data collection will be done directly by the investigator or CRA in charge of the study, using an eCRF accessible since the web site <https://nantes-lrsy.hugo-online.fr/EnnovClinical> and developed by the Promotion Department of the University Hospital of Nantes with Ennov Clinical software.

The eCRF is designed to record the data required by the protocol and collected by the investigator. The investigator must ensure that data are recorded and any corrections in the eCRF are made as stated in the study protocol and in accordance with the instructions. The investigator must ensure that the recorded data is correct, complete, and that reporting takes place according to the timelines that have been predefined and agreed. The investigator signs the completed eCRF.

### Patient identification

The principal investigator and all co-investigators undertake to keep the identities of the persons who participate in the study confidential by assigning them a code.

This code will be used for all the eCRF and all the attached documents (reports of imaging exams, biology, etc.). It will be the only information which will make it possible to make the connection with the patient retrospectively.

The coding rule is the following: 1st letter of first name + first letter of surname and Inclusion number.

### Encoding data

The sponsor is required to encode patient data on all documents that may be in his/her possession (reports from biological analysis, etc.) that are attached to the CRF. All the documents attached to the SAE report will remain encoded.

### Encoding data

Medical coding:

The data manager is responsible for data coding including:

Medical history and AEs using the latest version of the Medical Dictionary for Regulatory Activities, MedDRA.

Before database lock, all coded terms must be validated by a person involved in the medical review.

Clinical data collection will be based on a clinical database and creating input templates similar to the CRF in compliance with protocol and applicable regulations.

The structure of the database and data entry screens will be approved by the sponsor of the study.

When the database has been declared to be complete and accurate, it will be locked and made available for data analysis.

## Statistics

### Description of planned statistical methods, including planned intermediate analysis schedule

Phase 2a:

The safety population included all subjects randomized into the study who received at least one dose of study drug. The intent-to-treat (exposed, ITT[E]) Population was defined as all subjects who met study criteria and were randomized into the study with documented evidence of having received at least one dose of randomized treatment and at least one post-baseline XAV-19 IgG SARS-CoV2 titers measurement. The Per-Protocol Population was defined as all subjects included in the ITT(E) Population excluding those who had at least one major protocol deviation.

The PK Concentration Population included all subjects who received XAV-19 at least once during the study. The PK Summary Population included subjects with an evaluable pharmacokinetic profile of XAV-19 on Day 8. Subjects were excluded if they missed the second dose of Day 5, only if second administration was planned according to protocol. The PK/PD Summary Population included subjects who met the criteria for both PP Population and PK Summary Population

The primary endpoint will be evaluated for ITTE population by a Kruskal-Wallis test between all placebo patients and all treated patients.

The pharmacokinetic analysis will be evaluated by the study of the accumulation, time-invariance, achievement of steady state, and dose-proportionality by ANOVA models with terms for subjects as a random effect and day as fixed effect.

Groups of treatment will be described according to variables in of the secondary endpoints. Categorical variables will be summarized by percent and continuous variable by means and standard error, and medians and interquartile range.

Phase 2b:

This is a double-blind, placebo controlled randomized trial with a two-sided type I error rate of 0.05.

Categorical variables will be summarized by percent and continuous variable by means and standard error, and medians and interquartile range.

Efficacy analyses

Summaries and analysis of efficacy data will be presented for the intent-to-treat (ITT) population and a Treated population (See §7.2.6).

Primary endpoint:

Proportion of patients who die, develop respiratory failure (requiring non-invasive ventilation, high-flow oxygen devices or invasive mechanical ventilation) between baseline and Day 15 will be compared using a logistic mixed model to take into account stratification factors (duration of symptoms prior to enrolment and sites).

Secondary hypotheses have been ordered according to relative importance.

The key secondary outcome, which is the proportion of patients who die, develop respiratory failure (requiring non-invasive ventilation, high-flow oxygen devices or invasive mechanical ventilation) between baseline and Day 8 and Day29 will be analysed as the primary endpoint.

Others secondary endpoint :

Quantitative endpoint will be compared using linear mixed model and qualitative data with logistic mixed model to take into account stratification factors.

Time data’s will be analyzed using survival model (Kaplan-Meier, Cox model). Fine & Gray method will be used to take into account competing risk.

Safety Analyses

The safety population is defined as all subjects randomized into the study but excluded who are ineligible at baseline or who have not had any dose of treatment.

Safety endpoints include death through Day 29 and through Day 60, SAEs and Grade 3 and 4 AEs. These events will be analyzed univariately. Time-to-event methods will be used for death. Each AE will be counted once for a given subject and graded by severity and relationship to COVID-19 or study intervention. AEs will be coded using the current version of the Medical Dictionary for Regulatory Activities (MedDRA). AEs will be presented by system organ class, duration (in days), start- and stop-date. Adverse events leading to premature discontinuation from the study intervention and serious treatment-emergent AEs will be presented either in a table or a listing.

More details will be described in the Statistical Analysis Plan.

### Statistical justification of the number of inclusions

Phase 2 a:

As it is a Phase 2 a, the number of patients was determined according to relevant publications of phase 2a. It was decided to enrol two cohorts of 8 patients (6 treated patients and 2 matching placebo in each cohort).

Phase 2 b:

Assuming a true treatment diminution in clinical failure rate of 10% for XAV-19 added to SoC therapy compared to placebo added to SoC therapy where a rate of 20% is expected, a sample size of 398 randomized participants provides at least 80% power that the primary analysis will be statistically significant at the two-sided 5% significance level.

The 20 patients of the open-label ancillary terminal study (150 mg dose of XAV-19) will not be included in the primary analysis. Their pharmacokinetic characteristics and variability will be compared to the parameters of randomized phase 2b patients taking part in the ancillary pharmacokinetics ancillary study and immunomonitoring ancillary study.

### Expected level of statistical significance

The degree of significance is set at 5%.

### Consideration method for missing, unused or invalid data

Phase 2a:

No imputation will be realized in this study.

Patient who did not receive both doses (first cohort phase 2a and first 2 patients of the second cohort of phase 2a) or one dose (6 last patients of the second cohort of phase 2a and phase 2b), according to protocol or followed up until D8 will be replaced.

Phase 2b:

No randomized subject replacement is planned.

We do not expect missing data for the primary outcome.

All attempts will be made to collect all data per protocol. Any data point that appears to be erroneous or inexplicable based on clinical judgment will be investigated as a possible outlier. If data points are identified as outliers, sensitivity analyses may be performed to examine the impact of including or excluding the outliers. Any substantive differences in these analyses will be reported.

For the primary outcome, if patient is not followed up until D15 then, data will be imputed by a multiple imputation procedure and a sensitivity analysis will be performed on the imputation method,

For the analyses of the secondary outcomes that involve clinical score (i.e. the key secondary outcome and time to improvement), if a subject is discharged from the hospital without a previously or concurrently reported clinical score of 2 or 1, then their clinical score at the time of discharge will be imputed as 2, which is the highest value for a non hospitalized subject.

For the analyses of the secondary outcomes, the following imputation rules will be used for subjects who are lost to follow-up, terminate early from the study, or do not have further outcome data available after discharge for any reason:

• Days of Non-invasive ventilation/high-flow oxygen:

If the subject’s clinical status scale is 5 at the last observed assessment, then the subject will be considered to be on non-invasive ventilation/high-flow oxygen. The endpoint will be total days when assessments are available plus all imputed days following the last observed assessment.

If the subject is not on non-invasive ventilation/high-flow oxygen at the last observed assessment, then the subject will be considered to not be on non- invasive ventilation/high-flow oxygen for the remainder of follow-up. Thus, no additional imputed days will be added to the number of days recorded on available assessments.

• Days of intubation or mechanical ventilation:

If the subject’s clinical status scale is 6 at the last observed assessment, then the subject will be considered to be on non-invasive ventilation/high-flow oxygen. The endpoint will be total days when assessments are available plus all imputed days following the last observed assessment.

If the subject is not on non-invasive ventilation/high-flow oxygen at the last observed assessment, then the subject will be considered to not be on non- invasive ventilation/high-flow oxygen for the remainder of follow-up. Thus, no additional imputed days will be added to the number of days recorded on available assessments.

• Days of ventilation/ECMO:

If the subject’s clinical status scale is 7 at the last observed assessment, then the subject will be considered to be on ventilation/ECMO through Day 29. The endpoint will be total days when assessments are available plus all imputed days following the last observed assessment.

If the subject is not on ventilation/ECMO at the last observed assessment, then the subject will be considered to not be on ventilation/ECMO through Day 29. Thus, no additional imputed days will be added to the number of days recorded on available assessments.

• Days of Oxygen:

If the subject’s clinical status score is 4, 5, 6, or 7 at the last observed assessment, then the subject will be considered to be on oxygen through Day 29. The endpoint will be total days when assessments are available plus all imputed days following the last observed assessment.

If the subject is not on oxygen at the last observed assessment, then the subject will be considered to not be on oxygen through Day 29. Thus, no additional imputed days will be added to the number of days recorded on available assessments.

• Days of Hospitalization

If the subject is discharged and no further hospitalization data are available, then the subject will be assumed to not have been readmitted. Thus, no additional imputed days will be added to the number of days recorded on available assessments. If a subject dies while hospitalized, the number of days of hospitalization will be imputed as 29 days.

### Management of changes made to the initial analytical strategy

We do not expect modifications of the initial analysis strategy.

### Choice of subjects to be included in analysis

The safety population included all subjects randomized into the study who received at least one dose of study drug.

For phase 2 a:

The intent-to-treat (exposed, ITT[E])Population is defined as all subjects who met study criteria and were randomized into the study with documented evidence of having received at least one dose of randomized treatment and at least one post-baseline XAV-19 IgG SARS-CoV2 titers measurement.

The Per-Protocol Population is defined as all subjects included in the ITT(E) Population excluding those who had at least one major protocol deviation.

For phase 2 b:

The intent-to-treat (ITT) Population is defined as all subjects randomized into the study.

The Full Analysis Set population includes the ITT population but excludes subjects who are ineligible at baseline. Patient with withdrawal of consent will be conserved in the full analysis set

The Per-Protocol Population is defined as all subjects of the full analysis set population which received at least one dose of randomized treatment excluding those who had at least one major protocol deviation.

### Randomisation

Phase 2 a:

- Two patients will be first enrolled (1 placebo, 1 treated with dose 1). Then after 8 days, if no adverse events are observed and after authorisation of the DSMC, 6 other patients will be enrolled (1 placebo, 5 treated with dose 1).
- The committee will then evaluate the safety and tolerability of all the included patients after 8 days post randomisation. If the committee gives its authorization, the second cohort will start.
- Two patients will be first enrolled (1 placebo, 1 treated with dose 2). Then after 8 days, if no adverse events are observed and after authorisation of the DSMC, 6 other patients will be enrolled (1 placebo, 5 treated with dose 2).

Phase 2b:

A randomisation stratified by center and by duration between onset of COVID-19 symptoms and screening (1-6 days, 7-10, 11 to 14) will be settled.

Randomization will be double-blinded. It will be carried out in a 1:1 ratio and will be balanced by blocks.

The randomization will be carried out via EnnovClinical software by connecting to the website: <https://nantes-lrsy.hugo-online.fr/>. The connection will be made using a login, a password and a study number (NTLRXXX), issued by a data manager of the Research Promotion Department of the Nantes University Hospital.

The following information must be filled in:

- First initial of the name

- First initial of first name

- Year of birth

- Compliance with inclusion and non-inclusion criteria (yes/no)

- Informed consent (yes/no).

The inclusion number and arm of randomization will be assigned automatically at randomization. An e-mail confirmation will be sent to the randomizer and to all concerned.

The randomization lists will be drawn up by a statistician from the Research Promotion Department of the Nantes University Hospital. An explanatory guide to randomisation will be available online under EnnovClinical.

# Pharmacovigilance and adverse event management

## Definitions

| Vigilance | Science and activities relating to the detection, assessment, understanding and prevention of adverse effects or any other medicine-related problem. |
| --- | --- |
| Adverse events (AE) | Any untoward medical occurrence in a person participating in a research on human being whether or not considered related to the product or the research. |
| Adverse Event Intensity (EvI) | Rated according to the Division of AIDS (DAIDS) Table for Grading the Severity of Adult and Pediatric Adverse Events (version 2.1 dated July 2017). Any event not rated in the selected classification should be rated as follows:  *Grade 1: Mild symptoms causing no or minimal interference with usual social & functional activities with intervention not indicated*  *Grade 2: Moderate symptoms causing greater than minimal interference with usual social & functional activities with intervention indicated*  *Grade 3: Severe symptoms causing inability to perform usual social & functional activities with intervention or hospitalization indicated*  *Grade 4: Potentially life-threatening symptoms causing inability to perform basic self-care functions with intervention indicated to prevent permanent impairment, persistent disability.*  *In addition, all deaths related to an AE are to be classified as grade 5.* |
| Adverse reactions (AR) | All untoward medical occurrences in a person participating in a research on human being, when this response is related to the research or the investigational medicinal product (IMP). |
| Adverse reaction of an experimental medicinal product – Adverse Drug Reaction (ADR) | All untoward and unintended responses to an investigational medicinal product related to any dose administered. |
| Serious adverse events (SAE) | Any untoward medical occurrence or effect that :  - results in death,  - is life-threatening,  - results in persistent or significant disability or incapacity,  - requires hospitalisation or prolongation of existing hospitalisation,  - is a congenital anomaly or birth defect.  - is medically significant (the list of medically significant events/reactions is defined by the EMA) |
| Unexpected adverse reactions | An adverse reaction, the nature or severity of which is not consistent with the applicable product information. |
| Suspected Unexpected Serious Adverse Reactions (SUSAR) | An untoward and unintended response to an investigational medicinal product, which is not listed is the applicable product information, and meets one of the serious criteria. |
| New safety information | Any new data which could :   - Induce new evaluation of benefit/ risk ratio of the study or of the product object of the study, - modify product utilization, the conduct of the study or documents related to the study - Suspend or terminate the protocol under research or similar researches.   For trials on first administration or non-health product with person without any affection: all adverse effect. |
| Abuse | This corresponds to the persistent or sporadic, intentional excessive use of a medicinal product, which is accompanied by harmful physical or psychological effects. |
| Overdose | This refers to the administration of a quantity of a medicinal product given per administration or cumulatively, which is above the maximum recommended dose according to the authorized product information. Clinical judgement should always be applied.  (Real overdose: due to a brut excessive amount / relative overdose: due to patient predisposal factors as renal insufficiency, hypo-albuminuria…) |
| Misuse or use outside marketing authorisation | This refers to situations where the medicinal product is intentionally and inappropriately used not in accordance with the authorised product information. |
| Quality defect | Non conformity to the specifications described in the marketing authorisation file / CE marking / technical documentation or deviation against good manufacturing practices / good distribution/storage/labelling practices. |
| Medication Error | Medication errors are unintended mistakes (proved or potential) during the care process, in the circuit (from manufacturing to administration) implying a product that can lead to a risk or an adverse event for the patient.  The risk of error or potential error concerns situations where the error did not happen, was intercepted but could have happen. |

## Safety evaluation parameters

### Specific safety-related evaluation criteria

All patients will be closely monitored during both study phases for overall biological and clinical safety data (eg. infections or malignancies) and any drugs toxicity.

According to regulation, each AE/AR reported by the patient or identified by the investigator must be collected and reported to sponsor, as soon as he is aware, if it meets to seriousness criteria from inclusion of the subject, to the end of the participation.

As a precaution, patients enrolled in this trial must be observed during infusion of study drug and for at least 2 hours post infusion. Patients will be assessed for signs or symptoms of any infusion reactions (e.g., hypotension, hypoxia, tachycardia, fever, nausea, fatigue, headache, myalgia, and malaise).

The safety assessment in this study will be based on physical examination, vital signs, AEs, clinical laboratory tests and other biological tests and ECG, as described in the sections 5.2 (Procedure Details).

New symptoms suspected to be related to COVID-19/SARS-CoV-2 infection that start after the initiation of study treatment should be reported as AEs.

Worsening of symptoms related to COVID-19/SARS-CoV-2 infection should be closely medically analyzed, and should be reported as AE if, the symptoms are clinically significant and/or lead to treatment modification :

- For instance, in case of increase of oxygen requirement, investigators, taking into account the full clinical situation, will judge whether this increase in O2 treatment is related to a clinically significant complication or worsening that shall then be considered as either an AE or notified as a SAE

- In case of transfer to ICU, investigators will consider the reason responsible for this patient's management (worsening of COVID-19/SARS-CoV-2 infection, concomitant disease, or only local logistical management) to decide if AE or SAE must be considered. Clinically significant symptoms without acute severity criteria (e.g., transfer to the intensive care unit for close monitoring, including optiflow or NIV requirement) should be considered AE.

New symptoms leading to a risk of short-term mortality due to their severity should be reported as SAE, e.g. mechanical ventilation or high-flow oxygen therapy for patients in whom mechanical ventilation is not considered for ethical reasons.

### Methods and schedule envisaged to measure, compile and analyse safety evaluation parameters

Any AR/AE whether expected or unexpected, serious or not, must be real-time collected in the study eCRF.

Safety assessment will be followed at each visit.

## List of expected ARs

In this protocol, the expected Adverse Event and Reactions are associated with drug under study, it placebo, and disease under study.

**Expected ARs for IMP,** infusion-related reactions may occur with antibody-related products and has been reported with other agents.

Adverse reaction as complete or incomplete flu-like symptoms, characterized by fever, headache, chills, myalgia or fatigue, hypersensitivity signs, can be observed.

According to IB, no serious adverse reaction have been observed with glyco-humanized swine IgG infusions, all serious reaction that will be observed will be considered as SUSARs.

**Expected ARs for Placebo,** only local AEs with pain, erythema, irritation are expected for placebo (NaCl solution); the amount of NaCl does not suggest systemic hydro electrolytic or blood pressure adverse effects, nor infection.

**Concerning the disease**, the most frequent expected serious adverse events are as follows:

- events related to COVID-19 infection: progression (or worsening) or complications of pneumonia, occurrence of acute respiratory distress syndrome (ARDS) that may require mechanical ventilation and/ or ECMO.

- other complications such as septic shock, cardiac arrest, myocardial infarction, respiratory failure, stroke, cardiogenic shock, ARDS, multisystemic failure syndrome (MDS), ... that may be life-threatening or fatal

## Adverse event management

### AR/AE collection

Any AR or AE, whether expected or unexpected, serious or not, must be real-time collected in the study eCRF.

### Management of possible adverse reaction to infusion of XAV-19

If during close monitoring following treatment administration, unintended side effects occur, the patient will be immediately clinically reevaluated to detect any worsening of his/her condition. Because immunoallergic reactions to antibodies administration can present either typically or atypically, and can share some features of the COVID-19 cytokine storm, investigators will have to be very cautious in analysing events occurring following the first infusion, in order to decide whether the second infusion can be administered (for patients in phase 2a). In any situation where there is the possiblity that events following XAV-19 are related to product infusion, early withdrawal from the study will be decided only in phase 2a (see 6.7.).

All possible adverse reactions to infusion of XAV-19 will be managed as indicated by their nature and severity. In case of any severe advent, or COVID-19 worsening, requiring intensive care measures, participant will be immediately transferred to the intensive care unit department.

### SAR/SAE reporting

All SARs/SAEs whether expected or unexpected, must be:

- entered in real-time in the eCRF

- reported immediately (from the day the of the investigator becoming aware of the event) to the sponsor directly through the eCRF

The information mentioned on this form and on joined documents must be complete, accurate, clear (no abbreviation…) and closely coded.

Pregnancy, overdose, misuse, medication errors or potential medication errors, quality defects should be notified by the investigator to the sponsor even if there is no adverse reaction associated.

### Reporting period

All SAR must be reported to the sponsor if it happens for a research participant:

- Since the consent signature date,
- During all the participant follow up period scheduled by the study (28 days)
- After the end of the patient follow-up and without any time limit if the investigator becomes aware of a SAR possibly linked to the experimental treatment.

### Data and Safety Monitoring Committee (DSMC)

The role of the DSMC is to review the progress of the trial and the accumulating data to detect evidence of early safety issues for the enrolled subjects. The DSMC will give recommendation regarding modification to the ongoing conduct of the trial (see 1.2.4) and give approval for continuing the study to the next step within each group of phase 2a and between the phase 2a and 2b.

The competent members in the field of clinical trials (disease and methodology) are not involved in the study. They are appointed for the period of the study and undertake to participate and to respect the data confidentiality.

The annual safety report is sent to the Data and Safety Monitoring Committee. The committee may be requested for a review by the person in charge of safety pharmacology if a SUSAR or a SAE presents a particular analytical problem or if a doubt in respect benefit/risk arises during the study.

The list of members of the Data and Safety Monitoring Committee is attached in **Appendix 3.**

## Follow-up procedure and period for subjects following the onset of adverse events

### Procedure to follow for the patient concerned by the SAE

All events must be followed up until recovery, consolidation or death (event closed).

If a pregnancy occurs during the study, this should be followed up at least until birth or even until the child reaches adulthood.

Delayed adverse reactions (malformation, secondary cancer, etc.) must be reported to the sponsor (if known to the investigator) even after the end of the study.

# Administrative and regulatory aspects

## Source data and document access rights

Each patient's medical data shall only be provided to the sponsor or any person duly authorised by the sponsor, and, where applicable, to authorised health authorities, in confidential conditions.

The sponsor and the supervisory authorities may request direct access to medical records for the purposes of verification of the procedures and/or data in respect of the clinical trial, within the limits authorised by the legislation and regulations.

The data compiled during the trial may be processed electronically in compliance with CNIL requirements.

## Trial monitoring

Monitoring shall be carried out by the Research Division Promotion Department. A Clinical Research Associate (CRA) shall visit each site (investigator and dispensary) regularly to conduct quality control on the data reported in the case report forms.

The protocol has been classified according to the estimated level of risk for the patient taking part in the study. It shall be monitored as follows:

Risk D: very high foreseeable risk

The on-site monitoring visits shall be organised after making arrangements with the investigator. The CRAs should be able to consult on each site:

- the enrolled patients' data compilation records,

- the patients' medical and nursing files,

- the investigator file.

- the treatment storage and dispensation place

## Scientific Committee

### Composition

The list of members of the Scientific Committee is attached in **Appendix 4.**

### Frequency of meetings

The first meeting of the Scientific Committee should be organized prior to the start of the research, and if possible before initiating regulatory procedures to validate all scientific, ethical and logistical aspects of the trial.

For phase 2a, meetings will occur after each DSMC review of data.

For phase 2b, meetings are scheduled twice a year until the end of the research according to the specificities and the progress of the project. Extraordinary meetings may be decided by the chairperson of the Scientific Committee upon request from the Sponsor, or from one or several members of the Scientific Committee. The request motivated in writing shall be diffused for information to all members of the Scientific Committee.

### Role

The Scientific Committee's missions are:

- approval of DSMC composition,

- to ask for information regarding the progress of the research project, any potential issue and available results,

- to ensure compliance with ethics requirements,

- to perform the scientific follow-up of the research: maintain the relevance of the research objectives and the permanent validity of the methods implemented to meet them,

- to make all important decisions at the demand of the Coordinating Investigator or the DSMC regarding the good conduct of the research in compliance with the protocol, any procedure specific to the research and Good Clinical Practices,

- to decide on all relevant modification of the protocol required to achieve the research project (including recruitment facilitating measures, protocol amendments before regulatory submissions, addition or closure of participating sites),

- to provide information to all investigators and other participants in the research,

- to ensure that rules related to data and biological samples access are followed,

- to ensure that rules related to the communication and publication of research results are fulfilled.

Meeting minutes are drafted following each meeting by the Project Leader, together with the president of the Scientific Committee (at least an exhaustive list of the issues discussed and the decisions made as well as the list of the points raised). The minutes are submitted for review and modifications to the members of the Scientific Committee present at the meeting. The minutes are then validated by the president of the Scientific Committee. It is then distributed to the Scientific Committee members and to the persons invited to the meeting, as well as to the Head of the Nantes Hospital clinical research department. The minutes are definitely adopted at the beginning of the following meeting of the Scientific Committee.

## Inspection / Audit

Within the scope of this study, an inspection or audit may be conducted. The sponsor and/or participating centres should be able to provide inspectors or auditors with access to the data.

## Ethical considerations

### Written informed consent

The investigator agrees to provide the subject with clear and precise information about the protocol and request from him/her a written and signed consent form (information form and consent form appended). The investigator shall give the subject a copy of the information form and consent form. The subject can only be enrolled in the study after reading the information form and signing and dating the consent form, after taking time to reflect on the matter.

The investigator shall also sign and date the consent form. Both documents should be issued at least in duplicate hard copy format so that the patient and the investigator can each keep a copy. The investigator's original shall be placed in the investigator file. If the consent form is signed in duplicate, the investigator keeps the original and gives the copy to the subject.

### Ethical Review Board

The sponsor undertakes to submit the draft study to the Ethical Review Board (ERB) for prior approval.

## Registration with the competent authorities

This protocol shall be the subject of an ANSM authorisation application prior to its start. At the end of the phase 2a, inclusions will be stopped and a substantial modification will be submitted to the ANSM to obtain authorization to continue to the phase 2b.

## Amendments to the protocol

Requests for substantial modifications should be addressed by the sponsor for approval or notification to ANSM and/or the Ethical Review Board concerned in compliance with the law and its implementing decrees.

The amended protocol should be a dated updated version.

The patient information and consent forms should be amended if required.

## Study funding and insurance

The sponsor shall fund the study and take out an insurance policy covering the financial consequences of its civil liability in compliance with the regulations.

## Publication rules

This clinical study is carried out within the framework of the PSPC POLYCOR project, which is the subject of a consortium agreement negotiated and signed by all project partners.

The information and data collected during the conduct of this clinical study are considered confidential and will be used by the Sponsor and its partners in connection with the development of the study drug. All the results of this study including data reports, discoveries and inventions resulting from the study, are the property of the Sponsor CHU de Nantes.

The Sponsor is the holder of all exclusive exploitation rights relating to the project, and in particular the right to publish, subject to the rights of third parties and in particular the stipulations of the POLYCOR consortium agreement.

The results of this study may be published or presented at scientific meetings. The CHU de Nantes, as Sponsor, has the right to make the first publication on the results of the clinical study. Publications and communications should mention the contribution made by each partner to the implementation of the Project. If this is envisaged, the Coordinating Investigator, partners and coauthors agree to submit all manuscripts or abstracts to Sponsor prior to scientific meeting or journal submission allowing for reasonable time to review, consistent with Sponsor policy. This allows the Sponsor to protect proprietary information and to provide medical/scientific review.

For intellectual property protection purposes, Xenothera can also request the coauthors to delay publication or presentation of results for a period of eighteen (18) months from the date of application.

In accordance with consistent editorial practice, Sponsor supports the publication of primary study results in their entirety prior to any secondary analyses.

A copy of the publication shall be delivered to Nantes University Hospital, the study sponsor, which shall necessarily be cited.

The sponsor will enter the study results in the European Union database as soon as the main publication from the research is released, in order to preserve intellectual property.

## Outcome of biological samples

At the end of the study, residual biological samples resulting from sampling (blood sample and respiratory from nasopharyngeal swab collection) for all patients from Phase 2a and for all patients from Phase 2b will be kept. In the latter case, the subject's written consent will be collected at screening and the samples stored will be integrated into the biocollection “POLYCOR” located in the Centre de Ressources Biologiques (CRB) of the Nantes University Hospital, under the responsibility of Dr Gaborit.  This biocollection will be attached to the "Immunologie" research program declared on 05/09/2011 under the n° DC-2011-1399 and in the following amending declarations (DC-2012-1555; DC-2013-1832; DC2014-2206 and DC-2017-2987 currently pending) at the Ministry of Research and having obtained a favorable decision from the CPP Ouest IV on 07/04/2015.

## Source data archiving

The investigator should archive all study data for at least 15 years after the end of the study.

At the end of the study, the investigator shall also receive a copy of the data for each patient in the investigator's centre sent by the sponsor.

#

**STATISTICAL ANALYSIS PLAN**

| ***Trial acronym*** | *POLYCOR* |
| --- | --- |
| ***Trial registration number*** | **NCT04453384** |
| ***Study title*** | ***A randomized, double-blind, placebo-controlled phase 2 (2a and 2b) study to evaluate the safety and efficacy of XAV-19 in patients with COVID-19 induced moderate pneumonia*** |
| ***Protocol version*** | *Version 7.0* |

Prepared by:

*Aurélie LE THUAUT, Marie-Anne VIBET*

Plateforme de méthodologie et Biostatistique

DRI, CHU de Nantes

5 allée de l’Ile Gloriette 44093 NANTES cedex1

Email : marieanne.vibet@chu-nantes.fr et aurelie.lethuaut@chu-nantes.fr

Signature page for SAP

Protocole title : A randomized, double-blind, placebo-controlled phase 2 (2a and 2b) study to evaluate the safety and efficacy of XAV-19 in patients with COVID-19 induced moderate pneumonia

| **Version** | **Date** | **Author** | **Detail** |
| --- | --- | --- | --- |
| *1.0* | *29/04/2021* | *Marie-Anne Vibet*  *Aurélie Le Thuaut* |  |
| *2.0* | *18/06/2021* | *Marie-Anne Vibet*  *Aurélie Le Thuaut* | *Modifications done on the primary endpoint to take into accound LAT patients and after discussion with Xénothera* |
|  |  |  |  |
|  |  |  |  |
|  |  |  |  |
|  |  |  |  |

| **NAME** | **FUNCTION** | **DATE** | **SIGNATURE** |
| --- | --- | --- | --- |
| Marie-Anne Vibet | *Statistician* | 29/04/2021 | 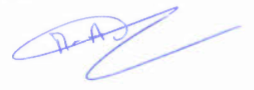 |
| Aurélie Le Thuaut | *Statistician* | 29/04/2021 | 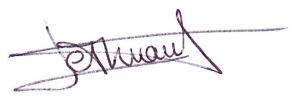 |
| Benjamin Gaborit | *Coordinating investigator* | 07/05/2021 | 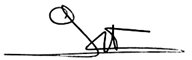 |
| Aurélie Le Thuaut | *Statistician* | 18/06/2021 | 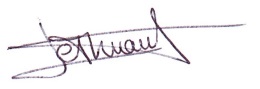 |
| Marie-Anne Vibet | *Statistician* | 18/06/2021 | 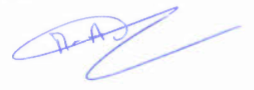 |
| Benjamin Gaborit | *Coordinating investigator* | 19/06/2021 | 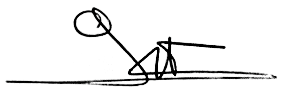 |

***Table of contents***

[List of abbreviations 4](#_Toc70589469)

[1. Introduction 5](#_Toc70589470)

[1.1 Background and rationale 5](#_Toc70589471)

[1.2 Study objectives 6](#_Toc70589472)

[1.3 Endpoints 8](#_Toc70589473)

[2. Study Methods 10](#_Toc70589474)

[2.1 Trial design 10](#_Toc70589475)

[2.2 Study Population 10](#_Toc70589476)

[2.3 Intervention 11](#_Toc70589477)

[2.4 Randomization 11](#_Toc70589478)

[2.5 Critical COVID and death follow-up 11](#_Toc70589479)

[2.6 Timing of final analysis 12](#_Toc70589480)

[2.7 Sample size calculation 13](#_Toc70589481)

[3. General statistical considerations 14](#_Toc70589482)

[3.1 General Principes 14](#_Toc70589483)

[3.2 Protocol deviations 14](#_Toc70589484)

[3.3 Population definition 14](#_Toc70589485)

[4. Trial population 15](#_Toc70589486)

[4.1 Screening data 15](#_Toc70589487)

[4.2 Withdrawal/Follow-up-level of withdrawal 15](#_Toc70589488)

[5. Statistical methods 18](#_Toc70589489)

[5.1 Outcome definitions 18](#_Toc70589490)

[5.2 Analysis methods 21](#_Toc70589491)

[5.3 Covariates and Subgroups 23](#_Toc70589492)

[5.4 Missing Data 24](#_Toc70589493)

[6. References 25](#_Toc70589494)

**List of abbreviations**

| **Abbreviation or Specialist Term** | **Explanation** |
| --- | --- |
| *AE* | *Adverse event* |
| *ALT* | *Alanine aminotransferase* |
| *AST* | *Aspartate aminotransferase* |
| *CI* | *Confidence interval* |
| *CRF* | *Case Report Form* |
| *DSMB* | *Data and Safety Monitoring* |
| *ECMO* | *Extra Corporeal Membrane Oxygenation* |
| *FAS* | *Full Analysis Set* |
| *ICU* | *Intensive care unit* |
| *ITT* | *Intent-to-treat* |
| *mITT* | *Modified Intent-To-Treat* |
| *N* | *Number of subjects* |
| *NEWS* | *National Early Warning Score* |
| *PP* | *Per protocol* |
| *RT-PCR* | *Reverse Transcriptase Polymerase Chain Reaction* |
| *SAE* | *Serious adverse event* |
| *SAP* | *Statistical analysis plan* |
| *SARS-Cov-2* | *Severe acute respiratory syndrome coronavirus 2* |
| *SAS* | *Statistical Analysis System (by SAS Institute)* |
| *SD* | *Standard deviation* |
| *SOC* | *Standard-of-care* |
| *WHO* | *World Health Organization* |

# Introduction

## Background and rationale

Early inhibition of entry and replication of the severe acute respiratory syndrome coronavirus 2 (SARS-CoV-2) is a very promising therapeutic approach. Polyclonal neutralizing antibodies offers many advantages such as providing immediate immunity, consequently blunt an early pro-inflammatory pathogenic endogenous antibody response and lack of drug-drug interactions1–3.

Because a suboptimal endogenous early antibody response with regard to SARS-CoV-2 replication in severe cases is observed, neutralising antibody treatment can be very interesting for patient with COVID-19 induced moderate pneumonia4,5.

A new polyclonal humanized anti-SARS-CoV2 antibodies (XAV-19) is being developed by Xenothera, which can be administered as intravenous treatment. XAV-19 is a heterologous swine glyco-humanized polyclonal antibody (GH-pAb) raised against the spike protein of SARS-CoV-2, inhibiting infection of ACE-2 positive human cells with SARS-CoV-2. Pharmacokinetic and pharmacodynamic studies have been performed in preclinical models including primates and a First In Human study with another fully representative GH-pAb from Xenothera is ongoing in volunteer patients recipients of a kidney graft. These studies indicated that 5 consecutive administrations of GH-pAbs can be safely performed in humans.

The objective of this 2-steps phase 2 randomized double-blind, placebo-controlled study is 1) to define the optimal and safety XAV-19 dose to administrate in patients with COVID-19 induced moderate pneumonia ; 2) to show the clinical benefit of selected dose of XAV-19 when administered to patients with COVID-19 induced moderate pneumonia.

In the first phase (2a) of this study, the terminal half-life (median, range) was estimated at 11.4 (5.5-13.9) days for 2 mg/kg of XAV-19 at day 1. Serum XAV-19 concentrations were above the target concentration of 10 µg/mL (tow fold the in vitro 100% inhibitory concentration [IC100]) from the end of perfusion to more than 8 days for XAV-19 2 mg/kg at day 1. No hypersensitivity or infusion-related reactions were reported during treatment and there was no discontinuation for adverse events and no serious adverse events related to study drug. In this phase 2a study, XAV-19 was well tolerated in patients admitted to hospital for COVID-related moderate pneumonia. The pharmacokinetic results of a single infusion of 2 mg/kg suggest that this dose has the potential to successfully block viral diffusion and supports the selection of this regimen for the phase 2b trial.

The statistical analysis plan described here only tackles the second step of this study.

## Study objectives

### Primary Objective

To evaluate the efficacy of XAV-19 + standard-of-care (Soc) therapy compared with placebo + Soc therapy for treatment of COVID-19 assessed by the proportion of patients who die or develop respiratory failure between baseline and Day 15.

### Secondary Objectives

**The key secondary objectives** are to evaluate the efficacy of XAV-19 + standard-of-care (Soc) therapy compared with placebo + Soc therapy for treatment of COVID-19 between baseline and Day 8, and then between baseline and Day 29.

**The others secondary objectives** are to evaluate clinical efficacy and safety of the investigational therapeutic compared to the control arm, assessed by:

**Clinical severity**:

a) National Early Warning Score (NEWS) over 29 days:

- Change from baseline to Day 3, 5, 8, 15 and 29

- Time to discharge or a NEWS <=2 and maintained for 24 hours

b) 8-point ordinal scale over 29 days

- Time to improvement of one category from baseline

- Time to respiratory failure

- Mean change in ordinal scale from baseline to Day 3, 5, 8, 15 and 29

- Percentage of subjects reporting each severity rating on an 8-point ordinal scale at Day15

c) Improvement of clinical and biological parameters over Day 15

d) Oxygenation

- Duration of oxygen therapy over 29 days

- Time to weaning in supplemental oxygen

- Proportion without O2 requirement at D8, D15 and D29

e) Non-invasive ventilation, high-flow oxygen

- Non-invasive ventilation/high flow oxygen use up to Day 29

- Incidence and duration of non-invasive ventilation or high flow oxygen use during the study

- Time to first day with non-invasive ventilation/high flow oxygen

f) Invasive mechanical ventilation / Extra Corporeal Membrane Oxygenation (ECMO)

- Ventilator / ECMO use up to Day 29

- Incidence and duration of new mechanical ventilation or ECMO use during the study

- Time to first day with Invasive Mechanical Ventilation / extracorporeal membrane oxygenation

g) Transfer to ICU by Day 29

h) Hospitalization

- Hospital length of stay (in days)

i) Mortality

- Mortality rate at day 15 and at Day 29 and at Day 60

j) Thrombotic events

- Cumulative incidence of thrombotic events: venous, pulmonary embolism, arterial thrombotic events.

**Safety of XAV-19**

Evaluate the safety of the intervention through 29 days and 60 days of follow up as compared to the control group as assessed by:

- The cumulative incidence of SAE

- The cumulative incidence of grade 3 or 4 AE

- Proportion of discontinuations or temporary suspensions of infusion (stopping without restarting, temporary suspension)

- Changes in white cell count, hemoglobin, platelets, creatinine, ALT, AST, over time

- The cumulative incidence of major or opportunistic bacterial or fungal infections

- The cumulative incidence of hypersensitivity reactions and infusion reactions

**Exploratory analysis in all patients**

- Change in SARS-CoV-2 status (positive or negative viral load) over time
- Time to RT-PCR virus negativity in nasopharyngeal swab samples over 29 days

## Endpoints

### Primary criterion

Patients who died or develop respiratory failure, as defined by the requirement of noninvasive ventilation, high-flow oxygen devices, invasive mechanical ventilation (corresponding to a score of 5 or more on the WHO 8 point ordinal scale*), or patients with low oxygen delivery device and a flow ≥ 10 L / min with reservoir.

Efficacy will be evaluated between baseline (D1 before infusion) and Day 15.

*Cf §5.1.2

### Secondary criteria

**The key secondary endpoints are the**:

Proportion of patients who die, develop respiratory failure, as defined by the requirement of noninvasive ventilation, high-flow oxygen devices , invasive mechanical ventilation, or patients with low oxygen delivery device and a flow ≥ 10 L / min with reservoir between baseline and Day 8, between baseline and Day 29.

**The other secondary endpoints are:**

1. National Early Warning Score (NEWS) assessed while hospitalized and on Day 15 and Day 29
2. Clinical status using the 8-point ordinal scale assessed daily until Day 29
3. Temperature and blood analysis between baseline and Day 15, and Day 29
4. Days of oxygen therapy over 29 days

PaO2 / FiO2 at baseline, Day 5, Day 8, Day 15, Day 29 if available

1. Days of non-invasive ventilation or high flow oxygen (if applicable) up to Day 29
2. Days of invasive mechanical ventilation/ECMO (if applicable) up to Day 29
3. Transfer in ICU
4. Hospital length of stay (in days)
5. All-cause mortality evaluated between baseline and Day 15 and between baseline and at Day 29 and at Day 60
6. Thrombotic events (peripheral venous, pulmonary, arterial)

**Safety of XAV-19 evaluated as:**

- Occurrence of all suspected XAV-19 related adverse effects or Incidence of serious adverse events

- Study drug discontinuation or temporary suspension of infusion

- Proportion of participants with treatment emergent adverse events leading to study drug discontinuation

- Incidence of major or opportunistic bacterial or fungal infections

- Incidence of hypersensitivity reactions and infusion reactions

- White cell count, hemoglobin, platelets, creatinine, ALT, AST, on D1, D3, D5, D8, D15 and D29

**Exploratory analysis:**

- SARS-CoV-2 status (positive or negative RT-PCR) over time (D1, D8, D15, and D29)

- SARS-CoV-2 status viral load over time (D1, D8, D15, and D29)

# Study Methods

## Trial design

The phase 2b will be a double-blind, placebo-controlled randomized study to assess clinical benefit and safety of the phase 2a selected dose of XAV-19 in 398 hospitalized adults with COVID-19 associated moderate pneumonia.

The study is a multicenter trial that will be conducted in up to approximately 40 sites globally. The study will compare an investigational therapeutic agent to a control arm.

Patients will be randomized to either XAV-19 or placebo in a 1:1 ratio and will receive a single dose based on the analysis of the Phase 2a study. Randomization will be stratified by site and duration since onset of COVID symptoms (1 to 6 days, 7-10 days and 11-14 days).

An independent Data and Safety Monitoring Board (DSMB) will actively monitor interim data to make recommendations about early study closure.

## Study Population

### Inclusion criteria

1. Willing and able to provide written informed consent prior to performing study procedures
2. Male or female ≥ 18 years
3. Hospitalized for COVID-19
4. Documentation of SARS-Cov-2 infection before enrolment, by positive SARS-CoV-2 RT-PCR or antigen in any body specimen (nasopharynx, oropharynx, saliva, sputum, bronchoalveolar lavage …) before enrolment
5. Evidence of pulmonary involvement (on lung examination [rales/crackles] and/or chest-imaging [Chest X-ray or computed tomography])
6. Requiring O2 supplement ≤ 6L/min at screening
7. Requiring O2 supplementation with SpO2 ≥ 92% on O2 therapy at screening (or ≥ 90 % if chronic obstructive pulmonary disease)
8. First onset of COVID-19 symptoms ≤ 14 days, among fever and/or chills, headache, myalgias, cough, shortness of breath, whichever as occurred fist (other symptoms such as asthenia not to be considered in this list)
9. WOCBP must have a negative urinary pregnancy test the day of inclusion
10. All sexually active male subjects must agree to use an adequate method of contraception throughout the study period and for 90 days after the last dose of study drug and agree to no sperm donation until the end of the study, or for 90 days after the last dose of XAV-19, whichever is longer
11. Patients with French social security

### Non-inclusion criteria

1. Evidence of multiorgan failure (severe COVID-19)
2. Mechanically ventilated (including ECMO)
3. Receipt of immunoglobulins or any blood products in the past 30 days
4. Psychiatric or cognitive illness or recreational drug/alcohol use that in the opinion of the investigator, would affect subject safety and/or compliance
5. End-stage renal disease (eGFR < 15 ml/min/1,73 m^2^)
6. Child-Pugh C stage liver cirrhosis
7. Decompensated cardiac insufficiency
8. Known allergy, hypersensitivity, or intolerance to the study drug, or to any of its components
9. Females of childbearing potential without contraceptive method, or with positive pregnancy test, breastfeeding, or planning to become pregnant during the study period
10. Current documented and uncontrolled bacterial infection.
11. Prior severe (grade 3) allergic reactions to plasma transfusion
12. Patient participating in another interventional clinical trial
13. Life expectancy estimated to be less than 6 months
14. Patient under guardianship or trusteeship
15. Patient already included
16. Prior hospitalisation in intensive care unit for the current covid-19 episode

## Intervention

The intervention is an infusion at D1.

Patients will be treated once with XAV-19 (at day 1) and monitored regularly during the study period (see flow chart). Patients will be hospitalized at least from Day 1 to Day 3, if discharged before Day 5 the patient will come back for on-site visits Day 8 and Day 15.

## Randomization

Enrollment and randomization of subjects is done online, using the eCRF, the day of the infusion or the day before.

Eligible subjects will be randomized and assigned in a 1:1 ratio to either Xav19+Soc or placebo+Soc, with stratification by site and by duration since onset of COVID symptoms (1-6 days, 7-10 days, 11-14 days).

## Randomization will be double-blinded and balanced by blocks.Stopping guidance - Critical COVID and death follow-up

During the phase 2b study, the DSMC will regularly meets as defined in the DSMC charter and may decide to stop the study prematurely.

Three meetings will be scheduled after the inclusion of D29 of the 50^th^, 150^th^ and 300^th^ patient. Rates of invasive mechanical ventilation and death will be presented and any other information asked by the DSMC.

If the proportion of patients with need of invasive mechanical ventilation and/or death after inclusion reaches to 20% or above (analyzed by incremental groups of 50 patients), the DSMC will be asked to review the data without stopping the study.

If the proportion of patients dead and/or requiring post-enrolment invasive mechanical ventilation after inclusion reaches 35% of above of the patients included in the study (analyzed by incremental groups of 50 patients), the enrolments will be temporarily discontinued and DSMC will be asked to review the data and decide on an early permanent discontinuation of the study.

References:

- study ACTT-1 (Beigel JH. NEJM 2020; 383:1813-26)

- study GS-US-5773 (Goldman JD. NEJM 2020; 383:1827-37)

- study CORIMUNO-TOCI (Hermine O. JAMA Intern Med 2020; Oct 20 (online ahead of print)

The number of patients requiring mechanical ventilation after inclusion will be tracked over time.

The thresholds requiring DSMC intervention or premature discontinuation of the study have been defined in incremental groups of 50 patients as follows

| Number of patients included | Number of patients requiring invasive mechanical ventilation after inclusion (after randomization) | |
| --- | --- | --- |
|  | For DSMC intervention | For temporary discontinuation of the study |
| Up to 50 | 10 | 18 |
| 51 to 100 | 20 | 35 |
| 101 to 150 | 30 | 53 |
| 151 to 200 | 40 | 70 |
| 201 to 250 | 50 | 88 |
| 251 to 300 | 60 | 105 |
| 301 to 352 | 70 | 124 |
| 353 to 398 | 80 | 140 |

These criteria applied to this day may need to be modified according to the evolution of the epidemic.

A definitive or temporary discontinuation of all or part of the study may be decided by ANSM, the Ethics Committee, the Sponsor after Data and Safety Monitoring Committee (DSMC) opinion.

In any case:

- A written confirmation of this early discontinuation of the study shall be sent to the coordinating investigator of the study (specifying the reasons for the early discontinuation),

- All the patients included in the study shall be informed and should attend their early withdrawal visit.

## Timing of final analysis

Unblinding will be performed when all data of Day 29 for all randomized patients in the main study (patients of the terminal ancillary study will not be considered) will be collected and monitored. However, the treatment allocation will only be communicated to all sites when D60 data for all patients are collected and monitored, and the data base is frozen. The analyses of the primary outcome and a series of key secondary outcomes will be produced after unblinding to allow quick scientific communication.

The final analyses of all outcomes, concerning all patients of the phase 2b study, will be performed on the final full locked database and provided in the final report.

## Sample size calculation

In the phase 2b, about 398 randomized patients, 199 patients in each group, will be included.

Assuming a true treatment difference in clinical failure rate of 10% for XAV-19 added to SoC therapy compared to placebo added to SoC therapy where a rate of 20% is expected, a sample size of 398 participants provides at least 80% power that the primary analysis will be statistically significant at the two-sided 5% significance level.

# General statistical considerations

## General Principes

### Confidence intervals and p-values

All applicable statistical tests will be 2-sided and will be performed using a 5% significance level.

No correction for multiple comparisons will be applied; all secondary objectives will be considered as exploratory and results were reported with only effect size estimates with their confidence intervals (CIs). All CIs presented will be 95%CI and 2-sided.

### Statistical software

Data will be analyzed using the SAS software (Version 9.4. SAS Institute Inc, Cary, NC, USA). Other package such as R software may be used if necessary.

## Protocol deviations

The following protocol deviations are pre-defined as major protocol violations with a direct bearing on primary outcome:

- Patients who did not respect treatment scheduled (i.e. for who treatment was not administrated or not entirely administrated)

- Patients who did not received the allocated treatment (treatment cross-over)

- Patients who did not verify all major inclusion criteria and all major non-inclusion criteria

- Patients lost to follow-up before day 15

- Patients who withdrew their consent before day 15

- Patients who had a respiratory failure before treatment administration

Protocol deviations will be identified and classified as major or minor in blind reviews before the database freezing. The number and % of patients with major and minor protocol deviations will be provided by treatment group, with details of the type of deviation. No formal statistical comparison will be done.

## Population definition

**Intent-to-treat (ITT) Population**: The ITT population will include all randomized patients, regardless of their eligibility and any protocol deviations, according to the treatment group to which they were assigned at randomization.

**Modified Intent-to-treat (mITT) Population**: The mITT population will include the ITT population but will exclude:

1. Patients who did not received treatment for any reason (withdrawn consent or investigator decision due to quick worsening of respiratory status before administration)
2. Patients with legal requirements (i.e. guardianship or trusteeship, < 18 years, no signed consent).

**Per-protocol (PP)**: The PP population will include the mITT population but will exclude:

1. Patients with treatment cross over, defined as receiving the other treatment
2. Patients with withdrawn consent between D1 and D15
3. Patients with major inclusion or non-inclusion criteria violation
4. Patients with major protocol deviations listed in section 3.2

The primary endpoint and the key secondary endpoints will be analysed on the ITT, mITT and the PP population.

The other secondary endpoints will be analysed on the mITT population only.

**Safety population**: The safety population will be included all randomized patients who have received the study treatment or placebo.

# Trial population

## Screening data

The number of screened patients (patients with sign consent), number of randomized patients and the reason for non-randomization will be reported for overall population according to consort flow diagram (figure 1) compliant with the CONSORT 2010 standard.

## Withdrawal/ Lost to Follow-up

The level of withdrawal will be tabulate and classified as:

- Withdraw consent before infusion or during follow-up

- Lost to follow-up at any time during study

- Withdraw due to investigator decisions (reasons should be detailed)

The timing of withdrawal and reasons for withdrawal will be provided by treatment group according to consort flow diagram (figure 1) compliant with the CONSORT 2010 standard.

**Figure 1. Flow of participation in the POLYCOR trial.**

Screened patients (**n=**)

Excluded (**n=**)

Withdraw consent before randomisation (n=) *

Degradation / improvement of clinical status (n=)

Other reasons (n=)*

***Enrollment***

Randomized (**n=**)

***Allocation***

Allocated to XAV-19 + Soc arm (**n=)**

Received allocated intervention (n=)

Did not receive allocated intervention (n=)

Allocated to Placebo + Soc arm (**n=)**

Received allocated intervention (n=)

Did not receive allocated intervention (n=)

Died (n=) or completed follow-up (**n=**)

-lost to follow-up (n=)

-withdrawn consent (n=)

-Other reasons (n=)*

Died (n=) or completed follow-up (**n=**)

-lost to follow-up (n=)

-withdrawn consent (n=)†

-Other reasons (n=)*

***60-day Follow-up***

***Primary Efficacy Analysis***

Analyzed (**n=**)

-excluded (n=)*

Analyzed (**n=**)

-excluded (n=)*

**Table 1. Baseline patient’s characteristics**

| **Characteristics** | **Xav19 + Soc** (N=) | **Placebo + Soc** (N=) |
| --- | --- | --- |
| **Baseline demographics and medical history** |  |  |
| **Age** (in years) |  |  |
| Mean (SD) |  |  |
| Category N (%) |  |  |
| <=50 |  |  |
| > 50-65 |  |  |
| > 65-75  > 75 |  |  |
| **Sex (N (%))** |  |  |
| Women |  |  |
| **Height** mean (SD) |  |  |
| **Weight** mean (SD) |  |  |
| **BMI**  Mean (SD)  Category N (%)  <30  30-34  35-40  >40 |  |  |
| **Medical history** |  |  |
| Hypertension  Other chronic cardiovascular disease |  |  |
| Diabetes |  |  |
| COPD  Other chronic lung disease, including asthma |  |  |
| Chronic kidney disease, including dialysis  Cancer  Solid organ transplant  HIV infection  Other immunosuppressive condition, based on investigator’s assessment |  |  |
|  |  |  |
| **Duration of symptoms before enrollment** |  |  |
| Mean (SD) |  |  |
| ≤ 6 days (%) |  |  |
| 7-10 days |  |  |
| 11-14 days  **Concomitant treatment** |  |  |
|  |  |  |
| **NEWS score** N (%)  2-4 |  |  |
| 5-6 |  |  |
| 7 and more  **SpO2 Mean ( SD)**  **O2 supplement**  < 4L/min  ≥4L/min  Variant’s type  COVID serology if possible |  |  |

# Statistical methods

Data on primary and secondary efficacy/safety outcomes will be performed by academic statisticians.

## Outcome definitions

### Baseline Value

For efficacy assessments, the baseline value will be defined as the value obtained at D1 before infusion.

For safety assessments, the baseline value will be defined as the last value obtained before infusion. This value may have been taken during the screening visit (i.e. up to 2 days before) or D1.

### Clinical status: WHO 8 point ordinal scale

The ordinal scale of the clinical status will be assessed at each study visit.

Each day, the worse score of the day will be recorded.

Reference: <https://www.who.int/blueprint/priority-diseases/key-action/COVID-19_Treatment_Trial_Design_Master_Protocol_synopsis_Final_18022020.pdf>


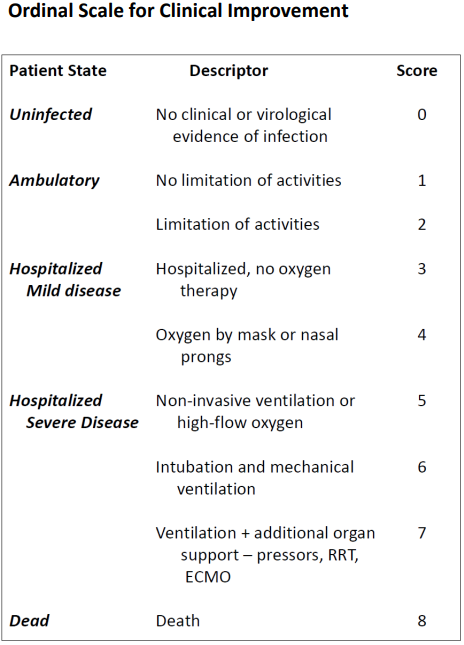


### Respiratory failure or death

Respiratory failure is defined by a requirement of low oxygen delivery device and a flow ≥ 10 L / min with reservoir, or high-flow oxygen devices or noninvasive ventilation, or invasive mechanical ventilation or death after infusion of treatment. This is related to a value of 5, 6, 7, or 8 on the clinical status 8-point ordinal scale.

Respiratory failure will be evaluated between baseline and day 15 (primary objective), between baseline and day 8, and between baseline and day 29 (key secondary objectives).

The primary outcome measures the number of patients who had at least one respiratory failure after baseline (D1 before infusion).

The time to respiratory failure will be defined as the elapsed time (in days) from baseline (on Day 1) to the first day at which a subject reaches any respiratory failure.

### NEWS score (version 2)

NEWS score is an early warning score developed for monitoring hospital in-patients over time using repeated measures.

NEWS has demonstrated an ability to discriminate subjects at risk of poor outcomes.

This score is based on 7 clinical parameters.

Reference: https://www.cebm.net/covid-19/should-we-use-the-news-or-news2-score-when-assessing-patients-with-possible-covid-19-in-primary-care.

The NEWS is based on a simple aggregate scoring system in which a score is allocated to physiological measurements. Six simple physiological parameters form the basis of the scoring system:

- respiration rate
- oxygen saturation
- systolic blood pressure
- pulse rate
- level of consciousness or new confusion
- temperature.

The score is then aggregated and uplifted by 2 points for people requiring supplemental oxygen to maintain their recommended oxygen saturation.


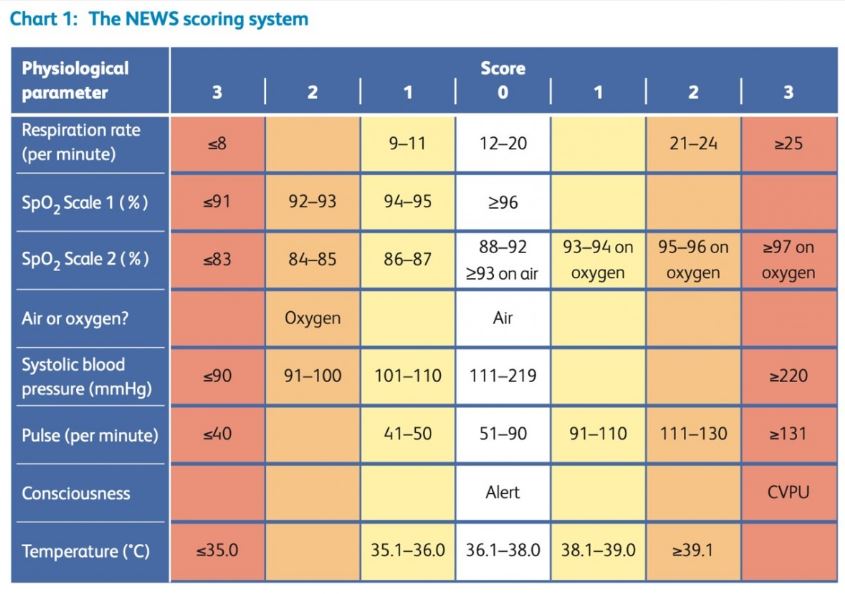


This should be evaluated at the first assessment of a given study day and prior to administration of study product. The 7 parameters can be obtained from the hospital chart using the last measurement prior to the time of assessment and a numeric score given for each parameter (e.g., a RR of 9 is one point, oxygen saturation of 92 is two points).

This score takes into account hypercapnic respiratory failure over time, using scale 2 for Sp02.

### Time to Discharge or NEWS of ≤ 2 over 29 days

The time to discharge or NEWS of ≤ 2 will be defined as the elapsed time (in days) from baseline to the earliest day at which either of the following occurs:

• Discharge from hospital

• Reported NEWS of ≤ 2

### Improvement of clinical and biological parameters

Time to CRP improvement (< 10 mg/l)

Time to lymphopenia improvement (> 1000/mm3)

Change and percentage change in neutrophil-lymphocyte ratio (NLR) at each visit through day 29

### Days of oxygenation

Oxygen days will be defined as the number of days where patients have any oxygen therapy during hospitalization.

The total number of days will be the sum of all reported days, regardless of whether the days occur consecutively or in disjoint intervals.

Oxygen free days will be analyzed over 28 days. 28- Number of days under oxygen. Death will be imputed to 0 day.

### Days of Non-invasive ventilation/high-flow oxygen

Non-invasive ventilation/high flow-oxygen days should be associated with the ordinal scale equal to 5.

The total number of days will be the sum of all reported days, regardless of whether the days occur consecutively or in disjoint intervals.

### Days of Ventilation/ECMO

Ventilator / ECMO days should be associated with the ordinal scale equal to 6 or 7.

The total number of days will be the sum of all reported days, regardless of whether the days occur consecutively or in disjoint intervals.

### Days of Hospitalization

Duration (in days) of the initial hospitalization will be defined as the number of days from D1 to discharge.

### Death

For analysis of death, the time to death will be defined as the elapsed time (in days) from D1 to death.

## Analysis methods

### Baseline characteristics

Detail of baseline characteristics are reported in table 1. Baseline characteristics will be described, in overall and according treatment groups. Quantitative variables will be expressed as mean (standard deviation) or median (interquartile range) for non-Gaussian distribution. Categorical variables will be expressed as frequencies and percentages. The number of missing data will be also reported. No formal statistical comparisons will be done; clinical importance of any imbalance will be noted.

### Primary and key secondary outcome analyses

Proportion of patients who die, develop respiratory failure (requiring noninvasive ventilation, high-flow oxygen devices or invasive mechanical ventilation) between Day 1 and Day 15 (Day 8 and Day 29) will be compared using a logistic mixed model to take into account stratification factors: duration of symptoms prior to screening (fixed effect) and center (random effect).

In cases of convergence failure, sensitivity analysis of the primary outcome will be performed to assess the impact of individual centers on the observed treatment effect. If centers effect is not relevant, final model will not include the center.

### Secondary outcomes analyses

### NEWS score

The median time to discharge or to a NEWS ≤ 2 will be summarized by treatment group.

Time to discharge or to a NEWS ≤ 2 will be compared between groups using Fine & Gray method to take into account competing risk (death).

The mean, standard deviation (SD), median, IQR, minimum, and maximum NEWS at baseline and each study visit will be presented by treatment arm as well as change from baseline.

Change in NEWS score at specific time points will be summarized by proportions (e.g., proportion who have a 1-, 2-, 3-, or 4-point improvement or 1-, 2-, 3-, 4-point worsening).

### 8-point ordinal scale over 29 days

The median time to respiratory failure (value of 5, 6, 7, or 8 on the clinical status 8-point ordinal scale) from baseline will be estimated with Kaplan-Meier method. Frailty model will be used to compare this time by treatment group. If death is a competiting risk (in the case where one death happens before the respiratory failure), a survival analysis with death as competiting risk will be used instead.

The median time to improvement to one category of 8-point ordinal scale from baseline will be summarized by treatment group.

Time to improvement to one category will be compared between groups using Fine & Gray method to take into account competing risk (death).

The proportion of subjects in each severity rating on an 8-point ordinal scale at baseline and at Day 8, Day 15 and Day 29 will be presented by treatment group.

The number and proportion of subjects in each severity rating on an 8-point ordinal scale at Day 15 will be presented. The outcome will be analyzed using an ordinal regression model by treatment group.

### Days of oxygenation

The median time to weaning of oxygenotherapy will be summarized by treatment group.

Time to oxygenation weaning will be compared between groups using Fine & Gray method to take into account competing risk (death).

### Days of Non-invasive ventilation/high-flow oxygen

The median days of non invasive ventilation or high-flow oxygen use during study will be summarized by treatment group. Patients who did not receive non invasive ventilation will have a number of days equal to 0.

Time to non-invasive ventilation/high flow oxygen use will be compared between groups using Fine & Gray method to take into account competing risk (death).

### Days of invasive ventilation /ECMO

The median days of invasive ventilation or ECMO use during study will be summarized by treatment group.

Time to invasive ventilation/ECMO will be compared between groups using Fine & Gray method to take into account competing risk (death).

### Days of hospitalization

The median duration of initial hospitalization will be summarized by treatment group.

Time to discharge will be compared between groups using Fine & Gray method to take into account competing risk (death).

### Transfer in ICU

ICU transfer rate will be summarized by treatment group at day 8, day 15 and day 29.

Time to ICU transfer will be compared between groups using Fine & Gray method to take into account competing risk (death).

### Death

Mortality rate will be summarized by treatment group at day 15, day 29 and day 60.

Time data’s will be analyzed using a stratified log-rank test by treatment groups. Differences will be summarized with Kaplan-Meier curves and 95% confidence bounds.

The median time to event and 95% CI in each treatment group will be summarized and log rank test will be performed. Differences in time-to-event endpoints by treatment arm will be summarized with Kaplan-Meier curves. Number at risk, hazard ratio and log rank p-values will be presented on the figures.

Hazard ratio and 95%CI will be performed using Cox proportional hazards model.

28-day mortality may be summarized according to proportions by category and/or odds ratios with confidence intervals.

### Safety Analyses

Safety endpoints include death through Day 60, SAEs and Grade 3 and 4 AEs.

Each AE will be counted once for a given subject and graded by severity and relationship to COVID-19 or study intervention. AEs will be coded using the current version of the Medical Dictionary for Regulatory Activities (MedDRA). AEs will be presented by system organ class, duration (in days). Adverse events leading to premature discontinuation from the study intervention and serious treatment-emergent AEs will be presented either in a table or a listing.

## Covariates and Subgroups

Subgroup analyses for the main efficacy outcomes will evaluate the treatment effect across the following subgroups:

• Duration of symptoms prior to enrollment

o <= 6 days; 7 to 10 days, 11 to 14 days

• COVID serology at randomization

o Positive

o Negative

• Oxygene Flow at randomization

o < 4l/min

o >=4 l/min

• Immunodepressive status (Solid tumor, leukemia, lymphoma, metastatic solid tumor, HIV)

o Positive

o Negative

• Comorbidities

o Number of conditions : None; One; Two or more

• Clinical status at baseline (NEWS score):

2-4

o 5-6,

o 7 and more

• Concomitant treatment of COVID if possible

• Concomitant treatment of coexisting conditions

• Age

o < 50

o 50-65

o 65-75

o > 75

or by the median (or quartiles) according to the sample size

## Missing Data

All attempts will be made to collect all data per protocol. Any data point that appears to be erroneous or inexplicable based on clinical judgment will be investigated as a possible outlier. If data points are identified as outliers, sensitivity analyses may be performed to examine the impact of including or excluding the outliers. Any substantive differences in these analyses will be reported.

### Primary and key secondary outcome

Subjects who withdrew their consent before Day 15 will be imputed using multiple imputation.

Subjects who are discharged but are subsequently re-admitted prior to Day 15 without a reported 8-point ordinal scale, their ordinal score will be imputed at 7, which is the highest value for a hospitalized subject.

For subjects who are discharged, if no visit is done at day 15 +/ 2 days then the reported ordinal score will be imputed at 2 (highest level for ambulatory patients).

### Clinical severity – 8-point ordinal scale

Subjects who are discharged but are subsequently re-admitted without a reported 8-point ordinal scale, their ordinal score will be imputed at 7, which is the highest value for a hospitalized subject.

For subjects who are discharged, if no visit is done then the reported ordinal score will be imputed at 2 (highest level for ambulatory patients).

For subjects who are discharged from the hospital without a previously or concurrently reported clinical score of 1 or 2, then their clinical score at the time of discharge will be imputed as 2, which is the highest value for a non-hospitalized subject.

For time to event outcomes, subjects who are lost to follow-up or terminate the study prior to Day 29 and prior to observing/experiencing, the event will be censored at the time of their last observed assessment.

### Days of Non-invasive ventilation/high-flow oxygen:

If the subject’s 8 point ordinal scale is 5 at the last observed assessment, then the subject will be considered to be on non-invasive ventilation/high-flow oxygen through Day 29. The endpoint will be total days when assessments are available plus all imputed days following the last observed assessment.

If the subject is not on non-invasive ventilation/high-flow oxygen at the last observed assessment, then the subject will be considered to not be on non- invasive ventilation/high-flow oxygen for the remainder of follow-up. Thus, no additional imputed days will be added to the number of days recorded on available assessments.

### Days of invasive ventilation/ECMO:

If the subject’s clinical status scale is 6 or 7 at the last observed assessment, then the subject will be considered to be on ventilation/ECMO through Day 29. The endpoint will be total days when assessments are available plus all imputed days following the last observed assessment.

If the subject is not on invasive ventilation/ECMO at the last observed assessment, then the subject will be considered to not be on ventilation/ECMO oxygen for the remainder of follow-up. Thus, no additional imputed days will be added to the number of days recorded on available assessments.

### Days of Oxygen

If the subject’s 8 point ordinal scale is 4, 5, 6, or 7 at the last observed assessment, then the subject will be considered to be on oxygen through Day 29. The endpoint will be total days when assessments are available plus all imputed days following the last observed assessment.

If the subject is not on oxygen at the last observed assessment, then the subject will be considered to not be on oxygen for the remainder of follow-up. Thus, no additional imputed days will be added to the number of days recorded on available assessments.

### Days of Hospitalization

If the subject is discharged and no further hospitalization data are available, then the subject will be assumed to not have been readmitted. Thus, no additional imputed days will be added to the number of days recorded on available assessments. If a subject die while hospitalized, the number of days of hospitalization will be imputed as 28 days.

# References

1. Schoenfeld, D. 1981. The asymptotic properties of nonparametric tests for comparing survival distributions. Biometrika. 68 (1): 316–319.
2. Austin PC, Lee DS, Fine JP. Introduction to the Analysis of Survival Data in the Presence of Competing Risks. Circulation. 2016 Feb 9;133(6):601-9.
3. Jason P. Fine & Robert J. Gray (1999) A Proportional Hazards Model for the Subdistribution of a Competing Risk, Journal of the American Statistical Association, 94:446, 496-509
4. Drummond R. CONSORT Revised: Improving the Reporting of Randomized Clinical Trials. JAMA. 2001; 285(15):2006-2007.
5. Gamble, C., Krishan, A., Stocken, D., Lewis, S., Juszczak, E., Doré, C., …Loder, E. (2017). Guidelines for the Content of Statistical Analysis Plans in Clinical Trials. Journal of the American Medical Association, 318(23), 2337-2343. https://doi.org/10.1001/jama.2017.1855
