# Supplementary material for: Effect of Swine Glyco-humanized Polyclonal Neutralizing Antibody on Survival and Respiratory Failure in Patients Hospitalized With Severe COVID-19: A Randomized, Placebo-Controlled Trial
Source: Open Forum Infect Dis. 2023 Oct 20;10(11):ofad525. doi: 10.1093/ofid/ofad525 (PMC10629360; doi:10.1093/ofid/ofad525)
Supplement: ofad525_Supplementary_Data [file ofad525_supplementary_data.zip › Corrected_Supplementary 2_CLEAN.docx]

**Supplemental 2 Online Content**

**Effect of Swine glyco-humanized polyclonal neutralizing antibody on survival and respiratory failure in patients hospitalized with COVID-19 pneumonia: A Randomized Clinical Trial**

Benjamin Gaborit, MD, PhD^1,2^, Bernard Vanhove, PhD^3^, Karine Lacombe, MD, PhD ^4^, Thomas Guimard, MD^5^, Laurent Hocqueloux, MD^6^, Ludivine Perrier, PharmD^7^, Vincent Dubee, MD, PhD ^8,9^, Virginie Ferre, MD, PhD ^10^,Celine Bressollette, MD, PhD ^10^, Régis Josien, MD, PhD ^2,11^, Aurélie Le Thuaut, MSc^7,12^, Marie-Anne Vibet, PhD^7,12^, Alexandra Jobert, PhD^7,13^,Eric Dailly, PharmD, PhD^14^, Florence Ader, MD, PhD^15,16^, Sophie Brouard, PhD^2^, Odile Duvaux, MD, PhD^3^, François Raffi, MD, PhD ^1^ for the POLYCOR study group

**eTable 1.** Primary outcome of the POLYCOR trial for the as-randomized population (ITT) et per-protocol population, subgroup analyses on mITT

**eTable 2.** Post-infusion plasma concentrations of XAV-19

**eTable 3.** Adverse events according to grade (Safety population)

**eTable 4**. Grade 3-4 laboratory abnormalities (Safety population)

**eFigure 1.** Forest plot of Primary outcome in subgroups of analysis (mITT)

**eFigure 2.** Respiratory status according to 8-point scale during study

**eFigure 3.** Time to death

**eFigure 4.** Time to hospital discharge

**eFigure 5.** Evolution of SARS-Cov2 viral load over time, in overall population (a) and According to Baseline Antibody Status, antibody -positive group (b) and antibody -positive group (c)

**eFigure 6.** Change from baseline of SARS-CoV-2 viral load in patients with onset of symptoms between 0-6 days (a), 7-10 days (b), 11-14 days (c)

This supplemental material has been provided by the authors to give readers additional

information about their work.

# Supplemantary material : Figures and tables

e Table 1. Primary outcome of the POLYCOR trial for the as-randomized population (ITT) and per-protocol population, subgroup analyses (mITT)

| **Outcome** | **XAV-19**  **(N=203)** | **Placebo**  **(N=195)** | **Adjusted risk difference (95% CI)** | **Odds / Hazard ratio (95%CI)** |  |
| --- | --- | --- | --- | --- | --- |
| **Primary outcome, occurrence of death or respiratory failure through day 15, No (%)** |  |  |  |  |  |
| All randomized patients (ITT)^a^ | 54/200 (27.0) | 48/189 (25.4) |  | 1.05 (0.65-1.68) | 0.85 |
| Per Protocol population | 50/196 (25.5) | 47/188 (25.0) | -0.6 (-7; 6) | 0.97 (0.60-1.58) | 0.90 |
| **Subgroup analyses (mITT analysis)** |  |  |  |  |  |
| Duration of symptoms |  |  |  |  |  |
| ≤ 6 days | 18/46 (39.1) | 17/40 (42.5) |  | 0.87 (0.37-2.06) |  |
| 7-10 days | 28/106 (26.4) | 25/101 (24.8) |  | 1.07 (0.57-2.03) |  |
| ≥11-14 days | 7/47 (14.9) | 6/48 (12.5) |  | 1.27 (0.38-4.27) |  |
| Age |  |  |  |  |  |
| < 60 yr | 24/102 (23.5) | 21/89 (23.6) |  | 0.88 (0.41-1.90) |  |
| ≥60 yr | 29/97 (29.9) | 27/100 (27) |  | 1.06 (0.56-2.01) |  |
| Number of comorbidities |  |  |  |  |  |
| None | 13/51 (25.5) | 18/49 (36.7) |  | 0.59 (0.23-1.50) |  |
| 1. | 15/72 (20.8) | 11/70 (15.7) |  | 1.38 (0.48-3.91) |  |
| ≥2 | 25/71 (35.2) | 19/67 (28.4) |  | 1.34 (0.63-2.84) |  |
| Immunodepressive status (IS) |  |  |  |  |  |
| With IS | 47/186 (25.3) | 45/175 (25.7) |  | 0.94 (0.57-1.55) |  |
| Without IS | 6/13 (46.2) | 3/14 (21.4) |  | 5.72 (0.31-105.6) |  |
| News Score2 at baseline |  |  |  |  |  |
| 2-4 | 2/27 (7.4) | 8/35 (22.9) |  | 0.19 (0.03-1.17) |  |
| 5-6 | 10/56 (17.9) | 4/44 (9.1) |  | 2.29 (0.63-8.34) |  |
| >7 | 35/105 (33.3) | 34/101 (33.7) |  | 1.02 (0.54-1.92) |  |
| Oxygen flow at baseline |  |  |  |  |  |
| ≤ 4 L/min | 26/145 (17.9) | 29/145 (20.0) |  | 0.76 (0.40-1.44) |  |
| >4 L/min | 27/54 (50.0) | 19/44 (43.2) |  | 1.35 (0.60-3.04) |  |
| COVID serology at baseline |  |  |  |  |  |
| Negative serology | 20/56 (35.7) | 22/55 (40.0) |  | 0.86 (0.39-1.89) |  |
| Positive serology | 9/64 (14.1) | 6/69 (8.7) |  | 1.55 (0.51-4.73) |  |
| **Selected secondary outcomes (mITT)** |  |  |  |  |  |
| Oxygen-free days, median (Q1, Q3)^b^ | 21 (14 ; 24) | 21 (16 ; 24) |  |  | 0.78 |
| Use of non-invasive ventilation/high flow oxygen until  day 29, No of patients (%) | 47 (23.6) | 44 (23.3) | 0.92 (0.6 – 1.6) | -0.42 (-6.4 – 5.5) |  |
| Days of non-invasive ventilation/high flow oxygen up to day 29, median (Q1, Q3)^c,b d^ | 4 (2.0; 10.0) | 4 (2.0;7.25) |  |  | 0.95 |
| Use of invasive ventilation or ECMO until day 29, No of patients (%)^d^ | 30 (15.1) | 15 (7.9) | 2.06 (1.05 - 4.05) | 6.5 (2.2 – 11.0) |  |
| Days of invasive ventilation/ECMO up to day 29, median (Q1, Q3)^c,b^ | 24 (14.25; 29.0) | 23 (14.5;26.5 ) |  |  | 0.03 |
| Time to NEWS<2 or hospital discharge, median (Q1, Q3) ^d^ | 8 (7 ;9) | 7 (7 ;8) | 5 (-2 ; 12) | 0.75 (0.61- 0.92) |  |
| Death before day 29 No. (%)^e^ | 7/199 (3.5) | 3/189 (1.6) | -1.8 (-4; 0.4) | 2.12 (0.55-8.20) e |  |
| Thrombotic events, No of patients (%)^f^ | 8/199 (4) | 5 (2.6) |  |  |  |

^a^ Analysis included all randomized patients. Missing data (3 in XAV-19 group and 6 in Placebo group) were handled by multiple imputations methods. The data presented correspond to the complete cases.

^b^ Van Elteren test (a Wilcoxon test adapted for stratification factors).

^c^ The total number of days are the sum of all reported days, regardless of whether the days occur consecutively or in disjoint intervals. For patients who died or withdrew their consent before day 29 was imputed as 29 days.

^d^ Median time before event for patients who had the event.

^e^  The model using a frailty model to take into account the variability between centers did not converge. Then no random effects were included in the final model.

^f^ Overall, 17 thrombotic events were identified, 7 in the placebo group (1 arterial thrombosis and 6 pulmonary embolisms) and 10 in the XAV-19 group (3 arterial thromboses, 2 peripheral venous thromboses and 12 pulmonary embolisms).

eFigure 1. **Forest plot** Primary outcome in subgroups (mITT analysis)


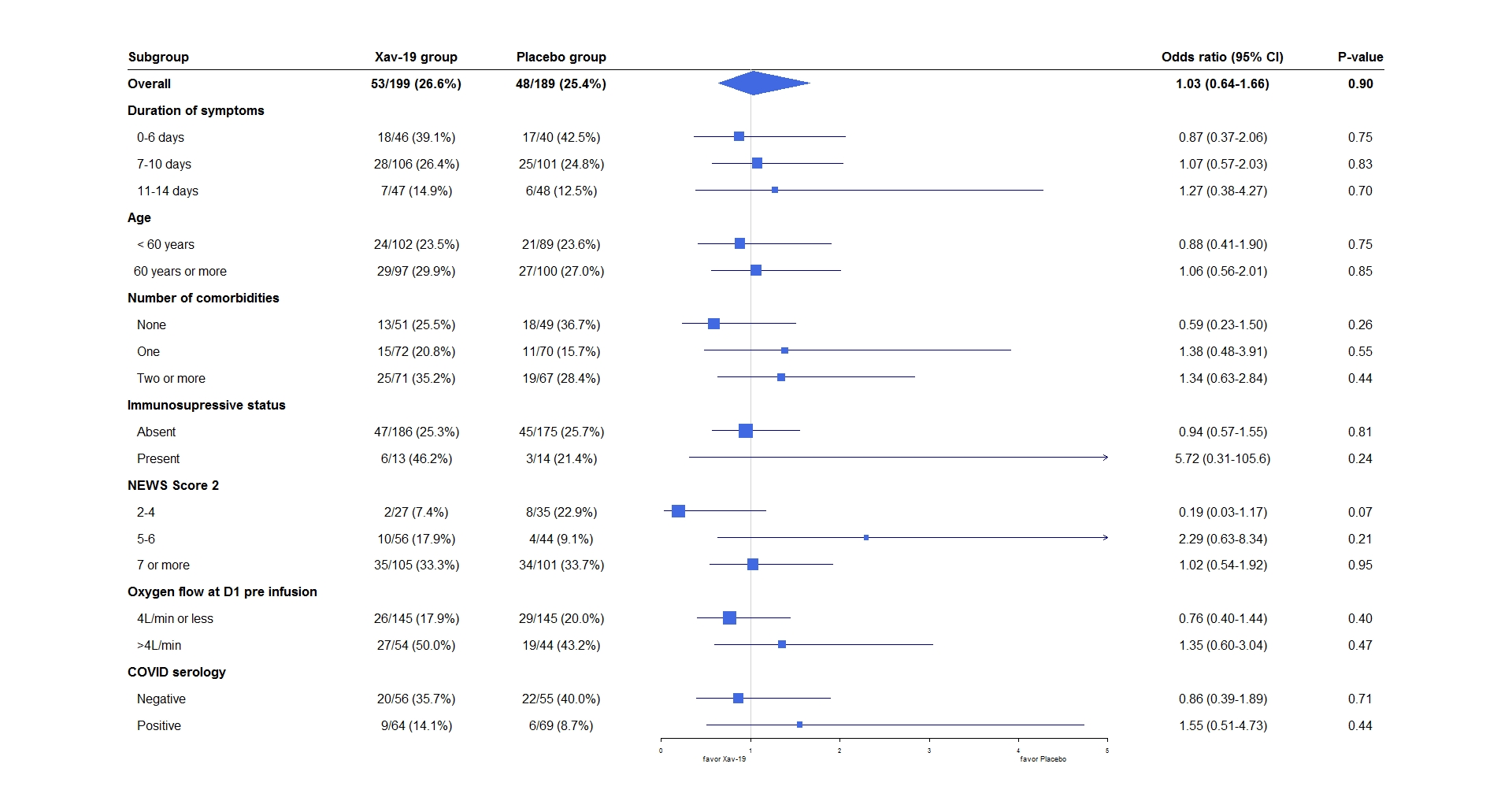


eFigure 2**.** Clinical status according to 8-point scale during study

**
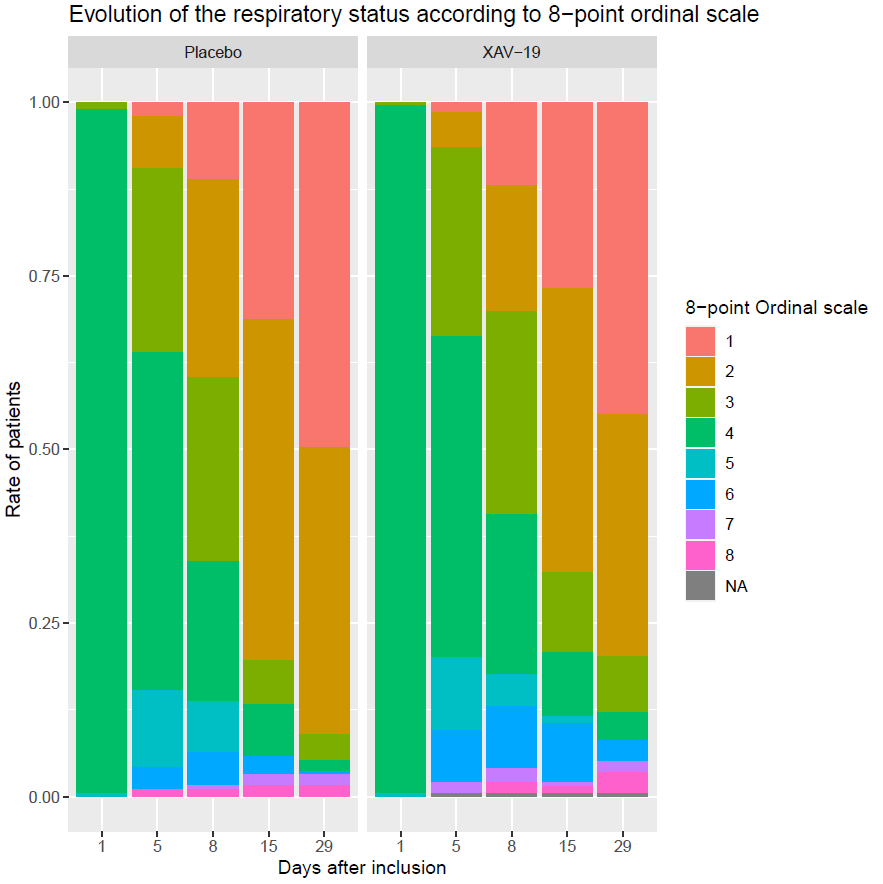
**

eFigure 3**.** Time to death

**
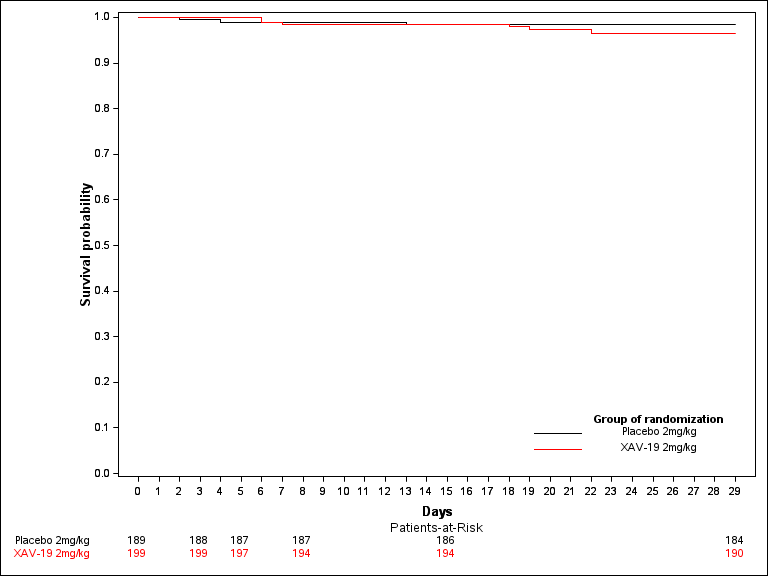
**

eFigure 4**.** Time to hospital discharge


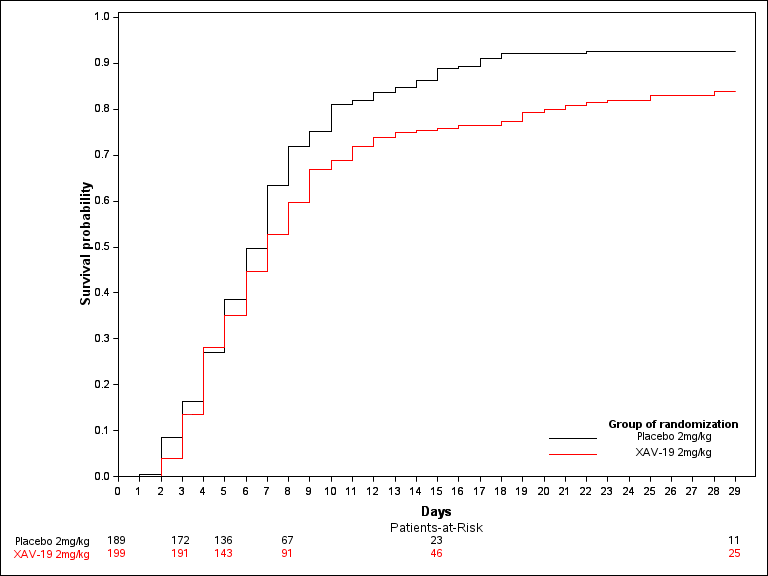


eFigure 5**.** Evolution of SARS-Cov2 viral load over time, in overall population (a) and according to baseline antibody Status, antibody -positive group (b) and antibody -negative group (c).

a


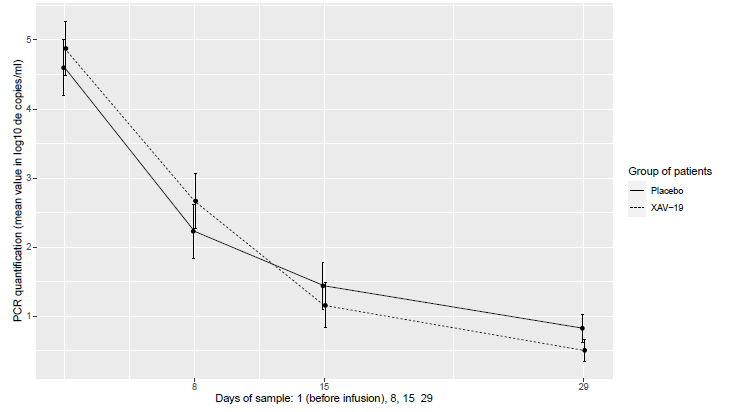

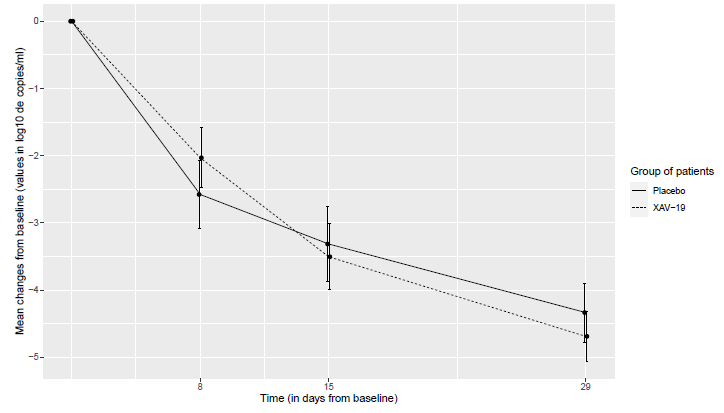


b


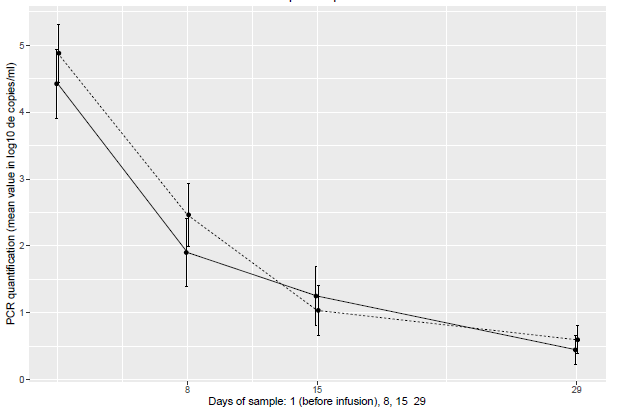

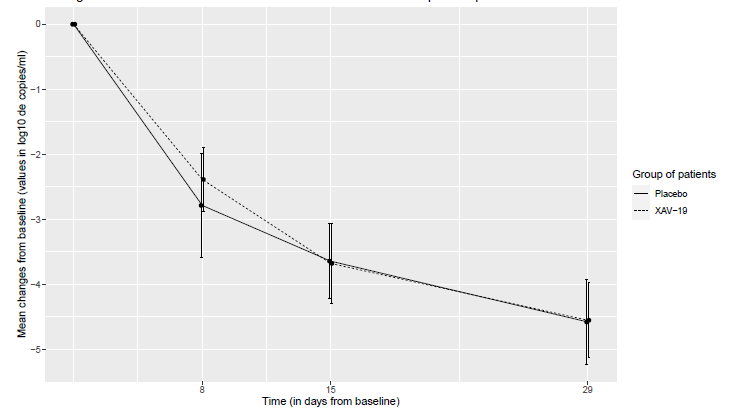


c


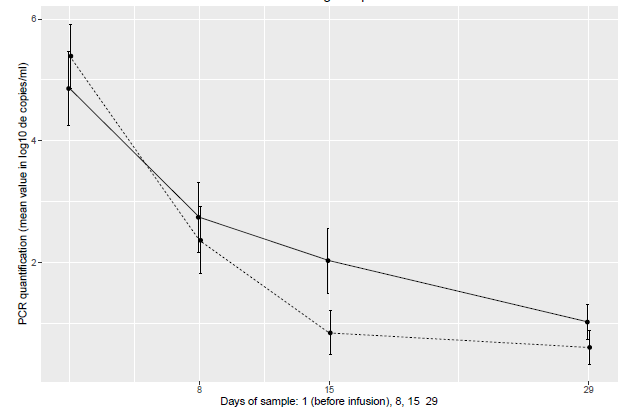
 **
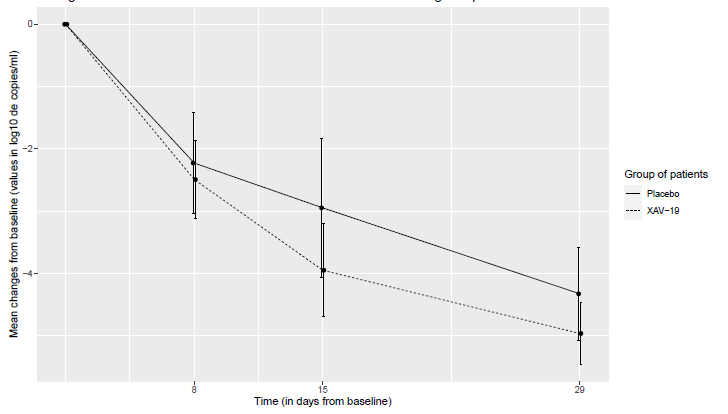
**

eFigure 6**.** Change from baseline of SARS-CoV-2 viral load in patients with onset of symptoms between 0-6 days (a), 7-10 days (b), 11-14 days (c)


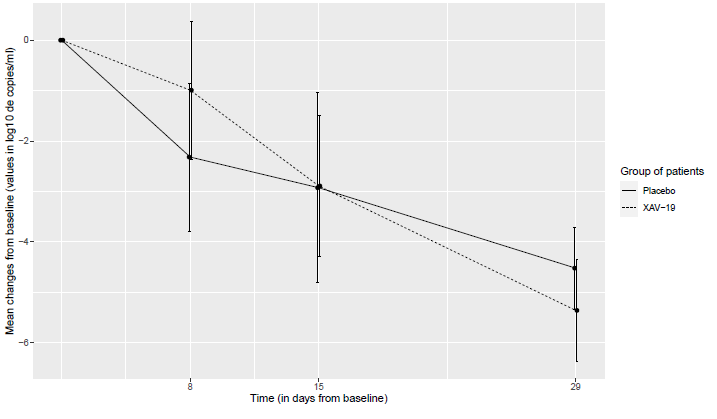


**
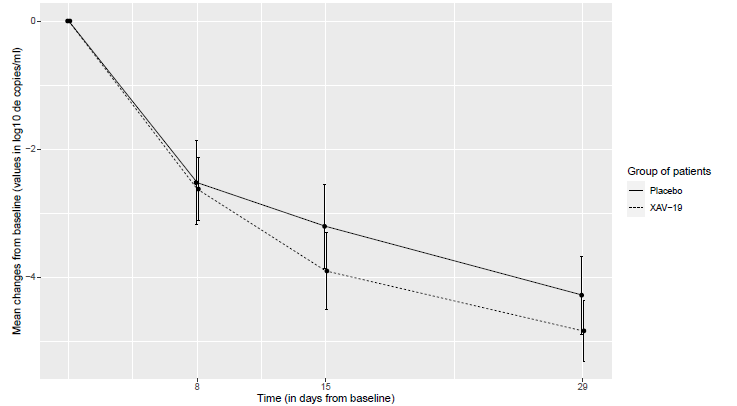
**

**
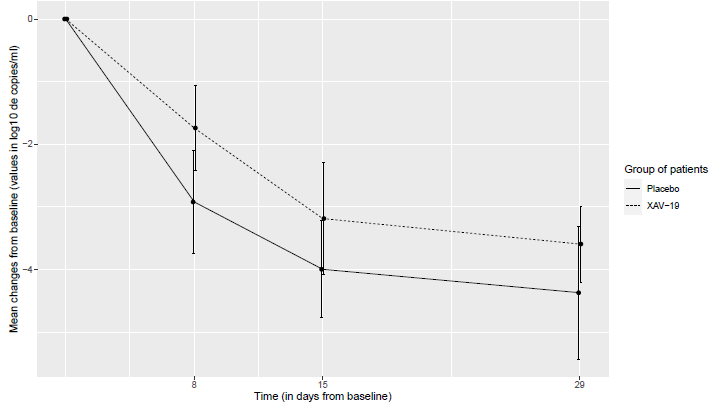
**

**eTable 2**. Post-infusion plasma concentrations of XAV-19

| Concentration | median (IQR), µg/ml [no]^a^ |
| --- | --- |
| Post day 1 infusion | 35.2 (34.3-37.6) [n=14] |
| at day 3 | 23.1 (20.6-27.6) [n=13] |
| at day 5^b^ | 17.7 (15.6-19.9) [n=14] |
| at day 8^a^ | 11.8 (11.1-14.2) [n=14] |
| at day 15 | 7.7 (7.2-9) [n=12] |
| at day 29 | 2.4 (2-2.5) [n=14] |

^a^ Number of patients analysed at each time.

^b^ At days 5 and 8, 14/14 and 12/14 patients, respectively, had serum concentrations above the target based on a target neutralization threshold of 10 µg/mL of XAV-19.

**eTable 3.** Adverse events according to grade (Safety population)

|  | XAV-19 (N=199) | |  |  |  |  | Placebo (N=190) | |  |  |
| --- | --- | --- | --- | --- | --- | --- | --- | --- | --- | --- |
| Adverse events by SOC (MedDRA V24.0) | Grade 1 | Grade 2 | Grade 3 | Grade 4 | Grade 5 | Grade 1 | Grade 2 | Grade 3 | Grade 4 | Grade 5 |
| Blood and lymphatic system disorders | 6 (30.0%) | 8 (40.0%) | 5 (25.0%) | 1 (5.0%) |  | 8 (29.6%) | 14 (51.9%) | 5 (18.5%) |  |  |
| Cardiac disorders | 7 (35.0%) | 7 (35.0%) | 3 (15.0%) | 3 (15%) |  | 10 (52.6%) | 5 (26.3%) | 3 (15.8%) | 1 (5.3%) |  |
| Ear and labyrinth disorders |  |  |  |  |  | 1 (50%) | 1 (50%) |  |  |  |
| Endocrine disorders | 1 (33.3%) | 2 (66.7%) |  |  |  | 1 (50%) | 1 (50%) |  |  |  |
| Respiratory, thoracic and mediastinal disorders | 12 (13.3%) | 19 (21.1%) | 22 (24.4%) | 29 (32.2%) | 8 (8.9%) | 10 (12.7%) | 20 (25.3%) | 22 (27.8%) | 22 (27.8%) | 5 (6.3%) |
| Gastrointestinal disorders | 28 (54.9%) | 19 (37.3%) | 3 (5.9%) | 1 (2.0%) |  | 28 (57.1%) | 14 (28.6%) | 6 (12.2%) | 1 (2.0%) |  |
| General disorders and administration site conditions | 28 (46.7%) | 23 (38.3%) | 8 (13.3%) | 1 (1.7%) |  | 18 (52.9%) | 13 (38.2%) | 2 (5.9%) | 1 (2.9%) |  |
| Hepatobiliary disorders | 18 (42.9%) | 16 (38.1%) | 7 (16.7%) | 1 (2.4%) |  | 13 (32.5%) | 17 (42.5%) | 9 (22.5%) | 1 (2.5%) |  |
| Immune system disorders |  | 1 (100%) |  |  |  |  |  |  |  |  |
| Infections and infestations | 13 (16.0%) | 33 (40.7%) | 24 (29.6%) | 10 (12.3%) | 1 (1.2%) | 5 (13.2%) | 14 (36.8%) | 12 (31.6%) | 7 (18.4%) |  |
| Injury, poisoning and procedural complications | 2 (33.3%) | 4 (66.7%) |  |  |  | 2 (33.3%) | 1 (16.7%) | 3 (50.0%) |  |  |
| Investigations | 7 (21.2%) | 5 (15.2%) | 20 (60.6%) | 1 (3.0%) |  | 5 (16.1%) | 7 (22.6%) | 12 (38.7%) | 5 (16.1%) | 2 (6.5%) |
| Metabolism and nutrition disorders | 27 (45.0%) | 24 (40%) | 9 (15.0%) |  |  | 21 (35.6%) | 32 (54.2%) | 6 (10.2%) |  |  |
| Musculoskeletal and connective tissue disorders | 8 (66.7%) | 4 (33.3%) |  |  |  | 8 (88.9%) | 1 (11.1%) |  |  |  |
| Neoplasms benign, malignant and unspecified (incl cysts and polyps) |  | 1 (100%) |  |  |  |  |  |  |  |  |
| Nervous system disorders | 18 (56.3%) | 9 (28.1%) | 3 (9.4%) | 2 (6.3%) |  | 10 (52.6%) | 7 (36.8%) | 1 (5.3%) | 1 (5.3%) |  |
| Psychiatric disorders | 10 (40.0%) | 13 (52.0%) | 2 (8.0%) |  |  | 11 (57.9%) | 7 (36.8%) | 1 (5.3%) |  |  |
| Renal and urinary disorders |  | 6 (100%) |  |  |  | 3 (27.3%) | 4 (36.4%) | 3 (27.3%) | 1 (9.1%) |  |
| Reproductive system and breast disorders | 1 (100%) |  |  |  |  |  |  |  |  |  |
| Skin and subcutaneous tissue disorders | 4 (36.4%) | 6 (54.5%) | 1 (9.1%) |  |  | 8 (72.7%) | 2 (18.2%) | 1 (9.1%) |  |  |
| Surgical and medical procedures |  |  |  |  |  |  |  | 1 (100%) |  |  |
| Vascular disorders | 10 (40.0%) | 6 (24.0%) | 6 (24.0%) | 3 (12.0%) |  | 4 (33.3%) | 6 (50.0%) | 1 (8.3%) | 1 (8.3%) |  |
| Total | 200 (34.5%) | 206 (35.5%) | 113 (19.5%) | 52 (9.0%) | 9 (1.6%) | 166 (35.5%) | 166 (35.5%) | 88 (18.8%) | 41 (8.8)% | 7 (1.5%) |

**eTable 4**. Grade 3-4 laboratory abnormalities (Safety population)

|  | **XAV-19 (N=199)** | **Placebo (N=190)** | **Total (N=389)** |
| --- | --- | --- | --- |
| **Adverse events by SOC and PT (MedDRA V24.0)** |  |  |  |
| Any serious adverse event-no. (%) | 14 | 15 | 29 |
| Blood and lymphatic system disorders | 6 | 5 | 11 |
| Anaemia | 3 | 1 | 4 |
| Anaemia macrocytic | 1 |  | 1 |
| Haemoglobin decreased |  | 1 | 1 |
| Iron deficiency anaemia | 1 |  | 1 |
| Normocytic anaemia | 1 |  | 1 |
| Red blood cell count decreased |  | 3 | 3 |
| Hepatobiliary disorders | 8 | 10 | 18 |
| Alanine aminotransferase increased | 4 | 4 | 8 |
| Aspartate aminotransferase increased | 2 |  | 2 |
| Blood bilirubin increased |  | 1 | 1 |
| Gamma-glutamyltransferase increased | 1 | 4 | 1 |
| Hepatocellular injury | 1 |  | 5 |
| Transaminases increased |  | 1 | 1 |
